# An Interlaboratory Comparison Study of Regulated and Emerging Mycotoxins Using Liquid Chromatography Mass Spectrometry: Challenges and Future Directions of Routine Multi-Mycotoxin Analysis including Emerging Mycotoxins

**DOI:** 10.3390/toxins14060405

**Published:** 2022-06-13

**Authors:** David Steiner, Armin Humpel, Eleonore Stamminger, Anna Schoeberl, Gerlinde Pachschwoell, Anita Sloboda, Christy Swoboda, Jolene Rigg, Dawei Zhang, Yahong Wang, Joshua Davis, Michael Sulyok, Rudolf Krska, Brian Quinn, Brett Greer, Christopher T. Elliott, Zbynek Dzuman, Jana Hajslova, Andreas Gschaider, Carina Fechner, Lisa Forstner, Elisabeth Varga, Piotr Jedziniak, Katarzyna Pietruszka, Adrianna Rudawska, Alexandra Malachová

**Affiliations:** 1 Romer Labs Diagnostic GmbH, Analytical Service Department, Technopark 5, 3430 Tulln, Austria; armin.humpel@dsm.com (A.H.); gerlinde.pachschwoell@dsm.com (G.P.); anita.sloboda@dsm.com (A.S.); 2 Romer Labs Division Holding GmbH, Marketing Department, Erber Campus 1, 3131 Getzersdorf, Austria; eleonore.stamminger@dsm.com (E.S.); joshua.davis@dsm.com (J.D.); 3 Romer Labs Division Holding GmbH, Technical Support Department, Technopark 5, 3430 Tulln, Austria; anna.schoeberl@dsm.com; 4 Romer Labs US Inc., Analytical Service Department, 1301 Stylemaster Drive, Union, MO 63084, USA; christy.swoboda@dsm.com (C.S.); jolene.rigg@dsm.com (J.R.); 5 Romer Labs Analytical Service (Wuxi) Ltd., No. 6-1 Chunyu Road, Wuxi 214000, China; dawei.zhang@dsm.com (D.Z.); yahong.wang@dsm.com (Y.W.); 6 Key Laboratory of Industrial Biotechnology, Ministry of Education, School of Biotechnology, Jiangnan University, Wuxi 214101, China; 7 Department of Agrobiotechnology (IFA-Tulln), Institute of Bioanalytics and Agro-Metabolomics, University of Natural Resources and Life Sciences, 3430 Vienna, Austria; michael.sulyok@boku.ac.at (M.S.); rudolf.krska@boku.ac.at (R.K.); 8 Institute for Global Food Security, Queens University Belfast, Belfast BT7 1NN, UK; brian.quinn@qub.ac.uk (B.Q.); brett.greer@qub.ac.uk (B.G.); chris.elliott@qub.ac.uk (C.T.E.); 9 Department of Food Analysis and Nutrition, University of Chemistry and Technology, Prague, Technicka 3, 16628 Prague 6, Czech Republic; zbynek.dzuman@vscht.cz (Z.D.); jana.hajslova@vscht.cz (J.H.); 10 LVA GmbH, Department for Residue Analysis, Magdeburggasse 10, 3400 Klosterneuburg, Austria; andreas.gschaider@lva.at (A.G.); carina.fechner@lva.at (C.F.); lisa.forstner@lva.at (L.F.); 11 Department of Food Chemistry and Toxicology, Faculty of Chemistry, University of Vienna, Währinger Straße 38-40, 1090 Vienna, Austria; elisabeth.varga@univie.ac.at; 12 Department of Pharmacology and Toxicology, National Veterinary Research Institute, 24-100 Pulawy, Poland; piotr.jedziniak@piwet.pulawy.pl (P.J.); katarzyna.pietruszka@piwet.pulawy.pl (K.P.); adrianna.rudawska@piwet.pulawy.pl (A.R.); 13 FFoQSI—Austrian Competence Centre for Feed and Food Quality, Safety & Innovation, FFoQSI GmbH, 3430 Tulln, Austria; alexandra.malachova@ffoqsi.at

**Keywords:** complex feed, certified reference material, internal standard, method harmonization, z-score

## Abstract

The present interlaboratory comparison study involved nine laboratories located throughout the world that tested for 24 regulated and non-regulated mycotoxins by applying their in-house LC-MS/MS multi-toxin method to 10 individual lots of 4 matrix commodities, including complex chicken and swine feed, soy and corn gluten. In total, more than 6000 data points were collected and analyzed statistically by calculating a consensus value in combination with a target standard deviation following a modified Horwitz equation. The performance of each participant was evaluated by a z-score assessment with a satisfying range of ±2, leading to an overall success rate of 70% for all tested compounds. Equal performance for both regulated and emerging mycotoxins indicates that participating routine laboratories have successfully expanded their analytical portfolio in view of potentially new regulations. In addition, the study design proved to be fit for the purpose of providing future certified reference materials, which surpass current analyte matrix combinations and exceed the typical scope of the regulatory framework.

## 1. Introduction

The worldwide occurrence of fungal species and their secondary metabolites known as mycotoxins are a major threat to global food and feed safety [1,2,3,4]. Mycotoxins are toxic to humans and animals and can cause acute and chronic diseases. Due to the diversity in their chemicals structure, their toxicity varies greatly, ranging from cyto-, nephron- and neurotoxin effects to carcinogenic, mutagenic, immunosuppressive and estrogenic effects [4,5]. Mycotoxicosis can be caused by the direct consumption of contaminated food and feedstuffs as well as by “carry over” into the food chain (e.g., via milk, animal tissue and eggs) [5]. 

The global population depends on starch and oilseed crops that are also inviting hosts for mycotoxin-producing fungi [6]. The progressing globalization of the food and feed market increases the challenges involved in tracing and monitoring these contaminants, which can result in major health concerns and barriers to international trade [6,7]. Additionally, changing climate conditions (temperature and humidity) can open new habitats for fungal species, which in turn can lead to the emergence of certain mycotoxins in geographical areas with no history of prior contamination and change mycotoxin patterns worldwide [6,8].

Mycotoxin-related health concerns have increased over the years. In order to control the contamination of food and feed by mycotoxins, many national and international institutions, such as the European Union (EU) and the Food and Drug Administration (FDA), have set maximum levels for the most common and potent mycotoxin–matrix combinations and, in addition, the World Health Organization (WHO) and the Food and Agriculture Organization (FAO) of the United Nations have developed strategies in order to mitigate mycotoxin contamination scenarios [4,5,7]. Regulations in the EU are based on the evaluation of risk assessments (evaluation of hazard and exposure) while taking agriculturally achievable levels in food- and feedstuffs into account as well [4]. As highlighted by Tittlemier et al. (2022), harmonization and verification strategies of standardized methods are of uppermost importance in order to guarantee a uniform application of regulations for mycotoxins [9].

In recent years, the coupling of high-pressure liquid chromatography (HPLC) to tandem mass spectrometry (MS/MS) has led to the development of highly sensitive and accurate methods for multi-mycotoxin analysis combined with short and simple extraction processes [4,7,10,11]. Moreover, multi-analyte LC-MS/MS methods can manage a high throughput of samples, making larger amounts of data available in a short amount of time [2]. LC-MS/MS multi-methods represent significant progress in food and feed analysis because of their ability to simultaneously monitor the compliance of mycotoxin concentrations with legal maximum values within a significantly reduced analytical turnaround time [7].

Therefore, worldwide operating laboratories are obliged to provide reliable and accurate results. To ensure high-quality operating levels, most laboratories base their workflow on ISO 17025:2017 (“General requirements for the competence of testing and calibration laboratories”), a standard published by the International Organization of Standardization. ISO 17025 lays out process requirements (validation of methods, sampling, handling of test and calibration items, etc.) as well as resource requirements (personnel, facilities and environmental conditions, metrological traceability, etc.) [12].

ISO 17025-accredited laboratories need to provide a validation for their analytical methods. Detailed requirements for method validation are listed in the standard [12]. ISO 17025-accredited laboratories are also required to ensure the validity of their results by recording data in such a way that trends are detectable, and by monitoring their performance, specifically the data trueness, by comparing their results with those of other laboratories by participating in proficiency testing (PT) or other interlaboratory comparison studies [12]. Organizations providing proficiency testing offer a broad variety of mycotoxin-contaminated matrices. However, these proficiency testing schemes typically focus on regulated mycotoxin–matrix combinations with a limited variability regarding complex matrices and emerging mycotoxins. As an example, De Girolamo et al. (2014) conducted such a proficiency testing study for mycotoxins with 18 participants from 10 countries, analyzing the regulated fungal metabolites aflatoxin B_1_, aflatoxin B_2_, aflatoxin G_1_, aflatoxin G_2_, deoxynivalenol, fumonisin B_1_, fumonisin B_2_, zearalenone, T-2 and HT-2 toxins, and ochratoxin A in maize and deoxynivalenol, zearalenone, T-2 and HT-2 toxins, and ochratoxin A in wheat with LC-MS/MS [3]. Another interlaboratory collaboration study was conducted by Sibanda et al. in 2021 by testing the applicability of diagnostic biochip arrays for 7 regulated mycotoxins in complex feed material, including, inter alia, dairy feed, dried distillers’ grains with solubles (DDGS), dog food and poultry feed [13]. Both studies demonstrated the applicability of either analytical reference methods as well as rapid test systems for regulated mycotoxins in common and challenging matrices. Although these results provide relevant information on the use of routine multi-mycotoxin methods, data on the applicability of analytical approaches exceeding the current regulatory scope are rather scarce. In addition, common PTs are focusing on a single target value per analyte–matrix combination and do not provide insights in the applicability of a method for a broad concentration range in a specific matrix commodity group.

The present interlaboratory comparison study includes nine (both accredited and non-accredited laboratories) from the USA, China and Europe and will provide some initial insights into the performance of multi-mycotoxin methods that go beyond common proficiency testing setups. This paper focuses on complex matrix materials such as chicken feed, swine feed, soy and corn gluten meal including 10 individual lots per matrix type. Furthermore, regulation candidates, such as emerging mycotoxins, were analyzed alongside regulated ones, as listed above, to evaluate and compare the performance of routine orientated laboratories applying multi-mycotoxin methods by means of LC-MS/MS.

## 2. Results

### 2.1. Homogeneity of the Sample Material

Evaluation of the sample homogeneity was conducted by comparing the between unit standard deviation, s_bu_, with the standard deviation of the interlaboratory comparison study (ILC) σ_p_ as well as the maximum between-unit variation u_bu_. The test material was considered to be adequately homogeneous if

s_bu_ ≤ 0.3 σ_p_ and u_bu_ ≤ 10%

The rationale for setting the factor 0.3 is that when this criterion is met, the standard deviation between samples will add less than about 10% to the variance in the performance assessment, so it is unlikely that the performance assessment will be affected [14]. All test materials passed the homogeneity test and were considered appropriate for the interlaboratory comparison study. The homogeneity study results are summarized in Table 1.

### 2.2. Summary of Reported Data

All participating laboratories tested for 11 regulated mycotoxins according to European Commission EC 1881/2006 [15]. In addition, two laboratories included 100% of target analytes (24) in their scope. Eight of ten laboratories included a scope between the regulated and several non-regulated toxins. A detailed overview of all tested compounds per participant is captured in Table S1.

The information content of the z-scores is influenced significantly by the number of reported data. Based on the international harmonized protocol for the proficiency testing of analytical chemistry laboratories, this number should not fall below 15 [3]. Otherwise, there would be serious limitations on the z-score, which is expressed as an increased statistical uncertainty on the consensus (represented by the standard error). This consensus value, which represents the best estimate of the true value, would be undesirably high and would correlate with a significant reduction of the z-score information content [3,16]. However, in order to increase the total number of evaluable data sets, laboratories with adequate measurement capacities for a reliable judgment of their reported results (only results higher than the limit of quantification (LOQ) were included for the statistical analysis. In addition, an outlier correction to the final data set was not conducted, as the overall data structure might have been substantially influenced by a significantly reduced number of statistically evaluable data points. Therefore, the final set of quantitative results for all matrix lots include a minimum of 6 and a maximum of 20 data points.

Of the reported quantitative results (6712) we conducted a statistical analysis for 6018 data points (90%). The distribution of positive findings for both regulated and non-regulated mycotoxins was equal. The highest positive rates for all analytes were observed for corn gluten samples (43%), followed by chicken feed (40%), swine feed (39%) and soy (17%). A summary of all reported data for the scope of each participant is listed in Table 2.

### 2.3. Contamination Patterns and Concentration Range

**Figure 1:**
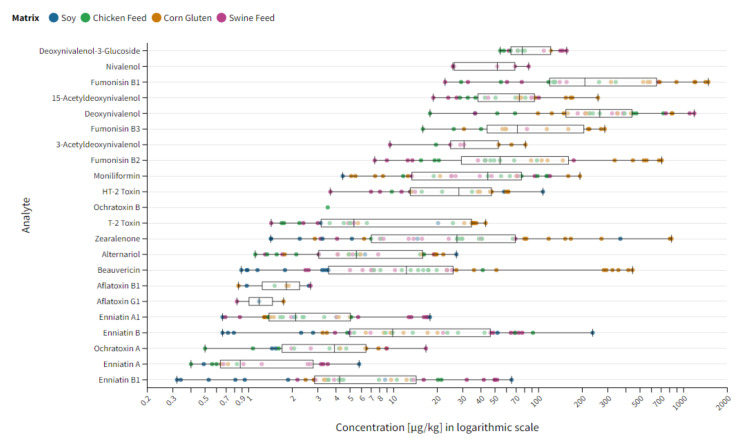
Overview of H15-mean values for all analyte matrix combinations. The x-axis represents the concentration range for the specific assigned values in µg/kg in a logarithmic scale. The y-axis shows the individual target compounds (AFB2 and AFG2 excluded).

Assigned values were calculated for 92% of analytes in at least one matrix lot. No assigned values were applicable for AFB2 and AFG2, as the number of quantitative results submitted for these compounds was too low to perform a statistical analysis. The most frequent number of assigned values was given for BEA with 40, followed by ZEN (37), ENN-B (37), ENN-B1 (36), DON (31), ENN-A1 (31), AOH (29), MON (29), FB2 (27), FB1 (26), T-2 toxin (25), 15-Ac-DON (22), HT-2 toxin (22), OTA (18), ENN-A (16), FB3 (14), D3G (13), 3-Ac-DON (9), AFB1 (7), NIV (5), AFG1 (3) and OTB (1). With 146 assigned values, corn gluten was the matrix with the highest number of evaluable statistical data points. This is followed by chicken feed with 142, swine feed with 139 and soy with 51 assigned values. An overview of H15 mean values for all analyte matrix combinations is depicted in Figure 1.

The lowest and highest observed assigned values were 0.32 and 1481 µg/kg for ENN-B1 and FB1, respectively, resulting in a concentration span of 4 orders of magnitude. Within the group of regulated mycotoxins, the broadest concentration span was observed for ZEN with a minimum assigned value of 1.42 µg/kg and a maximum of 824 µg/kg. This is followed by FB2 with 7.40–706 µg/kg as well as DON with 17.8–1184 µg/kg. In the category of non-regulated toxins, BEA showed the broadest concentration range from 0.89 to 444 µg/kg, followed by ENN-B with 0.66–235 µg/kg and ENN-B1 with 0.32–65.1 µg/kg. A detailed overview about the analyte-specific contamination range for each matrix commodity is listed in Table 2**.** In addition, a graphical illustration of the analyte matrix specific concentration range is covered by Figures S1–S4 and an overview of the analyte specific H15-mean based concentration ranges for each matrix commodity is captured within Tables S2–S5.

### 2.4. Overview of Total z-Score Performance

Matrix independency was observed for the performance of all tested mycotoxins. The overall acceptable z-scores were 70%, while the acceptable rate was equal for both groups of mycotoxins, including regulated and non-regulated toxins. In addition, 14% of z-scores were questionable and 16% unacceptable for all tested compounds. The best performance was observed for swine feed with 74% of acceptable z-scores followed by corn gluten with 71%, chicken feed with 67% and soy with 67%. With 12%, chicken feed was the matrix with the lowest number of total questionable z-scores but with 21% also representing the matrix with the highest number of unacceptable z-score results. On the other hand, with 13%, swine feed showed the lowest number of unacceptable results followed by corn gluten with 14% and soy with 17%. The highest number of questionable results was observed in soy with 16% followed by corn gluten with 15% and swine feed with 13%. A detailed description of analyte specific z-scores is provided in the Tables S6–S9.

**Figure 2:**
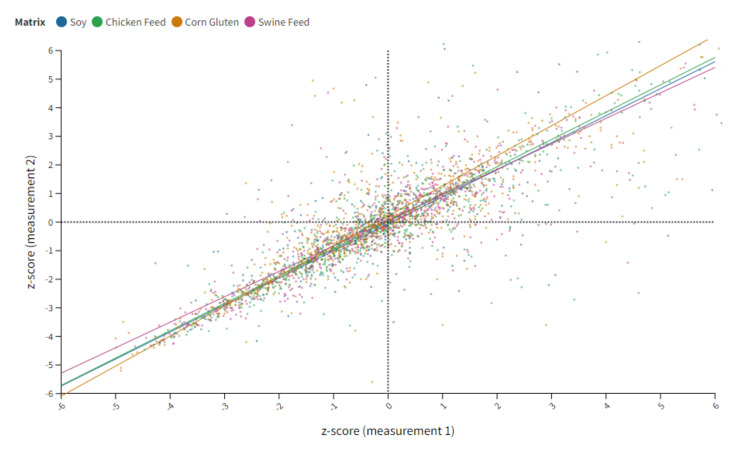
Quadrant chart of compiled z-score data for all analyte matrix combinations. The x-axis represents the z-score obtained from the first and the y-axis from the second data set. Each dot represents a z-score set for a specific analyte reported by the participants. The individual matrices are colored in blue for soy, green for chicken feed, brown for corn gluten and purple for swine feed.

A graphical overview of all z-score data calculated in this study is depicted in Figure 2. In this graphic, the replicate measurements (*n* = 2) for each lot are opposed and a product-moment (Pearson) correlation was conducted. The Pearson correlation is expressed as *r* and reflects the strength of the linear relationship of continuous variables (*x* and *y* vary together). Based on that, a very high correlation (size of *r* between 0.90 and 1.00) was obtained for all matrices with a correlation coefficient of 0.98 for soy, 0.95 for chicken feed and 0.90 for both corn gluten and swine feed [17]. With a Pearson correlation coefficient of 0.97, the 1439 data points for compounds currently subject to regulation have greater consistency than non-regulated substances; these had a Pearson correlation of 0.92 at 1506 data points. Less routine measurement of this substance class could be a possible reason for the lower consistency in repeated analysis. Furthermore, the outcome of the correlation analysis additionally proves the homogeneity of the sample material and highlights the consistency in replicate measurements.

### 2.5. Overview of Individual Laboratory Performance

#### 2.5.1. Soy Matrix

**Figure 3:**
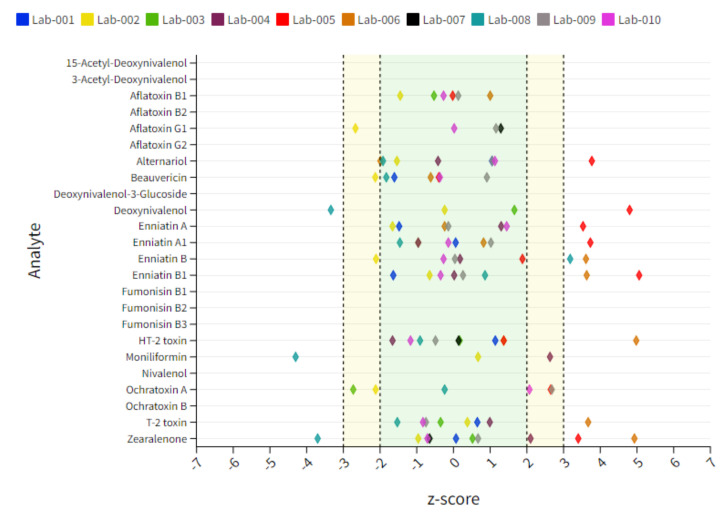
Dot plot chart representing an overview of individual lab performances expressed as a mean z-score derived from 10 tested soy samples. The x-axis represents the z-score, and each colored diamond reflects the individual participant. The y-axis represents the analytes included in the scope. The target acceptable z-score range of ±2 is marked with a green area.

Soy samples contain the lowest number of positive findings and thus the lowest number of statistical evaluable data points. Arrows pointing vertically up or down indicate unacceptable results, while arrows pointing at a slight angle indicate questionable results. The target z-score range of ±2 as the criterion for successful participation was not reached for BEA (↘) and ENN-B (↘) for Lab 2 showing a slight underestimation of these compounds. Lab 4 showed, in contrast, a trend in overestimating BEA (↑) as well as a minor overestimation of ZEN (↗), whereby two of ten reported results are the main reason for the deviation of the target z-score. A clear overestimation was shown for ENN-Bs (↑) and ZEN (↑) for Lab 6. Some laboratories also show questionable or unacceptable results for several compounds as in the case of Lab 2, Lab 5, Lab 6 and Lab 7 for AFB1, AFG1, AOH, DON, enniatins (ENNs), HT-2, T-2 or ZEN. However, in these cases, only a small number of data points were given, and therefore a clear statement to the analyte–matrix-specific performance cannot be made. Therefore, a sum interpretation was only conducted when a minimum of 6 reported z-score data points were available. An overview of the individual lab performance based on average z-score values is depicted in Figure 3**.** An overview of all z-score data including those which exceed the range of ±7 is captured in Table S6.

#### 2.5.2. Corn Gluten Matrix

**Figure 4:**
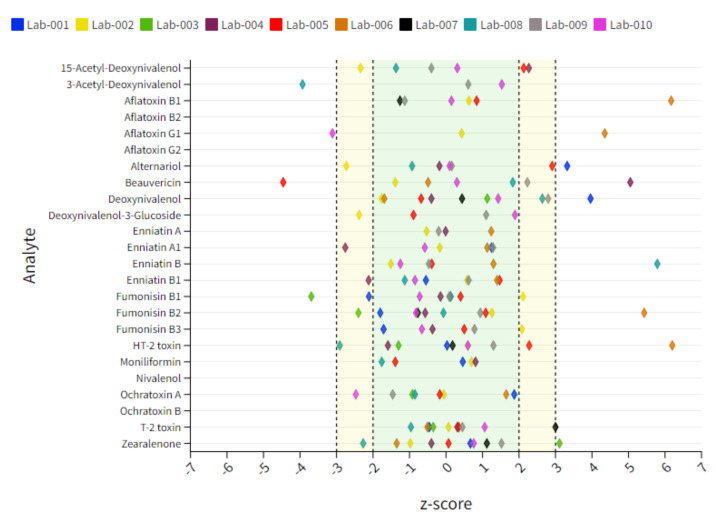
Dot plot chart representing an overview of individual lab performances expressed as mean z-score derived from 10 tested corn gluten samples. The x-axis represents the z-score and each colored diamond reflects the individual participant. The y-axis represents the analytes included in the scope. The target acceptable z-score range of ±2 is marked with a green area.

As stated in Table 2**,** corn gluten samples contained the highest contamination rate of all matrices. Deviations from the acceptable z-score range for regulated mycotoxins were observed for fumonisins (FBs), DON, OTA, HT-2, T-2 and ZEN. In addition, questionable and unacceptable findings were made for non-regulated mycotoxins, including Ac-DONs, AOH, BEA and ENNs. Lab 1 showed a slight underestimation for FB1 (↘) as well as a clear overestimation for AOH (↑) and DON (↑). Minor underestimations were also true for 15-Ac-DON (↘) and AOH (↘) for Lab 2 as well as a slight overestimation for FB1 (↗). Unacceptable results were obtained for FB1 (↓) for Lab 3 and questionable results for FB2 (↘). In addition, Lab 3 showed a clear overestimation of ZEN (↑), although this outcome was mainly influenced by 4 data points out of 20. Significant overestimations were observed for BEA (↑), ENN-B (↑) and OTA (↑) for Lab 4. Questionable results for this lab were obtained for 15-Ac-DON (↗), ENN-A1 (↘) and ENN-B1 (↘). Lab 5 showed a trend in slightly overestimating 15-Ac-DON (↗) and AOH (↗) as well as in systematically underestimating BEA (↓). Unacceptable results were obtained for FB1 (↑) and FB2 (↑) as well as HT-2 (↑) for Lab 6 and for T-2 (↑) for Lab 7. Lab 8 delivered questionable results for DON (↗), HT-2 (↘) and ZEN (↘), although data for DON exhibited a significant spread and did not show any consistency in the measurement. Unacceptable results were additionally observed for 3-Ac-DON (↓) and ENN-B (↑) for this lab. Minor deviations were obtained for Lab 9 and Lab 10. Only slight overestimations were obtained for Lab 9 for BEA (↗) and DON (↗), and a minor underestimation was observed for OTA (↘) for Lab 10 in this matrix type. The individual lab performance based on average z-score values is depicted in Figure 4**.** An overview of all z-score data including those which are exceeding the range of ±7 is captured in Table S7.

#### 2.5.3. Chicken Feed Matrix

**Figure 5:**
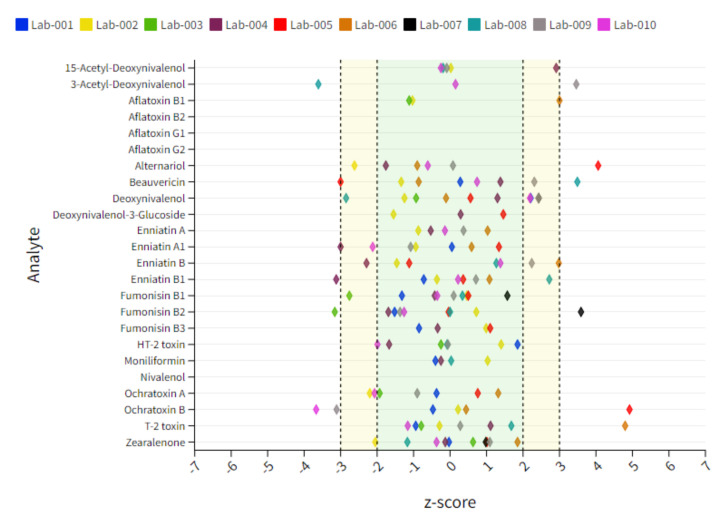
Dot plot chart representing an overview of individual lab performance expressed as mean z-score derived from 10 tested chicken feed samples. The x-axis represents the z-score, and each colored diamond reflects the individual participant. The y-axis represents the analytes included in the scope. The target acceptable z-score range of ±2 is marked with a green area.

Regarding any deviations from the target z-score range of ±2 for both regulated and non-regulated toxins, there were similar findings for complex chicken feed samples, compared to corn gluten. Questionable and unacceptable results were obtained for DON, FBs, OTA and T-2 for regulated and for AOH, BEA and ENNs for non-regulated mycotoxins. Questionable results were obtained for DON (↗) for Lab 1 and for AOH (↘) and OTA (↘) for Lab 2 as well as for FB1 (↘) for Lab 3. In addition, Lab 3 showed unacceptable average z-scores for FB2 (↓), indicating a general underestimation for FBs. Underestimations were also observed for ENNs in general for Lab 4, including ENN-A1 (↓), ENN-B (↘) and ENN-B1 (↓) and a clear overestimation for OTA (↑). Unacceptable results were also recorded at Lab 5 for AOH (↑) and BEA (↓) as well as for ENN-B (↑), FB1 (↑), FB2 (↑) and T-2 (↑) at Lab 6. Lab 7 reported questionable results for DON (↗) and unacceptable results for FB2 (↑). Additionally, unacceptable z-score data were submitted by Lab 8 for AOH (↑), BEA (↑), ENN-A1 (↑) and OTA (↑). In addition, Lab 8 delivered questionable results for DON (↘) and ENN-B1 (↗). Lab 9 delivered questionable results for BEA (↗), DON (↗) and ENN-B (↗). However, in all cases, the deviation from the target z-score range was mainly influenced by two significantly enhanced results from a data set including a minimum of 12 and maximum of 18 data points per analyte/matrix combination. Lab 10 provided data for DON (↗) showing a slight overestimation for this compound, which was also mainly affected by 2 of 14 submitted results. The individual lab performance based on average z-score values is depicted in Figure 5. An overview of all z-score data including those exceeding the range of ±7 is captured in Table S8.

#### 2.5.4. Swine Feed Matrix

**Figure 6:**
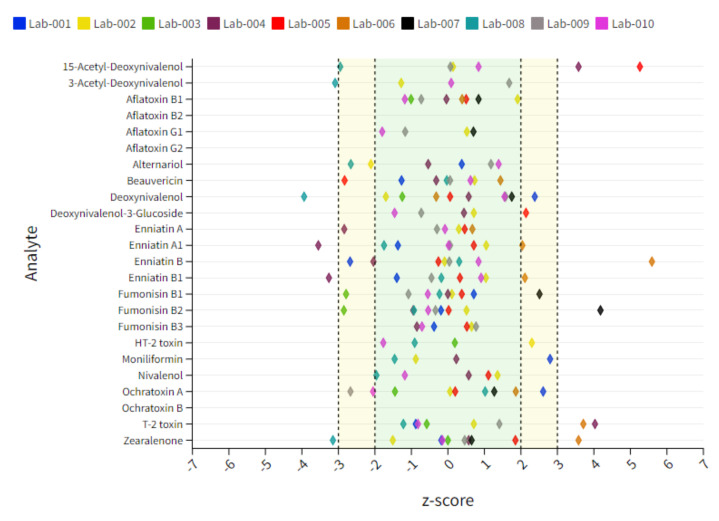
Dot plot chart representing an overview of individual lab performance expressed as mean z-score derived from 10 tested swine feed samples. The x-axis represents the z-score and each colored diamond reflects the individual participant. The y-axis represents the analytes included in the scope. The target acceptable z-score range of ±2 is marked with a green area.

In swine feed, the majority of non-acceptable results was observed for DON and FBs from the group of regulated mycotoxins and for 15-Ac-DON, AOH and ENNs from the group of non-regulated toxins. Data submitted by Lab 1 showed slight overestimations for DON (↗), MON (↗) and OTA (↗) as well as trends in underestimating ENN-B (↘). Minor underestimations were also observed for AOH (↘) and HT-2 (↗) at Lab 2 and for FB1 (↘) and FB 2 (↘) at Lab 3. Lab 4 delivered unacceptable results for 15-Ac-DON (↑), ENN-A1 (↓), ENN-B1 (↓) and T-2 (↑) as well as questionable results for ENN-A (↘). Questionable results were also observed for BEA (↘) and unacceptable for 15-Ac-DON (↑) and AOH (↑) at Lab 5. Significant overestimations were observed for ENN-B (↑), FB1 (↑), FB2 (↑) and ZEN (↑) as well as a slight overestimation for ENN-B1 (↗) at Lab 6. Data reported for FB1 (↗) showed a slight, and for FB2 (↑) a significant, overestimation of these compounds at Lab 7. Lab 8 provided questionable data for 15-Ac-DON (↘) and AOH (↘) and unacceptable z-scores were recorded for 3-Ac-DON (↓), DON (↓) and ZEN (↓). Lab 9 only provided questionable results for OTA (↘). No deviations from the satisfactory z-score range of ±2 was observed for Lab 10. The individual lab performance based on average z-score values is depicted in Figure 6**.** An overview of all z-score data including those exceeding the range of ±7 is captured in the Table S9.

## 3. Discussion

### 3.1. Contamination Pattern

The overall contamination pattern revealed a high exposure of *Fusarium* toxins, in particular ENNs, FBs, BEA, MON, DON and ZEN, leading to the conclusion that *Fusarium* spp. was the dominant fungal species in all tested samples [18,19,20]. Minor exposure scenarios were given for *Aspergillus* spp. and *Penicillium* spp. in general. However, some lots of all four matrix commodities also contained traces of aflatoxins (AFLAs) and OTA, indicating an infection with the respective fungal species as well. As highlighted within Section 2.3, ZEN was the most prevalent compound in the entire sample set, which can be related to the matrix commodities included in the study. ZEN is ubiquitous in a broad range of different feed commodities as recently revealed in a review conducted by Ropejko and Twaruzek [21]. In 93.3% (28/30) of swine feed samples, ZEN was found in concentrations between 8.93 and 866 µg/kg. In addition, 69% (9/13) of soy meal samples tested positive for ZEN with a mean value of 51 µg/kg. Considering the presence of ZEN in grain-based commodities, the occurrence ranged from 21 to 100% of tested corn and 1.9 to 63% of tested wheat material. Since these two grain materials along with soy comprise the main components of chicken feed, positive findings of ZEN in this complex feed material are to be expected [10]. Aside from high potential contamination scenarios for ZEN, a high method sensitivity in all participating laboratories was given for this compound, as the limits of quantification ranged from 0.6 to 25 µg/kg. High method sensitivity was also true for BEA, the most prevalent non-regulated mycotoxin in this study. Limits of quantification for BEA ranged from 0.1 to 4.0 µg/kg for the methods of the participants who included this compound in their scope. In general, the prevalence of regulated and non-regulated toxins (see Section 2.3) was balanced with a slight weighting for regulated mycotoxins when taking the total number of statistical evaluable data points compared to the amount of measured toxins into account (268/237 average data points per regulated/non-regulated mycotoxins). However, this remarkable number of positive findings for non-regulated toxins emphasizes the need to expand current analytical mycotoxin methods in order to gain more information on total contamination patterns. The findings of this study also confirm previously gained knowledge about the contamination rate of emerging mycotoxins. Gruber-Dorninger et al. described in their 2017 overview of emerging mycotoxins that BEA was detected in 98% of feed samples and feed raw materials (*n* = 83) and in 54% of unprocessed grains (*n* = 861). A similar contamination picture emerged for ENNs; 96% of feed samples (*n* = 83) and 76% of unprocessed grains (*n* = 2647) tested positive for this compound group [22,23]. Based on the potential toxic impact and on the occurrence of some selected emerging mycotoxins covered in the paper, they derived a ranking list prioritizing the research focus for the scientific community. With respect to the compounds included in our study, the list starts from the bottom with ENNs and BEA. While these compounds show a clear toxic impact in vitro, their impact in vivo is, according to the current knowledge, minor [23]. This is followed by AOH, which shows genotoxic effects in vitro, but these effects could not be confirmed in vivo so far. On the upper end of the list, MON can be found, as its toxicity and occurrence pose a clear risk to poultry in particular. To complete the list of emerging mycotoxins not covered by our study, the future focus should additionally be set on culmorin < butenolide < sterigmatocystin < alternariol monomethyl ether and tenuazonic acid [23].

### 3.2. Matrix-Dependent Deviations

The reasons for the deviations of the target z-score are manifold, including, mainly, the signal suppression or enhancement effects coming from the matrix material (impact of co-eluting co-extracts on ESI ionization process) and low extraction efficiencies. As highlighted by Martinez-Dominguez et al. in 2016, matrix suppressions (>20%) in soy isoflavone supplements obtained from soy material were observed for 98% of tested compounds including 257 pesticides and mycotoxins. However, extraction procedures either based on dilute and shoot or QuEChERS (quick, easy, cheap, efficient, rugged, safe) ensured recoveries of 72% and 66% of the compounds in a range of 70% to 120% [24]. These results indicate that matrix effects may have a higher influence on analytical performance for this matrix, compared to extraction efficiency. As a consequence, those carrying out the tests may attempt to compensate for this with improper recovery corrections; this is a common overreaction when no internal standards are used or applicable.

Compared to soy, corn gluten samples resulted in a higher rate of questionable and unacceptable results, which can be linked to the overall higher contamination rate and positive findings submitted by the participants. However, corn gluten as such can be considered a challenging matrix, as typical mycotoxin multi-methods have been deemed insufficient for the analysis of gluten-based material, including corn gluten meal, corn gluten feed and DDGS (distiller’s dried grains with solubles). Therefore, these matrices often require a specific clean-up prior to analysis due to the origination of various Maillard (non-enzymatic reaction between reducing sugars and amino acids, peptides or proteins) effects [25] potentially interfering with products upon heat treatment conditions [26]. Another phenomenon related to this matrix type is based on the ratio between the different FBs. Within the fumonisin family, FB1 is typically the most prevalent, followed by FB2 and FB3, which are usually associated with lower concentrations [27]. As highlighted within a comprehensive co-occurrence study conducted by Kovalsky et al. in 2016, the concentration ratio for FBs B1:B2:B3 is 7:3:1 for the 75th percentile of the data set used, including 1113 finished feed, maize and silage samples from 46 countries. Taking the maximum concentration into account, this ratio is shifting towards a higher FB1 content and results in a concentration ratio of 10:2:1 for FBs B1:B2:B3 [28]. These results indicate that the FB1 content in naturally contaminated samples is about 10 times higher compared to FB3 and between a factor of 2 to 3 higher compared to FB2. However, assigned values of the corn gluten samples derived from this study revealed a different contamination ratio for FBs. Samples with a higher degree of contamination (>1 mg/kg total FBs) resulted in a concentration ratio of 5:2:1 for FBs B1:B2:B3; less contaminated samples (<1 mg/kg total FBs) resulted in a ratio of 10:4:1 for FBs B1:B2:B3. A possible reason for this variation in the FBs ratio could be based on a degradation of FBs during the production process of corn gluten. Corn gluten itself is a coproduct of corn wet milling processes, which separates corn kernels into hull, germ, gluten and starch. Among different coproducts from the wet milling process, such as corn oil and gluten meal, starch can be further processed into ethanol, which many regard as the most high-value product of the corn kernels [29]. In 2001, Saunders et al. indicated that commercial procedures for converting corn into feed and food products, including processes such as wet milling or extrusion, significantly reduces the FBs concentrations in the final products [30]. These findings were also confirmed by Prettl et al. in 2011. Wet milling methods of processing cornstarch led to a reduction in FB1, as it is soluble in water. Reciprocally, dry milling processes of corn did not affect the FB1 content, as a distribution of this compound into bran, flour and germ was observed [31].

Observed similarities in terms of laboratory performance were given for complex feed matrices compared to corn gluten. This is probably related to the higher overall positive rate in these matrices. In addition, chicken and swine feed also contain ingredients, such as soy, DDGS, corn or rapeseed, which also may negatively contribute to overall lab performance [10,32]. As highlighted by Steiner et al. in 2020, matrix effects greater than 20% in complex chicken and swine feed matrices were observed for 39% and 42% of 100 tested compounds, including mycotoxins, pesticides and veterinary drugs. Strong matrix effects (>20%), signal suppressions in particular, were also observed for feed ingredients such as DDGS, rapeseed and corn. By achieving acceptable extraction efficiencies for both complex matrices and raw materials, both absolute and, in particular, relative matrix effects were revealed to be the major obstacle for the performance of a multi-class LC-MS/MS-based method [10]. Due to the high degree of heterogeneity of complex feedstuff, it is very likely that recovery corrections of the final results are doomed to fail for methods lacking internal standards, as there is no uniform feed formula existing.

### 3.3. Matrix-Independent Deviations

As highlighted in the previous section, deviations from an acceptable z-score are mainly related to matrix-specific characteristics that hamper analytical performance. However, by compiling all matrix data together, general tendencies for some compounds were revealed. An overview of analyte-specific z-score deviations from the target range of ±2 is provided in Table S10. For the interpretation of matrix-independent analyte specific trends, a sum product expressed as a percentage was derived from the individual z-score deviations per matrix/analyte combination to ensure a weighted average value. As regards regulated toxins, general tendencies for unsatisfactory results, and thus a matrix-independent performance, were observed for DON (33% deviations of all reported z-scores) followed by OTA (32%), FB2 (31%) and FB1 (30%). The trend for DON was clearly toward an overestimation whereby the over- and underestimating events for the remaining regulated toxins were equally distributed. Similar trends were also observed for non-regulated toxins including AOH (47%), 15-AcDON (38%), ENN-B (32%), BEA (31%) and 3-AcDON (25%). Tendencies in general overestimations were observed for 15-AcDON and ENN-B, while deviations for 3-AcDON showed a trend in underestimation. The remaining non-regulated toxins AOH and BEA were equally affected by over- and underreporting. These results for both groups of mycotoxins indicate that a few approaches show some limitations in terms of adequate extraction of the target compound from the matrix material and additionally in reducing or compensating unwanted matrix effects. For regulated mycotoxins in particular, matrix effects could be easily handled by applying internal standards. In order to reduce interferences from the matrix material and provide more certainty in manual data integration, proper clean-up strategies (e.g., clean-up columns) should be considered and applied if possible.

### 3.4. Internal Standard vs. Recovery Correction

As revealed in the previous section, accurate quantification can be significantly hampered by matrix effects; for this reason, the use of isotopically labelled internal standards should be considered both for routine-orientated laboratories and in particular for those working within an accredited environment [33]. Especially for challenging matrix materials, such as complex chicken or swine feed, the use of internal standards could overcome these effects by compensating for signal suppressions and enhancements that occur during the ionization process [10,34]. A very economic and efficient way to apply these standards is the so-called SIDA approach (stable isotope dilution assay), in which the internal standards are added at the end of the sample preparation. One major advantage of this approach is that it requires only very small amounts of an internal standard mixture. This ensures that matrix effects are corrected efficiently by keeping the costs per analysis low [26,33]. Alternative strategies, such as matrix-matched calibrations, are feasible and inexpensive, although they have significant shortcomings in routine settings, as multiple-matrix calibrations must be analyzed in one sequence [26]. Within our study, five laboratories used uniformly [^13^C]-labelled standards for each of the 11 regulated toxins for which they also possessed an accredited status.

**Figure 7:**
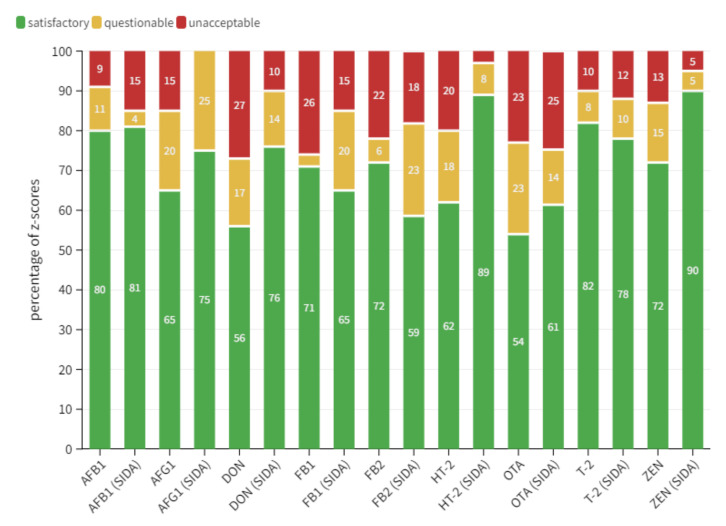
Bar chart comparison between z-score performance of regulated toxins for laboratories applying a recovery correction to the measured result and laboratories applying an internal standard correction by following a stable isotope dilution assay. Data provided represent an average z-score of all tested matrices. The x-axis shows all regulated mycotoxins; abbreviations including parenthetical note “(SIDA)” represent data corrected by internal standards. The y-axis represents the percentage of z-scores. Satisfactory, questionable and unacceptable results are colored as green, yellow and red, respectively.

As depicted in Figure 7, the use of internal standards also benefitted overall performance and resulted in a higher number of satisfactory z-scores. This was particularly true for two of the most prevalent regulated mycotoxins, DON (481 z-scores) and ZEN (615): the difference between recovery corrected and data corrected with internal standards was around 20% for both compounds. Even more significant was the result obtained for HT-2 (254) with a difference of 27%. Smaller differences were observed for OTA (232) with 7% higher acceptable z-scores for SIDA approaches and 4% for T-2 (363) for recovery corrected approaches. However, in both cases, the difference cannot be considered as significant. Furthermore, no significant data were observed for AFB1 (71); SIDA methods only displayed beneficial results in 1% of cases. Differences for AFG1 (24) were higher (10% more acceptable z-scores using SIDA), but in this case, the number of z-scores was so low that a clear statement is impossible. Results for both fumonisins showed a different pattern in terms of method performance. Satisfactory z-score rates were higher by 13% and 6% for FB1 (476) and FB2 (427), respectively. However, these results were mainly influenced by data provided from Lab 3 following a sample preparation protocol with a very short extraction period of 30 min. Obviously, this extraction time is insufficient to properly extract fumonisins from the matrix material; Meneely et al. demonstrated in 2011 that sample extraction periods invariably include tedious extraction times between 60 and 90 min [35]. However, the overall results indicate that SIDA-based approaches provide high reliability and broad applicability, even for complex matrices. This is raised by the fact that the number of questionable results for FBs based on SIDA approaches was much higher and the number of unacceptable results was significantly lower compared to recovery corrected methods. An overview of individual z-score data for regulated mycotoxins corrected either by internal standards or recovery is depicted in Tables S6–S9.

## 4. Conclusions

Based on the results of this interlaboratory comparison study, we can derive the following conclusions:

- An overall value of 70% for satisfactory z-score results within ±2 proves that all participating laboratories delivered accurate data which are fit for purpose for official control of regulated toxins as well as emerging mycotoxins, even in complex matrix material. The applied methods also proved their applicability in a broad concentration range, from high to trace contaminations [3].
- There is broad consensus in terms of sample preparation strategies as the majority of participants used an acetonitrile-based water mixture under acidic conditions. Therefore, this sample preparation protocol can be seen as the most suitable compromise for multi-mycotoxin methods.
- Diverse and broad contamination patterns for both regulated and emerging mycotoxins, such as BEA and ENNs, provide a relevant basis for future combined risk assessment. We learned that, from a technical perspective, routine laboratories can accommodate the demands of expanding scopes, as they have successfully incorporated methods to detect emerging mycotoxins into their routine portfolio.
- The study also underscores the demand of certified matrix reference materials for a broad range of mycotoxins, which can be used as internal quality control materials. The availability of such materials is currently restricted, but the study proves that the production of such materials can be stimulated by future proficiency tests especially designed for this purpose.
- The development and production of [^13^C]-labeled standards will become essential for emerging mycotoxins and for the most prevalent compounds, such as BEA, ENNs and MON in particular. The data demonstrated significant benefits for laboratories that applied internal standards for regulated mycotoxins, suggesting this as the most effective way to compensate for matrix effects.

In summary, the outcome of this study proved that a method specific harmonization by means of LC-MS/MS has already been successfully implemented by international operating routine laboratories participating in this study.

## 5. Materials and Methods

### 5.1. Interlaboratory Comparison: Responsibilities and Coordination

The following nine laboratories participated in the ILC study (the order does not match with the individual lab code): Romer Labs Diagnostic GmbH (Analytical Service Department, Tulln, Austria), Romer Labs China Ltd. (Analytical Service Department, Wuxi, China), Romer Labs US Inc (Analytical Service Department, Union, MO, USA), University of Natural Resources and Life Sciences, Vienna (Institute of Bioanalytics and Agro-Metabolomics, Tulln, Austria), Queen’s University Belfast (Institute for Global Food Security, Belfast, Northern Ireland), University of Chemistry and Technology (Department of Food Analysis and Nutrition, Prague, Czech Republic), University of Vienna (Department of Food Chemistry and Toxicology, Vienna, Austria), National Veterinary Research Institute (Department of Pharmacology and Toxicology, Pulawy, Poland), LVA GmbH (Department of Residue Analysis, Klosterneuburg, Austria).

The Interlaboratory comparison study was designed and coordinated by Romer Labs Diagnostic GmbH, including the recruitment of participating laboratories, and the acquisition and distribution of 40 test samples, and the compilation of the data material. All participants are specialized in routine mycotoxin analysis based on mass spectrometric approaches and are either accredited by ISO 17025 or ensure at least an equivalent technical implementation of the standard norm. Samples were collected in June 2021 and distributed to the laboratories in July 2021. Reporting of the results was done in October/November 2021. Data analysis was conducted independently by each participating laboratory. Statistical data analysis was conducted by Romer Labs Diagnostic GmbH. All study participants agreed to the publication of their laboratory and methods identification as well as their measurements data.

### 5.2. Analytes of Interest

Target analytes included in this study are all related to the substance class of secondary fungal metabolites. This includes two groupings of mycotoxins: those with an existing regulatory limit or recommendation such as those mentioned in European Commission Regulation No 1881/2006 and its amendments [15], including aflatoxin B_1_ (AFB1), aflatoxin B_2_ (AFB2), aflatoxin G_1_ (AFG1), aflatoxin G_2_ (AFG2), deoxynivalenol (DON), fumonisin B_1_ (FB1), fumonisin B_2_ (FB2), HT-2 toxin (HT-2), ochratoxin A (OTA), T-2 toxin (T-2) and zearalenone (ZEN); and those known as emerging and masked mycotoxins such as 15-acetyldeoxynivalenol (15-AcDON), 3-acetyldeoxynivalenol (3-AcDON), alternariol (AOH), beauvericin (BEA), deoxynivalenol-3-glucoside (D3G), enniatin A (ENN-A), enniatin A1 (ENN-A1), enniatin B (ENN-B), enniatin B1 (ENN-B1), fumonisin B_3_ (FB3), moniliformin (MON), nivalenol (NIV) and ochratoxin B (OTB), for which an implementation into the current regulatory framework is widely discussed. Table S11 contains an overview of regulated mycotoxins within the European Union. An overview of all compounds analyzed by each participating laboratory in the ILC study is depicted in Table S1. To verify the suitability of the individual calibration standards, two standard mix solutions, including all regulated mycotoxins, were sent to the participants. The participating laboratories measured these standard mixtures together within each sequence of the ILC test to verify the suitability of their calibration standards. Therefore, each laboratory was told to perform a 1:10 dilution of each standard mixture by using their in-house solvent for the external calibration. A threshold of ±20% was set in order to prove the comparability of the participants’ quantification protocol. A detailed description of the standards provided for this study is depicted in Table S12.

### 5.3. Samples

Collection and preparation of the test samples for this interlaboratory comparison was carried out by Romer Labs Diagnostic GmbH. The test materials were prepared from soy, corn gluten, chicken and pig feed retention samples, initially tested and characterized by the in-house Analytical Service Department. In total, 40 test samples at 10 individual lots for each matrix were collected and homogenized thoroughly by using a knife mill (GM 300, Retsch), which ensured a particle size of <300 µm. The test material was tested on the presence of 24 regulated and emerging mycotoxins. No elevation of the natural contamination levels of tested mycotoxins was performed. Each participant received the samples together with an instruction letter. Samples were stored frozen at −20 °C until dispatch.

#### Conduct of Measurements for Homogeneity Study

For the homogeneity assessment, 10 g of each testing lot per matrix was weighed into falcon tubes (*n* = 8) and extracted with 30 mL of extraction solvent acetonitrile:water:formic acid 69.5:29.5:1 (*v*/*v*/*v*). The extracts were shaken for 90 min using a rotary shaker at 200 rpm and centrifuged at 4500 rpm for 4 min. Afterwards, 100 µL of the extract was transferred into a chromatographic vial and diluted with 600 µL of methanol/water/acetic acid 10:89:1 (*v*/*v*/*v*). Analytical measurement was conducted on a Q-Trap 6500+ MS/MS system (SCIEX, Foster city, CA, USA) linked to a 1260 series HPLC system (Agilent Technologies, Waldbronn, Germany). For chromatographic separation a Gemini C18-column, 100 × 4.3 mm i.d. and 3 µm particle size (Phenomenex, Torrance, CA, USA) was used. The autosampler program included an injection volume of 10 µL together with a flow rate of 0.5 mL/min following a binary gradient mode. Mobile phase A was composed of methanol/water/acetic acid 10:89:1 (*v*/*v*/*v*) and mobile phase B of methanol/water/acetic acid 97:2:1 (*v*/*v*/*v*). Both mobile phases contained 5 mM ammonium acetate. Starting conditions of the gradient were 100% A after an initial time of 2 min and the proportion of B was increased linearly to 50% after 5 min. Mobile phase B was increased to 100% after 14 min, followed by a hold time of 4 min. The gradient program was completed after 21 min, including a re-equilibration period of 3 min. Two successive chromatographic runs were performed for each polarity mode following a scheduled reaction monitoring algorithm. This approach was validated for all matrices prior to this study and has achieved an accredited status according to ISO 17025 for all applied analyte–matrix combinations.

**Figure 8:**
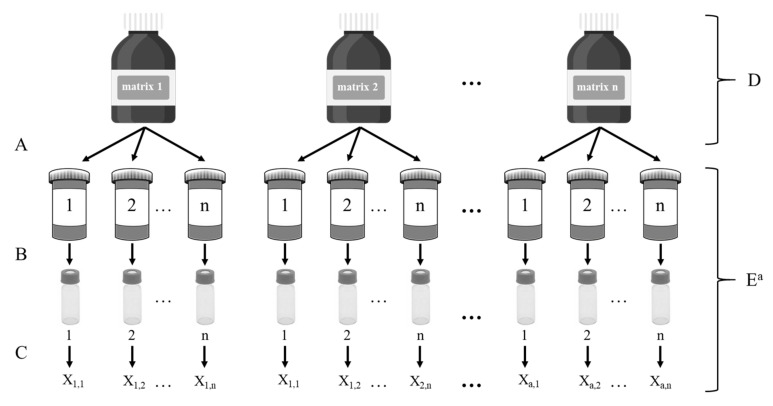
Layout of the between-unit homogeneity study (A = subsampling; B = preparation; C = measurement; D = contributes to the observed between unit variation; E = operations contributing to observed within-unit variation).

Homogeneity testing included both within- and between-unit homogeneity. Between-unit homogeneity is important to ensure that each sample unit carries the same value for each property. The within-unit homogeneity is important if subsamples can be taken for measurement by users of the material [36]. To prove the homogeneity, one randomly selected lot of each test matrix was selected, extracted 8 times and subjected to a fourfold analysis. This resulted in 32 data points for each analyte/lot combination, which were not included in the calculation of the assigned value of the interlaboratory comparison. An overview of the homogeneity study layout is shown in Figure 8**.** Statistical analysis for homogeneity was expressed as maximum between-unit variation (u_bu_) and was calculated according to ISO Guide 35 [36] and Linsinger et al. [37].

Therefore, a one-way ANOVA (analysis of variance) was conducted, taking the mean square sums of the between- and within-unit variances into account. For the final calculation of u_bu_, the between-unit variation (u*_bu_) and the between-unit standard deviation (s_bu_) were calculated. For s_bu_, the between-bottle variance (s^2^_bu_) first had to be calculated with the following equation:

(1) $$s_{\mathrm{bu}}^{2}=\frac{{\mathrm{MS}}_{\mathrm{bu}}-{\mathrm{MS}}_{\mathrm{wu}}}{n_{0}}$$

The MS_bu_ and MS_wu_ represent the so-called between- and within-group mean squares resulting from the ANOVA. If there are no missing data in the study planned to contain n observations per group, n_0_ becomes equal to n. For the calculation of s_bu_ the square root of s^2^_bu_ was taken. The calculation of u*_bu_ included the mean squares within units (MS_wu_) and the degrees of freedom of MS_wu_ (vMS_wu_) and was evaluated based on the following equation:

(2) $$u_{\mathrm{bu}}^{*}=\sqrt{\frac{{\mathrm{Ms}}_{\mathrm{wu}}}{n}}\times \sqrt[{4}]{\frac{2}{{\mathrm{vMS}}_{\mathrm{wu}}}}$$

Finally, for the estimation of the u_bu_, the higher value of (s_bu_ or u*_bu_) was taken as an uncertainty estimate for the homogeneity. The calculation of the contribution of the homogeneity to the overall measurement uncertainty in percent was carried out as follows:

(3) $$u_{\mathrm{bu}}\left(\%\right)=\frac{\left(s_{\mathrm{bu}}{\;\mathrm{or}\;u}_{\mathrm{bu}}^{*}\right)}{{\mathrm{Mean}}_{\mathrm{total}}}\times 100$$

### 5.4. Comparison of Methods for Extraction and Determination

The majority of participating laboratories in this study measured the samples by using an LC-MS/MS configuration (9). One lab additionally measured the entire sample set with a high-resolution detector (Thermo Scientific Q-Exactive Plus). Considering the individual instrumental setup, combinations of Agilent, AB Sciex, Thermo Scientific, Waters and Shimadzu LC-MS/MS systems were applied. All extraction protocols applied by the individual laboratories followed a dilute and shoot approach, including sample volumes between 1 and 10 g. An extraction solution, acidified acetonitrile water mixtures with a volume between 4 and 30 mL were used. No clean-up was included in any of the sample preparation protocols. Chromatographic separation was carried out in reversed phase separation mode under HPLC or UHPLC conditions. As mobile phases, most laboratories used an acidified water solution as mobile phase A and an acidified methanol solution as mobile phase B with ammonium acetate or formate as modifier. The average run time of all methods amounted to 17 min. Matrix effect correction was carried out by five laboratories by injecting a small amount of a [13C]-labelled internal standard mix together with the sample extracts. A summary of the sample preparation protocols as well as instrumental conditions is listed in Table 3. The information provided in this table is based on a random order and does not match the individual laboratory codes. An overview of the lab specific methodology in terms of acquisition parameters, recovery data and limit of quantification is depicted in Tables S13–S22.

### 5.5. Data Analysis

Participants were requested to treat the testing material as a routine sample and to perform the analysis in duplicate (on different days) for each lot, which was then treated as an individual value for the final data analysis. Compound identification criteria followed the recommendations of SANTE/11312/2021 [38] including two product ions per target compound with an ion ratio from sample extracts of ±30% (relative) compared to calibration standards from the same sequence for MS/MS. For HRMS, the criteria included a mass accuracy of ≤5 ppm and analyte peaks from precursor and product ions in the extracted ion chromatograms had to fulfill a complete overlap. Criteria for retention time shifting included an acceptance range of ±0.1 min. For reporting purposes, a template was provided in which the individual mycotoxin level was stated in µg/kg. No corrections of the reported results were conducted by the organizer. The reported results of the compounds listed in Table S1 were subjected to performance assessment and statistical evaluation. The object of the statistical procedure employed was to obtain a simple and transparent result that the participant can readily utilize. The following statistical parameters were calculated based on the submitted data.

#### 5.5.1. Calculation of Assigned Value (X)

The assigned value, X, i.e., the best estimation of the true concentration of the analyte, was set as the consensus of the chromatographical results submitted by participants. The assigned value was calculated as the robust mean by Huber’s H15 method. This approach is also known as “algorithm A” which was originally recommended by the Analytical Methods Committee and provides, in most circumstances, a smaller standard error, as it makes more use of the information in the data compared to the median value [16]. It is, therefore, the method of choice in cases where the distribution pattern is symmetrical [39].

#### 5.5.2. Target Standard Deviation (σ_p_)

The value of σ_p_ determines the limit of satisfactory performance in this interlaboratory comparison study. It is set at a value that reflects best practice for the analyses in question. The standard deviation of reproducibility (RSD_R_) found in collaborative studies is generally considered an appropriate indicator of the best agreement that can be obtained between laboratories. In the case where no appropriate collaborative studies are available, the modified Horwitz equation has proven an appropriate indicator of performance evaluation [40]. The target standard deviation σ_P_ for this ILC was therefore derived from the modified Horwitz equation by using the following function:σp =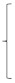
0.22cif c < 1.2 × 10^−7^0.02c ^0.8495^if 1.2 × 10^−7^ ≤ c ≤ 0.1380.01c ^0.5^if c > 0.138

In this function, c represents the mass fraction of the target substance where 0 ≤ c ≤ 1. It further represents a criterion that enables a more realistic calculation of z-scores [18].

#### 5.5.3. z-Scores

The z-score relates the error in the result to the target standard deviation (σ_p_) which is set ahead of the test and reflects “best practice” or fitness for purpose. The z-scores are calculated using the following equation:

(5) $$z=\frac{x-X}{\sigma_{P}}$$

The measurement results reported by the individual participant is represented by x, while X stands for the assigned value that reflects the robust mean. The standard deviation σ_p_ for the interlaboratory comparison study is derived from the modified Horwitz equation as described in Section 5.5.2. A z-score >2 is usually taken as an indication that the investigation of possible causes is necessary; a z-score >3 is commonly used as an intervention signal that indicates the need for corrective actions [18]. Therefore, the following interpretation was conducted:
|z| ≤ 2result is acceptable2 < |z| ≤ 3result is questionable|z| > 3result is unacceptable

## Figures and Tables

**Table 1:** Compilation of homogeneity study results for samples of chicken and swine feed, soy and corn gluten testing positive for mycotoxins. All analytes passed the homogeneity criteria in terms of s_bu_ ≤ 0.3 σ_p_ and u_bu_ ≤ 10%.

Matrix	Compound	Average [µg/kg]	s_wu_ [µg/kg]	s_bu_ [µg/kg]	σ_p_ [µg/kg]	u_bu_ [%]
Chicken feed	15-acetyldeoxynivalenol	114	6.32	4.04	25.0	4
	alternariol	25.3	1.11	1.19	5.57	5
	beauvericin	4.46	1.37	0.00	0.98	8
	deoxynivalenol-3-glucoside	78.2	3.45	4.74	17.2	6
	deoxynivalenol	413	37.0	0.00	75.5	2
	enniatin B	15.3	2.00	0.00	3.37	4
	enniatin B1	10.7	1.93	0.00	2.36	5
	fumonisin B_1_	182	5.74	8.20	37.6	5
	fumonisin B_2_	45.9	3.19	0.00	10.1	2
	fumonisin B_3_	15.8	1.50	0.37	3.47	3
	HT-2 toxin	53.8	20.3	1.28	11.8	10
	moniliformin	38.9	0.94	2.23	8.56	6
	ochratoxin A	0.38	0.10	0.02	0.08	7
	T-2 toxin	39.8	2.86	0.35	8.76	2
	zearalenone	45.0	3.06	1.60	9.89	4
Swine feed	enniatin B	2.77	0.62	0.00	0.61	6
	fumonisin B_1_	163	7.79	8.05	34.2	5
	fumonisin B_2_	36.6	2.02	0.90	8.04	2
	fumonisin B_3_	12.4	1.27	0.72	2.73	6
	moniliformin	53.2	2.37	2.40	11.7	5
	ochratoxin A	16.6	0.72	0.92	3.65	6
	T-2 toxin	5.93	0.65	0.22	1.30	4
	zearalenone	4.65	0.16	0.29	1.02	6
Soy	enniatin A	0.95	0.02	0.00	0.21	0.4
	enniatin B	1.46	0.09	0.07	0.32	5
	enniatin B1	1.40	0.05	0.02	0.31	2
	fumonisin B_1_	5.41	0.66	0.00	1.19	3
	fumonisin B_2_	4.94	0.52	0.23	1.09	5
	zearalenone	1.63	0.12	0.04	0.36	2
Corn Gluten	15-acetyldeoxynivalenol	203	9.42	5.67	41.3	3
	alternariol	19.7	0.92	0.70	4.34	4
	beauvericin	56.2	3.40	2.64	12.4	5
	deoxynivalenol	311	12.0	0.00	59.3	1
	enniatin A	0.33	0.05	0.01	0.07	4
	enniatin A1	2.01	0.37	0.00	0.44	5
	enniatin B	13.3	0.56	0.71	2.92	5
	enniatin B1	7.50	0.40	0.36	1.65	5
	fumonisin B_1_	1041	58.5	14.4	166	2
	fumonisin B_2_	552	16.9	0.00	96.5	1
	fumonisin B_3_	174	7.35	2.95	36.2	2
	HT-2 toxin	75.7	19.3	0.00	16.7	7
	moniliformin	8.03	0.47	0.00	1.77	2
	ochratoxin A	2.13	0.22	0.00	0.47	3
	T-2 toxin	35.5	3.54	0.00	7.81	3
	zearalenone	620	20.1	15.8	107	3

s_wu_ = within unit standard deviation; s_bu_ = between unit standard deviation; σ_p_ = standard deviation for interlaboratory comparison study using modified Horwitz equation; s_bu_ ≤ 0.3 σ_p_ = homogeneity check based on ISO 13528; u_bu_ ≤ 10% = homogeneity check based on the maximum between unit variation.

**Table 2:** Summary of statistical data for 11 regulated and 13 non-regulated mycotoxins in 10 individual chicken feed, swine feed, corn gluten and soy samples.

	15-Ac-DON	3-Ac-DON	AFB1	AFB2	AFG1	AFG2	AOH	BEA	D3G	DON	ENN-A	ENN-A1	ENN-B	ENN-B1	FB1	FB2	FB3	HT-2	MON	NIV	OTA	OTB	T-2	ZEN
No. of participants	6	6	10	10	10	10	7	7	5	10	7	7	5	7	10	10	5	10	5	7	10	7	10	10
**Chicken Feed**																								
No. of quantitative results	66	26	43	1	10	1	89	160	47	157	54	99	140	153	170	146	29	62	65	14	88	19	114	187
No. of statistical data points	60	6	7	-	-	-	89	160	39	157	30	99	140	153	164	146	19	42	61	-	76	14	110	187
Max assigned value (µg/kg)	81.1	19.6	1.51	-	-	-	16.2	41.2	80.3	725	0.83	5.09	91.3	21.3	340	97.3	40.1	34.7	116	-	4.71	3.51	25.6	66.3
Med assigned value (µg/kg)	42.6	19.6	1.51	-	-	-	3.97	15.4	68.1	250	0.58	2.23	18.5	6.21	127	44.7	26.0	15.6	63.3	-	2.06	3.51	4.53	29.0
Min assigned value (µg/kg)	28.7	19.6	1.51	-	-	-	1.11	7.32	54.3	17.8	0.40	1.37	4.79	3.54	29.2	15.4	15.9	9.71	11.6	-	0.50	3.51	1.69	7.01
Acceptable z-scores in %	87	33	57	-	-	-	47	61	69	69	93	61	61	66	65	54	95	76	95	-	47	50	76	83
Questionable z-scores in %	8	-	-	-	-	-	21	21	15	18	7	14	16	17	16	16	5	17	5	-	17	-	11	11
Unacceptable z-scores in %	5	67	43	-	-	-	31	19	15	13	-	25	22	17	19	30	-	7	-	-	36	50	13	6
**Swine Feed**																								
No. of quantitative results	72	44	21	2	10	4	81	150	73	179	78	121	151	142	124	100	20	50	80	45	72	9	72	159
No. of statistical data points	62	30	15	-	10	-	73	150	59	179	74	121	151	142	114	89	12	38	80	41	60	-	60	159
Max assigned value (µg/kg)	100	30.6	2.67	-	0.83	-	19.1	25.3	156	1185	3.51	17.1	77.0	51.7	673	174	83.1	14.0	120	85.3	16.7	-	5.32	69.0
Med assigned value (µg/kg)	44.9	26.7	2.67	-	0.83	-	4.11	7.04	125	365	2.60	9.21	47.5	24.0	76.5	13.4	83.1	9.69	43.3	51.9	5.81	-	3.00	13.2
Min assigned value (µg/kg)	18.8	9.43	2.67	-	0.83	-	1.30	2.47	62.2	36.5	0.67	0.69	3.94	2.17	22.6	7.40	83.1	3.66	13.4	25.6	1.97	-	1.43	3.14
Acceptable z-scores in %	55	57	93	-	90	-	58	75	90	72	85	74	67	72	71	63	100	82	69	76	62	-	70	81
Questionable z-scores in %	15	23	7	-	0	-	18	12	7	12	8	14	18	15	11	9	-	8	21	24	15	-	18	9
Unacceptable z-scores in %	31	20	-	-	10	-	25	13	3	16	7	12	15	13	18	28	-	11	10	-	23	-	12	10
	**15-Ac-DON**	**3-Ac-DON**	**AFB1**	**AFB2**	**AFG1**	**AFG2**	**AOH**	**BEA**	**D3G**	**DON**	**ENN-A**	**ENN-A1**	**ENN-B**	**ENN-B1**	**FB1**	**FB2**	**FB3**	**HT-2**	**MON**	**NIV**	**OTA**	**OTB**	**T-2**	**ZEN**
No. of participants	6	6	10	10	10	10	7	7	5	10	7	7	5	7	10	10	5	10	5	7	10	7	10	10
**Corn Gluten**																								
No. of quantitative results	77	39	37	2	18	-	127	140	14	138	36	70	117	103	198	192	113	141	65	4	95	8	163	184
No. of statistical data points	73	24	26	-	6	-	127	140	8	138	14	68	113	95	198	192	113	139	65	-	80	-	160	184
Max assigned value (µg/kg)	257	81.2	1.89	-	1.74	-	22.0	445	121	837	0.72	4.98	20.1	12.3	1481	706	286	61.7	193	-	8.93	-	43.1	825
Med assigned value (µg/kg)	124	58.9	1.82	-	1.74	-	15.7	287	121	216	0.69	1.39	9.51	3.31	787	391	137	47.1	12.5	-	6.47	-	34.8	135
Min assigned value (µg/kg)	72.3	52.7	0.85	-	1.74	-	1.77	23.3	121	98.9	0.65	1.28	3.24	2.47	315	88.7	30.6	13.3	5.10	-	4.29	-	5.68	2.86
Acceptable z-scores in %	52	58	88	-	50	-	68	62	63	55	100	69	59	85	69	74	85	67	74	-	70	-	88	79
Questionable z-scores in %	29	13	8	-	17	-	20	7	38	17	-	19	15	15	9	17	13	17	18	-	18	-	5	11
Unacceptable z-scores in %	19	29	4	-	33	-	12	31	-	28	-	12	26	-	23	9	2	16	8	-	13	-	8	10
**Soy**																								
No. of quantitative results	11	17	23	6	8	-	40	106	-	39	22	45	93	85	27	42	-	63	12	-	36	6	52	99
No. of statistical data points	-	-	23	-	8	-	27	106	-	7	20	34	92	85	-	-	-	35	6	-	16	-	33	85
Max assigned value (µg/kg)	-	-	2.60	-	1.18	-	27.1	23.3	-	36.4	5.78	17.8	236	65.1	-	-	-	107	4.45	-	1.54	-	20.3	366
Med assigned value (µg/kg)	-	-	1.79	-	1.18	-	16.7	2.50	-	36.4	3.13	2.06	2.54	0.88	-	-	-	82.5	4.45	-	1.50	-	11.7	3.23
Min assigned value (µg/kg)	-	-	0.97	-	1.18	-	6.35	0.89	-	36.4	0.49	0.66	0.66	0.32	-	-	-	57.8	4.45	-	1.45	-	3.16	1.42
Acceptable z-scores in %	-	-	70	-	50	-	74	63	-	57	75	88	72	84	-	-	-	83	33	-	25	-	82	76
Questionable z-scores in %	-	-	13	-	50	-	7	10	-	-	20	3	14	5	-	-	-	6	33	-	50	-	-	11
Unacceptable z-scores in %	-	-	17	-	-	-	19	26	-	43	5	9	14	12	-	-	-	11	33	-	25	-	18	13

one laboratory delivered two additional data sets.

**Table 3:** Method description summary including information regarding the sample preparation and instrumental conditions (randomly listed).

HPLC System	Detection System	Weight (g)	Extraction Solvent	Volume (mL)	Chromatographic Column	Mobile Phase A	Mobile Phase B	Run Time (min)	Quant
Thermo Scientific UltiMate 3000	Thermo Scientific TSQ Vantage	5	79:20:1 ACN:H_2_O:HAc	20	Waters Acquity UPLC HSS T3 1.8 µm, 2.1 × 100 mm	H_2_O (0.1% HAc 5 mM CH_3_COONH_4_)	MeOH	19.0	ENS
Agilent 1290 series	Agilent 6470	5	79:20.9:0.1 ACN:H_2_O:HFo	20	RRHD-Zorbax Eclipse Plus C18 1.8 µm 2.1 × 100 mm	H_2_O (0.1% HAc 5 mM NH_4_OOCH)	MeOH (0.1% HAc 5 mM NH_4_OOCH)	11.5	ENS + ISTD
Agilent 1290 series	AB Sciex QTrap 5500	5	79:20:1 ACN:H_2_O:HAc	20	Phenomenex Gemini C18 5 µm, 4.6 × 150 mm	89:10:1 H_2_O:MeOH:HAc (5 mM CH_3_COONH_4_)	2:97:1 H_2_O:MeOH:HAc (5 mM CH_3_COONH_4_)	21.5	ENS
AB Sciex ExionLC AD	AB Sciex QTrap 5500	1	79:20:1 ACN:H_2_O:HAc	4	Phenomenex Gemini C18 5 µm, 4.6 × 100 mm	89:10:1 H_2_O:MeOH:HAc (5 mM CH_3_COONH_4_)	2:97:1 H_2_O:MeOH:HAc (5 mM CH_3_COONH_4_)	13.5	ENS
Waters Acquity	AB Sciex QTrap 5500	2	50:50 ACN:H_2_O (0.2% HFo)	20	Waters Acquity UPLC HSS T3 1.8 µm, 2.1 × 100 mm	H_2_O (0.2% HFo 5 mM NH_4_OOCH)	MeOH (0.2% HFo 5 mM NH_4_OOCH)	12.0	ENS
Shimadzu	AB Sciex 5500+	5	79:20:1 ACN:H_2_O:HAc	20	Phenomenex Gemini C18 5 µm, 4.6 × 150 mm	89:10:1 H_2_O:MeOH:HAc (5 mM CH_3_COONH_4_)	2:97:1 H_2_O:MeOH:HAc (5 mM CH_3_COONH_4_)	20.6	ENS + ISTD
Thermo Scientific UltiMate 3000	Thermo Scientific Q-Exactive Plus	2	50:50 ACN:H_2_O (0.2% HFo)	20	Waters Acquity UPLC HSS T3 1.8 µm, 2.1 × 100 mm	H_2_O (0.2% HFo 5 mM NH_4_OOCH)	MeOH (0.2% HFo 5 mM NH_4_OOCH)	12.0	MMC
Agilent 1290 series	AB Sciex QTrap 5500	10	69.5:29.5:1 ACN:H_2_O:HFo	30	Phenomenex Gemini C18 3 µm, 4.3 × 100 mm	89:10:1 H_2_O:MeOH:HAc (5 mM CH_3_COONH_4_)	2:97:1 H_2_O:MeOH:HAc (5 mM CH_3_COONH_4_)	19.5	ENS + ISTD
Agilent 1260 series	AB Sciex QTrap 6500+	10	69.5:29.5:1 ACN:H_2_O:HFo	30	Phenomenex Gemini C18 3 µm, 4.3 × 100 mm	89:10:1 H_2_O:MeOH:HAc (5 mM CH3COONH_4_)	2:97:1 H_2_O:MeOH:HAc (5 mM CH_3_COONH_4_)	19.5	ENS + ISTD
Shimadzu Nexera X2	Shimadzu 8050	1	79:20:1 ACN:H_2_O:Hfo	4	Phenomenex Kinetex BiPhenyl 2.1 µm, 100 × 2.1 mm	95:5 H_2_O:MeOH (0.1% HAc 0.01 M CH_3_COONH_4_)	5:95 H_2_O:MeOH (0.1% HAc 0.01 M CH_3_COONH_4_)	16.0	MMC + ISTD

HAc = acetic acid; HFo = formic acid; Quant = how was the quantification carried out; MMC = matrix matched calibration; ISTD = internal standard; ENS = external neat solvent calibration.

## Data Availability

Not applicable.

## Supplementary Materials

The following supporting information can be downloaded at: https://www.mdpi.com/article/10.3390/toxins14060405/s1, Figure S1: Graphical illustration of H15-mean based concentration ranges in soy matrix. The x-axis represents the concentration range for the specific assigned values in µg/kg in a logarithmic scale. The y-axis shows the individual target compounds; Figure S2: Graphical illustration of H15-mean based concentration ranges in corn gluten matrix. The x-axis represents the concentration range for the specific assigned values in µg/kg in a logarithmic scale. The y-axis shows the individual target compounds; Figure S3: Graphical illustration of H15-mean based concentration ranges in chicken feed matrix. The x-axis represents the concentration range for the specific assigned values in µg/kg in a logarithmic scale. The y-axis shows the individual target compounds; Figure S4: Graphical illustration of H15-mean based concentration ranges in swine feed matrix. The x-axis represents the concentration range for the specific assigned values in µg/kg in a logarithmic scale. The y-axis shows the individual target compounds; Table S1: Overview of the target compound list for the interlaboratory comparison study. The columns include the name of the compound (analyte), the typical abbreviation (abbr.) and an indication of which compounds have been included in the testing scheme of the individual participant; Table S2: Analyte specific H15-mean based concentration range for 10 individual soy matrix lots; Table S3: Analyte specific H15-mean based concentration range for 10 individual corn gluten matrix lots; Table S4: Analyte-specific H15-mean based concentration range for 10 individual chicken feed matrix lots; Table S5: Analyte-specific H15-mean based concentration range for 10 individual swine feed matrix lots; Table S6: Summary of z-score performance of 10 soy matrices. Acceptable, questionable and unacceptable z-scores are colored in green, yellow and red respectively. No-stat information refers to positive findings where a z-score calculation was not feasible due to a reduced number of reported results; Table S7: Summary of z-score performance of 10 corn gluten matrices. Acceptable, questionable and unacceptable z-scores are colored in green, yellow and red, respectively. No-stat information refers to positive findings where a z-score calculation was not feasible due to a reduced number of reported results; Table S8: Summary of z-score performance of 10 chicken feed matrices. Acceptable, questionable and unacceptable z-scores are colored in green, yellow and red, respectively. No-stat information refers to positive findings where a z-score calculation was not feasible due to a reduced number of reported results; Table S9: Summary of z-score performance of 10 swine feed matrices. Acceptable, questionable and unacceptable z-scores are colored in green, yellow and red, respectively. No-stat information refers to positive findings, where a z-score calculation was not feasible due to a reduced number of reported results; Table S10: Overview of analyte specific z-score deviations from ± 2 per laboratory and matrix; Table S11: Overview of EU regulated mycotoxins; Table S12: Preparation scheme of the control solutions provided by the study organizer. The participants were informed to dilute the standard mixtures (conc. mix) of each vial in a ratio of 1:10 by using the same solvent as for their calibration standards; Table S13: Lab 001 method information; Table S14: Lab 002 method information; Table S15: Lab 003 method information; Table S16: Lab 004 method information; Table S17: Lab 005 method information; Table S18: Lab 006 method information; Table S19: Lab 007 method information; Table S20: Lab 008 method information; Table S21: Lab 009 method information; Table S22: Lab 010 method information.

## References

[1] Eskola M., Elliott C.T., Hajšlová J., Steiner D., Krska R. (2020). Towards a dietary-exposome assessment of chemicals in food: An update on the chronic health risks for the European consumer. Crit. Rev. Food Sci. Nutr..

[2] Eskola M., Kos G., Elliott C.T., Hajšlová J., Mayar S., Krska R. (2020). Worldwide contamination of food-crops with mycotoxins: Validity of the widely cited ‘FAO estimate’ of 25%. Crit. Rev. Food Sci. Nutr..

[3] De Girolamo A., Ciasca B., Stroka J., Bratinova S., Pascale M., Visconti A., Lattanzio V.M.T. (2017). Performance evaluation of LC–MS/MS methods for multi-mycotoxin determination in maize and wheat by means of international Proficiency Testing. TrAC Trends Anal. Chem..

[4] Malachová A., Sulyok M., Beltrán E., Berthiller F., Krska R. (2014). Optimization and validation of a quantitative liquid chromatography-tandem mass spectrometric method covering 295 bacterial and fungal metabolites including all regulated mycotoxins in four model food matrices. J. Chromatogr. A.

[5] Krska R., Schubert-Ullrich P., Molinelli A., Sulyok M., Macdonald S., Crews C. (2008). Mycotoxin analysis: An update. Food Addit. Contam. Part A Chem. Anal. Control Expo. Risk Assess..

[6] FAO (2020). Climate Change: Unpacking the Burden on Food Safety.

[7] Steiner D., Malachová A., Sulyok M., Krska R. (2021). Challenges and future directions in LC-MS-based multiclass method development for the quantification of food contaminants. Anal. Bioanal. Chem..

[8] Steiner D., Sulyok M., Malachová A., Mueller A., Krska R. (2020). Realizing the simultaneous liquid chromatography-tandem mass spectrometry based quantification of >1200 biotoxins, pesticides and veterinary drugs in complex feed. J. Chromatogr. A.

[9] Tittlemier S.A., Cramer B., Dall’Asta C., DeRosa M.C., Lattanzio V.M.T., Malone R., Maragos C., Stranska M., Sumarah M. (2022). Developments in mycotoxin analysis: An update for 2020–2021. World Mycotoxin J..

[10] Steiner D., Krska R., Malachová A., Taschl I., Sulyok M. (2020). Evaluation of Matrix Effects and Extraction Efficiencies of LC-MS/MS Methods as the Essential Part for Proper Validation of Multiclass Contaminants in Complex Feed. J. Agric. Food Chem..

[11] Sulyok M., Stadler D., Steiner D., Krska R. (2020). Validation of an LC-MS/MS-based dilute-and-shoot approach for the quantification of >500 mycotoxins and other secondary metabolites in food crops: Challenges and solutions. Anal. Bioanal. Chem..

[12] (2005). General Requirements for the Competence of Testing and Calibration Laboratories.

[13] Sibanda L., McCallum K., Plotan M., Webb S., Snodgras B., Muenks Q., Porter J., Fitzgerald P. (2022). Interlaboratory collaboration to determine the performance of the Randox food diagnostics biochip array technology for the simultaneous quantitative detection of seven mycotoxins in feed. World Mycotoxin J..

[14] (2015). Statistical methods for use in proficiency testing by interlaboratory comparison.

[15] Commission of the European Union (2006). COMMISSION REGULATION (EC) No 1881/2006. Off. J. Eur. Union.

[16] Thompson M., Ellison S.L.R., Wood R. (2006). The International Harmonized Protocol for the proficiency testing of analytical chemistry laboratories (IUPAC Technical Report). Pure Appl. Chem..

[17] Asuero A.G., Sayago A., Gonzalez A.G. (2006). The correlation coefficient: An overview. Crit. Rev. Anal. Chem..

[18] Häggblom P., Nordkvist E. (2015). Deoxynivalenol, zearalenone, and Fusarium graminearum contamination of cereal straw; field distribution; and sampling of big bales. Mycotoxin Res..

[19] Rheeder J.P., Marasas W.F.O., Vismer H.F. (2002). Production of fumonisin analogs by Fusarium species. Appl. Environ. Microbiol..

[20] Jestoi M. (2008). Emerging fusarium-mycotoxins fusaproliferin, beauvericin, enniatins, and moniliformin—A review. Crit. Rev. Food Sci. Nutr..

[21] Ropejko K., Twarużek M. (2021). Zearalenone and Its Metabolites-General Overview, Occurrence, and Toxicity. Toxins.

[22] Streit E., Schwab C., Sulyok M., Naehrer K., Krska R., Schatzmayr G. (2013). Multi-mycotoxin screening reveals the occurrence of 139 different secondary metabolites in feed and feed ingredients. Toxins.

[23] Gruber-Dorninger C., Novak B., Nagl V., Berthiller F. (2017). Emerging Mycotoxins: Beyond Traditionally Determined Food Contaminants. J. Agric. Food Chem..

[24] Martínez-Domínguez G., Romero-González R., Arrebola F.J., Garrido Frenich A. (2016). Multi-class determination of pesticides and mycotoxins in isoflavones supplements obtained from soy by liquid chromatography coupled to Orbitrap high resolution mass spectrometry. Food Control.

[25] Habinshuti I., Chen X., Yu J., Mukeshimana O., Duhoranimana E., Karangwa E., Muhoza B., Zhang M., Xia S., Zhang X. (2019). Antimicrobial, antioxidant and sensory properties of Maillard reaction products (MRPs) derived from sunflower, soybean and corn meal hydrolysates. LWT.

[26] Patil U.S., King S., Holleran S., White K., Stephenson C., Reuther J. (2019). Identifying challenges and risks associated with the analysis of major mycotoxins in feed and botanicals. J. AOAC Int..

[27] Rao Z.-X., Tokach M.D., Woodworth J.C., DeRouchey J.M., Goodband R.D., Calderón H.I., Dritz S.S. (2020). Effects of fumonisin-contaminated corn on growth performance of 9 to 28 kg nursery pigs. Toxins.

[28] Kovalsky P., Kos G., Nährer K., Schwab C., Jenkins T., Schatzmayr G., Sulyok M., Krska R. (2016). Co-occurrence of regulated, masked and emerging mycotoxins and secondary metabolites in finished feed and maize–An extensive survey. Toxins.

[29] Vohra M., Manwar J., Manmode R., Padgilwar S., Patil S. (2014). Bioethanol production: Feedstock and current technologies. J. Environ. Chem. Eng..

[30] Saunders D.S., Meredith F.I., Voss K.A. (2001). Control of Fumonisin: Effects of Processing. Environ. Health Perspect..

[31] Prettl Z.S., Lepossa A., Tóth É., Kelemen-Horváth I., Németh Á.S., Nagy E. (2011). Effects and changes of zearalenone and fumonisin contamination in corn-based bioethanol process. Hung. J. Ind. Chem..

[32] Berwanger E., Nunes R.V., Pasquetti T.J., Murakami A.E., De Oliveira T.M.M., Bayerle D.F., Frank R. (2017). Sunflower cake with or without enzymatic complex for broiler chickens feeding. Asian-Australasian J. Anim. Sci..

[33] Varga E., Glauner T., Köppen R., Mayer K., Sulyok M., Schuhmacher R., Krska R., Berthiller F. (2012). Stable isotope dilution assay for the accurate determination of mycotoxins in maize by UHPLC-MS/MS. Anal. Bioanal. Chem..

[34] Wang J., Cheung W. (2006). Determination of pesticides in soy-based infant formula using liquid chromatography with electrospray ionization tandem mass spectrometry. J. AOAC Int..

[35] Meneely J.P., Ricci F., Van Egmond H.P., Elliott C.T. (2011). Current methods of analysis for the determination of trichothecene mycotoxins in food. TrAC Trends Anal. Chem..

[36] (2017). Guide 35—Reference Materials—Guidance for Characterization and Assessment of Homogeneity and Stability.

[37] Linsinger T.P.J., Pauwels J., Van Der Veen A.M.H., Schimmel H., Lamberty A. (2001). Homogeneity and stability of reference materials. Accredit. Qual. Assur..

[38] (2021). Analytical Quality Control and Method Validation for Pesticide Residues Analysis in Food and Feed.

[39] Bao L., Bao Z., Zhang Y., Liang C., Lu N., Liu X., Jin Y., Pei J.Z., Ge M., Tian L. (2009). Aflatoxin Testing in Peanuts: A Proficiency Assessment Scheme for Chinese Analytic Laboratories. Food Chem. Contam..

[40] Thompson M. (2000). Recent trends in inter-laboratory precision at ppb and sub-ppb concentrations in relation to fitness for purpose criteria in proficiency testing. Anal. Commun..

