# Supplementary material for: An Interlaboratory Comparison Study of Regulated and Emerging Mycotoxins Using Liquid Chromatography Mass Spectrometry: Challenges and Future Directions of Routine Multi-Mycotoxin Analysis including Emerging Mycotoxins
Source: Toxins (Basel). 2022 Jun 13;14(6):405. doi: 10.3390/toxins14060405 (PMC9229327; doi:10.3390/toxins14060405)
Supplement: Supplementary file 1 [file toxins-14-00405-s001.zip › toxins-1743744-supplementary.pdf]

Supplementary Material: An Interlaboratory Comparison Study of Regulated and Emerging Mycotoxins Using Liquid Chromatography Mass Spectrometry: Challenges and Future Directions of Routine Multi-Mycotoxin Analysis Including Emerging Mycotoxins

David Steiner, Armin Humpel, Eleonore Stamminger, Anna Schoeberl, Gerlinde Pachschwoell, Anita Sloboda, Christy Swoboda, Jolene Rigg, Dawei Zhang, Yahong Wang, Joshua Davis, Michael Sulyok, Rudolf Krska, Brian Quinn, Brett Greer, Christopher T. Elliott, Zbynek Dzuman, Jana Hajslova, Andreas Gschaider, Carina Fechner, Lisa Forstner, Elisabeth Varga, Piotr Jedziniak, Katarzyna Pietruszka, Adrianna Rudawska and Alexandra Malachová

Table of Contents

1. Overview of tested analytes 3

1.1. Summary of positive findings 3

2. Matrix specific concentration ranges 5

2.1. Concentration range in soy matrices 5

2.2. Concentration range in corn gluten matrices 6

2.3. Concentration range in chicken feed matrices 7

2.4. Concentration range in swine feed matrices 8

3. Analyte specific z-score performance 9

3.1. Overview of individual z-score data for soy matrix 10

3.2. Overview of individual z-score data for corn gluten matrix 15

3.3. Overview of individual z-score data for chicken feed matrix 20

3.4. Overview of individual z-score data for swine feed matrix 25

4. Overview of z-score deviations 30

5. Regulatory framework of the European Union 31

6. Control Standard Solutions 32

7. Lab specific methodology 33

1. Overview of tested analytes

Table S1: Overview of the target compound list for the interlaboratory comparison study. The columns include the name of the compound (analyte), the typical abbreviation (abbr.) and an indication which compounds has been included into the testing scheme of the individual participant.

ANALYTE	ABBR.	LAB 001	LAB 002	LAB 003	LAB 004	LAB 005	LAB 006	LAB 007	LAB 008	LAB 009*
		compound tested								
Aflatoxin B1	AFB1	yes	yes	yes	yes	yes	yes	yes	yes	yes
Aflatoxin B2	AFB2	yes	yes	yes	yes	yes	yes	yes	yes	yes
Aflatoxin G1	AFG1	yes	yes	yes	yes	yes	yes	yes	yes	yes
Aflatoxin G2	AFG2	yes	yes	yes	yes	yes	yes	yes	yes	yes
Deoxynivalenol	DON	yes	yes	yes	yes	yes	yes	yes	yes	yes
Fumonisin B1	FB1	yes	yes	yes	yes	yes	yes	yes	yes	yes
Fumonisin B2	FB2	yes	yes	yes	yes	yes	yes	yes	yes	yes
HT-2 Toxin	HT-2	yes	yes	yes	yes	yes	yes	yes	yes	yes
Ochratoxin A	OTA	yes	yes	yes	yes	yes	yes	yes	yes	yes
T-2 Toxin	T-2	yes	yes	yes	yes	yes	yes	yes	yes	yes
Zearalenone	ZEN	yes	yes	yes	yes	yes	yes	yes	yes	yes
15-Acetyldeoxynivalenol	15-AcDON	yes	yes	no	yes	yes	no	no	yes	yes
3-Acetyldeoxynivalenol	3-AcDON	yes	yes	no	yes	yes	no	no	yes	yes
Alternariol	AOH	yes	yes	no	yes	yes	yes	no	yes	yes
Beauvericin	BEA	yes	yes	no	yes	yes	yes	no	yes	yes
Deoxynivalenol-3-Glucoside	D3G	yes	yes	no	yes	yes	no	no	no	yes
Enniatin A	ENN-A	yes	yes	no	yes	yes	yes	no	yes	yes
Enniatin A1	ENN-A1	yes	yes	no	yes	yes	yes	no	yes	yes
Enniatin B	ENN-B	no	yes	no	no	yes	yes	no	yes	yes
Enniatin B1	ENN-B1	yes	yes	no	yes	yes	yes	no	yes	yes
Fumonisin B3	FB3	yes	yes	no	yes	yes	no	no	no	yes
Moniliformin	MON	yes	yes	no	yes	yes	no	no	yes	no
Nivalenol	NIV	yes	yes	no	yes	yes	yes	no	yes	yes
Ochratoxin B	OTB	yes	yes	no	yes	yes	yes	no	yes	yes

*Lab 009 provided an additional data set which is listed as lab 010

Five laboratories tested for 62 – 92% (19 – 23) of all compounds and only two laboratories did not include any non-regulated mycotoxins in their testing scope. Furthermore, 4 participants provided results for 46 – 100% of analytes which complied with the accreditation requirements of ISO 17025. Based on a low number of quantitative results, a statistical analysis for AFB2, AFG2 in all 4 matrix commodities as well as AFG1 in chicken feed, OTB in swine feed, corn gluten and soy, NIV in chicken feed, corn gluten and soy and 15-AcDON, 3-AcDON, D3G, FB1, FB2 and FB3 in soy was not feasible. Some participants reported results lower their individual reporting and detection limits, which was especially true for aflatoxins and ochratoxin A. However, these results were excluded for the final statistical calculation.

1.1. Summary of positive findings

In total, 6712 quantitative results were reported for all analyte/matrix combinations. For statistical calculation 6018 results (89.7%) were used, whereby 3075 results were related to the group of non- regulated and 2943 to the group of regulated mycotoxins.

The highest positive rate of quantitative results (> LOQ) for all analytes was observed in corn gluten samples with 2081 positive findings out of 4800 possible events which results in a 43.4% overall contamination rate. This is followed by chicken feed with 1940 (39.6%), swine feed with 1859 (38.7%) and soy with 832 (17.3%) total positive findings. Amongst the regulated mycotoxins, ZEN, FB1 and DON were the most prevalent representatives with 629 (78.6%), 519 (69.5%) and 513 (64.9%) reported quantitative results. The fewest positive findings were observed for aflatoxins with 124 (15.5%) reported results for AFB1, 46 (5.8%)

for AFG1, 11 (1.4%) for AFB2 and 5 (0.6%) for AFG2. Quantitative results for the remaining regulated mycotoxins accounted 480 (60.0%) for FB2, 401 (50.1%) for T-2 toxin, 316 (39.5%) for HT-2 toxin, 291 (36.4%) for OTA and 162 (20.3%) for FB3.

The highest contamination rate for non-regulated mycotoxins was observed for BEA with 556 (69.5%), ENN-B with 501 (62.6%) and ENN-B1 with 483 (60.4%) quantitative reported results. Moderate findings were made for AOH with 337 (42.1%), ENN-A1 with 335 (41.9%), 15-AcDON with 226 (28.3%), MON with 222 (27.8%) and ENN-A with 190 (23.8%) reported quantitative data. The lowest quantitative dataset was reported for D3G with 134 (16.8%), 3-AcDON with 126 (15.8%), NIV with 63 (7.9%) and OTB with 42 (5.3%) of positive results.

2. Matrix specific concentration ranges

2.1. Concentration range in soy matrices

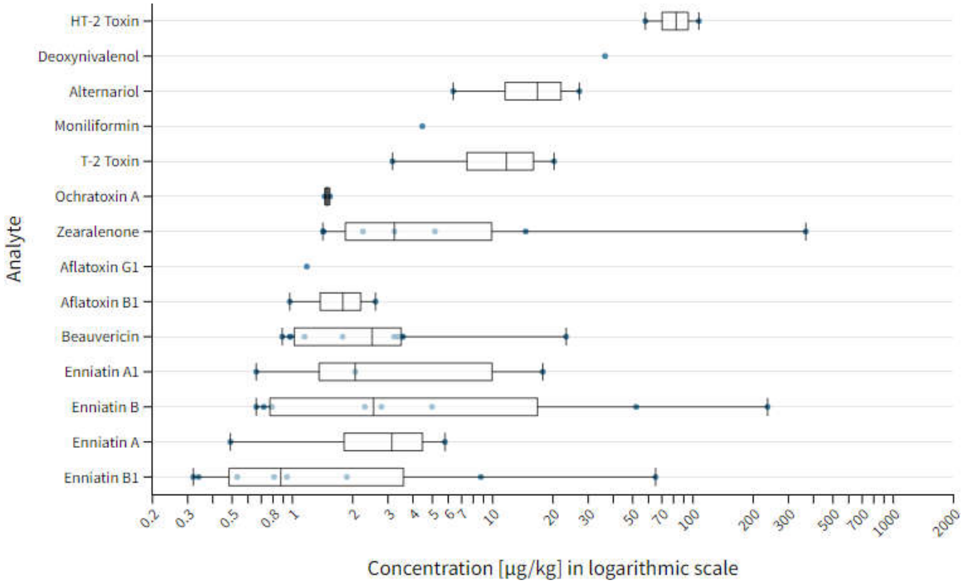

Figure S1: Graphical illustration of H15-mean based concentration ranges in soy matrix. The x-axis represents the concentration range for the specific assigned values in µg/kg in a logarithmic scale. The y-axis shows theindividual target compounds.

Table S2: Analyte specific H15-mean based concentration range for 10 individual soy matrix lots.

Lot	15-AcDON	3-AcDON	AFB1	AFB2	AFG1	AFG2	AOH	BEA	D3G	DON	ENN-A	ENN-A1	ENN-B	ENN-B1	FB1	FB2	FB3	HT-2	MON	NIV	OTA	OTB	T-2	ZEN
1	-	-	-	-	-	-	-	3.56	-	-	-	-	0.79	0.53	-	-	-	-	-	-	-	-	-	-
2	-	-	2.60	-	-	-	-	0.89	-	-	-	-	-	-	-	-	-	-	-	-	1.54	-	-	-
3	-	-	-	-	-	-	6.35	3.37	-	-	0.49	2.06	52.0	8.73	-	-	-	57.8	-	-	-	-	3.16	14.6
4	-	-	-	-	-	-	-	0.97	-	-	-	-	-	-	-	-	-	-	-	-	-	-	-	-
5	-	-	-	-	-	-	-	3.22	-	-	-	-	0.66	0.34	-	-	-	-	4.45	-	-	-	-	3.23
6	-	-	-	-	-	-	-	0.98	-	-	-	-	2.78	0.94	-	-	-	-	-	-	1.45	-	-	1.43
7	-	-	-	-	-	-	-	3.53	-	-	-	0.66	4.98	1.87	-	-	-	-	-	-	-	-	-	1.42
8	-	-	-	-	-	-	-	1.15	-	-	-	-	2.30	0.81	-	-	-	-	-	-	-	-	-	5.15
9	-	-	-	-	-	-	27.1	23.3	-	36.4	5.78	17.8	236	65.1	-	-	-	107	-	-	-	-	20.3	366
10	-	-	0.97	-	1.18	-	-	1.78	-	-	-	-	0.72	0.32	-	-	-	-	-	-	-	-	-	2.25

2.2. Concentration range in corn gluten matrices

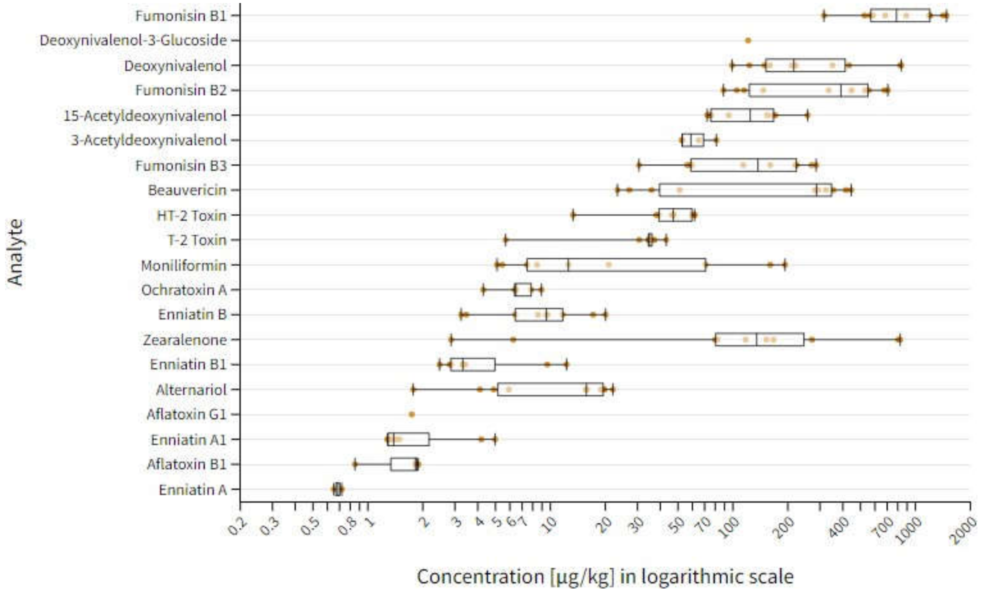

Figure S2: Graphical illustration of H15-mean based concentration ranges in corn gluten matrix. The x-axis represents the concentration range for the specific assigned values in µg/kg in a logarithmic scale. The y-axisshows the individual target compounds.

Table S3: Analyte specific H15-mean based concentration range for 10 individual corn gluten matrix lots.

Lot	15-AcDON	3-AcDON	AFB1	AFB2	AFG1	AFG2	AOH	BEA	D3G	DON	ENN-A	ENN-A1	ENN-B	ENN-B1	FB1	FB2	FB3	HT-2	MON	NIV	OTA	OTB	T-2	ZEN
1	166	65.0	-	-	-	-	15.6	416	-	351	0.72	4.98	20.1	12.3	891	447	160	39.3	20.9	-	4.29	-	34.8	825
2	154	52.7	-	-	-	-	15.9	445	-	433	0.65	4.18	17.1	9.61	683	335	114	38.0	12.5	-	8.93	-	35.9	805
3	171	52.8	-	-	-	-	4.11	51.1	-	837	-	-	-	-	564	147	59.7	13.3	70.9	-	-	-	5.68	79.8
4	-	-	1.82	-	-	-	5.92	27.1	-	210	-	1.49	3.46	2.81	585	115	58.6	59.6	160	-	6.47	-	30.7	6.26
5	257	81.2	-	-	-	-	1.77	23.3	121.1	826	-	-	6.42	-	315	88.7	30.6	-	5.45	-	-	-	-	82.2
6	74.9	-	0.85	-	-	-	18.9	293	-	98.9	-	1.35	8.55	3.32	1203	555	224	47.4	5.10	-	7.85	-	36.0	117
7	94.8	-	-	-	-	-	19.8	282	-	123	-	1.29	11.7	3.42	1189	531	220	61.7	-	-	-	-	43.1	271
8	72.3	-	-	-	-	-	19.6	355	-	159	-	1.42	9.63	3.31	1481	706	286	47.1	8.44	-	-	-	34.6	167
9	76.0	-	-	-	-	-	22.0	323	-	149	-	1.28	9.51	2.86	1417	677	270	46.3	7.45	-	-	-	37.2	153
10	-	-	1.89	-	1.74	-	4.90	35.8	-	221	-	1.29	3.24	2.47	526	105	56.2	61.6	193	-	6.37	-	34.4	2.86

2.3. Concentration range in chicken feed matrices

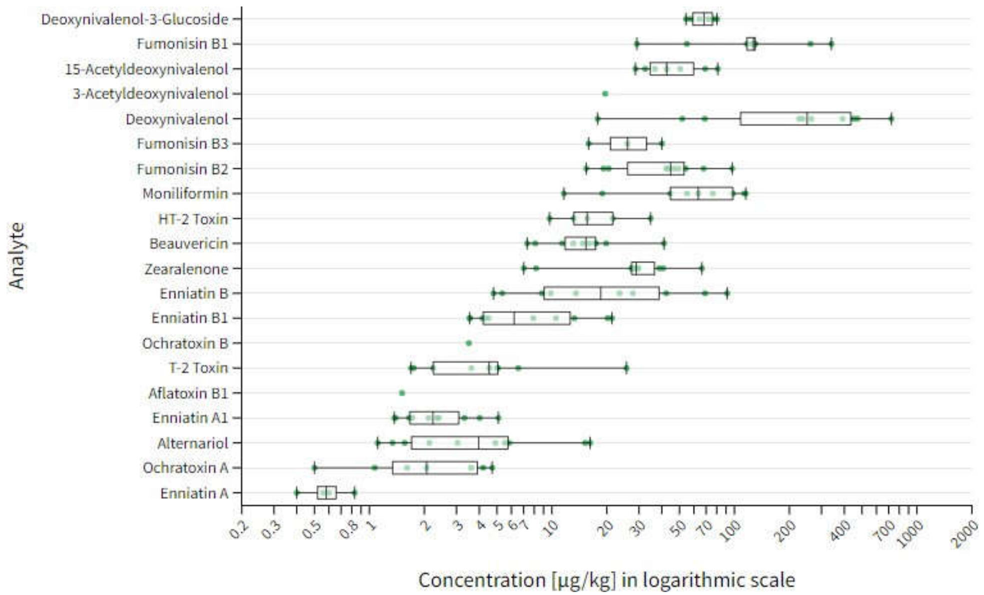

Figure S3: Graphical illustration of H15-mean based concentration ranges in chicken feed matrix. The x-axis represents the concentration range for the specific assigned values in µg/kg in a logarithmic scale. The y-axisshows the individual target compounds.

Table S4: Analyte specific H15-mean based concentration range for 10 individual chicken feed matrix lots.

Lot	15-AcDON	3-AcDON	AFB1	AFB2	AFG1	AFG2	AOH	BEA	D3G	DON	ENN-A	ENN-A1	ENN-B	ENN-B1	FB1	FB2	FB3	HT-2	MON	NIV	OTA	OTB	T-2	ZEN
1	36.7	-	-	-	-	-	15.2	11.4	54.3	449	0.56	2.40	27.8	10.5	130	43.0	-	34.7	44.6	-	4.20	-	25.6	40.7
2	-	-	-	-	-	-	2.13	8.10	-	68.8	0.83	5.09	69.0	20.2	29.2	19.2	-	-	18.9	-	-	-	4.53	8.19
3	-	-	-	-	-	-	1.11	14.8	-	51.8	0.60	4.02	91.3	21.3	-	15.4	-	-	11.6	-	4.71	3.51	2.24	29.6
4	81.1	-	1.51	-	-	-	5.83	41.2	64.3	725	-	3.32	42.3	13.3	130	42.4	15.9	21.6	63.3	-	0.50	-	6.54	66.3
5	69.2	19.6	-	-	-	-	5.53	19.8	-	264	0.40	2.35	23.5	7.92	54.9	20.5	-	15.6	-	-	-	-	3.62	38.7
6	32.4	-	-	-	-	-	4.90	13.1	77.1	237	-	2.11	8.81	4.50	261	67.9	26.0	-	55.0	-	-	-	1.69	29.7
7	28.7	-	-	-	-	-	3.04	17.4	71.9	226	-	1.72	5.34	3.56	340	97.3	40.1	-	113	-	1.61	-	1.75	27.3
8	42.6	-	-	-	-	-	1.34	16.0	57.6	392	-	1.39	9.84	4.17	117	46.5	-	13.2	98.1	-	3.61	-	5.06	28.4
9	50.4	-	-	-	-	-	1.56	17.3	80.3	472	-	1.65	13.5	4.31	127	49.4	-	9.71	116	-	2.06	-	4.99	27.5
10	-	-	-	-	-	-	16.2	7.32	-	17.8	-	1.37	4.79	3.54	126	54.2	-	-	76.1	-	1.07	-	-	7.01

2.4. Concentration range in swine feed matrices

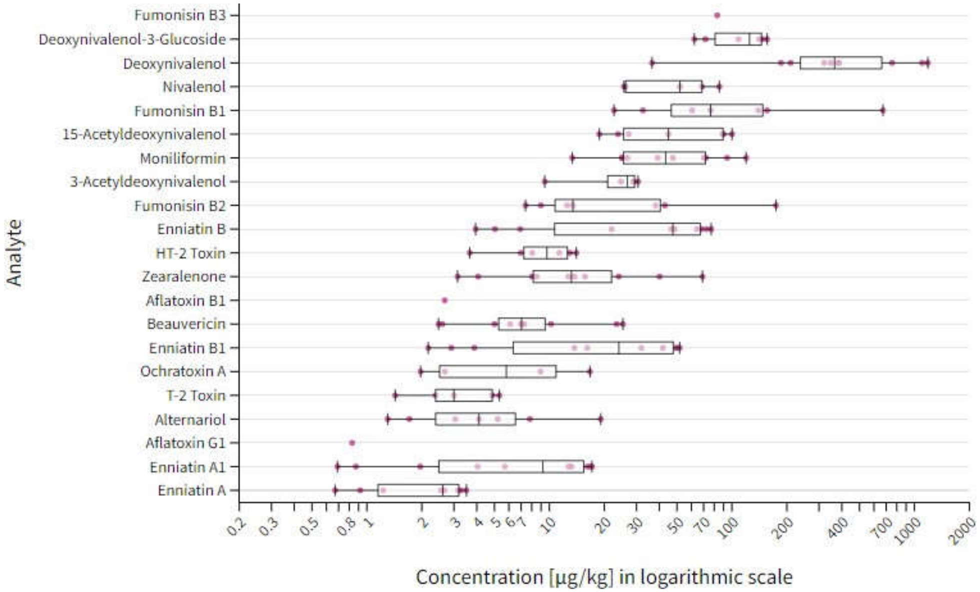

Figure S4: Graphical illustration of H15-mean based concentration ranges in swine feed matrix. The x-axis represents the concentration range for the specific assigned values in µg/kg in a logarithmic scale. The y-axisshows the individual target compounds.

Table S5: Analyte specific H15-mean based concentration range for 10 individual swine feed matrix lots.

Lot	15-AcDON	3-AcDON	AFB1	AFB2	AFG1	AFG2	AOH	BEA	D3G	DON	ENN-A	ENN-A1	ENN-B	ENN-B1	FB1	FB2	FB3	HT-2	MON	NIV	OTA	OTB	T-2	ZEN
1	-	-	-	-	-	-	1.71	5.00	-	36.5	0.67	1.96	3.94	3.88	140	42.9	-	-	94.1	-	16.7	-	1.43	4.07
2	-	-	-	-	-	-	5.21	7.28	-	186	2.55	13.2	64.4	41.8	22.6	7.40	-	14.0	26.7	68.5	-	-	5.32	12.7
3	18.8	-	-	-	-	-	19.1	10.2	-	210	3.16	16.8	72.9	49.7	32.5	8.98	-	13.0	25.0	85.3	-	-	4.86	15.7
4	-	-	-	-	-	-	7.82	6.10	109	386	3.51	17.1	77.0	51.7	60.5	12.5	-	8.05	39.3	-	1.97	-	2.38	24.0
5	89.0	28.8	-	-	-	-	4.11	6.99	156	1185	3.27	16.2	68.2	50.0	76.5	13.4	-	6.98	47.4	-	-	-	-	40.1
6	100	30.6	-	-	-	-	1.30	25.3	147	1102	2.66	12.7	48.5	31.9	156	38.2	-	11.3	72.1	25.6	8.94	-	3.00	69.0
7	27.2	9.43	2.67	-	0.83	-	3.05	7.08	-	321	1.23	5.69	21.9	13.7	673	174	83.1	-	70.2	26.1	2.68	-	-	13.7
8	90.0	24.7	-	-	-	-	-	2.59	141	755	-	0.69	5.03	2.17	-	-	-	-	25.0	-	-	-	-	8.51
9	23.8	-	-	-	-	-	-	2.47	62.2	348	-	0.87	6.92	2.90	-	-	-	-	13.4	-	-	-	-	3.14
10	44.9	-	-	-	-	-	-	23.4	71.6	382	0.92	4.04	46.4	16.1	-	-	-	3.66	120	51.9	-	-	-	8.04

3. Analyte specific z-score performance

Within the category of regulated mycotoxins, ZEN, T-2 toxin, AFB1 and HT-2 toxin showed with 79.7%,78.9%, 77.1% and 76.9% respectively a higher performance compared to the total average acceptablez-score result observed for this group. On the other hand, the performance of FB1 (68.1%), FB2 (63.8%), AFG1 (63.3%), DON (63.3%) and OTA (51.0%) was lower compared to the average value of 69.1% for this class. Amongst the group of non-regulated mycotoxins, FB3 with 93.2%, ENN-A with 88.4%, ENN- B1 with 76.7%, NIV with 75.6%, D3G with 73.9% and ENN-A1 with 72.9% were above the average performance of 70.4% in this category while MON with 67.8%, BEA with 65.3%, ENN-B with 64.8%, 15- AcDON with 64.5%, AOH with 61.6%, OTB with 50.0% and 3-AcDON with 49.4% were lower. However, for some of the compounds for both groups (regulated and non-regulated mycotoxins) with a higher performance compared to the overall average in the respective category, the number of statistical evaluable data points was rather low (e.g., AFB1 with 71, NIV with 41 or D3G with 106) compared to more prevalent toxins showing a lower performance.

3.1. Overview of individual z-score data for soy matrix

Table S6: Summary of z-score performance of 10 soy matrices. Acceptable, questionable and unacceptable z-scores are colored in green, yellow and red respectively. No-stat information refers to positive findingswhere a z-score calculation was not feasible due to a reduced number of reported results.

Lab Code	ISTD	15-AcDON	3-AcDON	AFB1	AFB2	AFG1	AFG2	AOH	BEA	D3G	DON	ENN-A	ENN-A1	ENN-B	ENN-B1	FB1	FB2	FB3	HT-2	MON	NIV	OTA	OTB	T-2	ZEN
		Lot 1																							
Lab 1	yes	-	-	-	-	-	-	-	-	-	-	-	-	-	-	-	-	-	-	-	-	-	-	-	-
Lab 1	yes	-	-	-	-	-	-	-	-	-	-	-	-	-	-	-	-	-	-	-	-	-	-	-	-
Lab 2	no	-	-	-	-	-	-	-	-1.9	-	-	-	-	-1.9	-0.4	-	-	-	-	no stat	-	-	-	-	-
Lab 2	no	-	-	-	-	-	-	-	-0.4	-	-	-	-	-0.9	1.0	-	-	-	-	no stat	-	-	-	-	-
Lab 3	yes	-	-	-	-	-	-	-	-	-	no stat	-	-	-	-	no stat	no stat	-	no stat	-	-	no stat	-	no stat	no stat
Lab 3	yes	-	-	-	-	-	-	-	-	-	no stat	-	-	-	-	no stat	no stat	-	no stat	-	-	no stat	-	no stat	no stat
Lab 4	yes	-	-	-	-	-	-	-	16.6	-	-	-	-	0.2	0.1	-	-	-	-	-	-	-	-	-	-
Lab 4	yes	-	-	-	-	-	-	-	16.3	-	-	-	-	0.2	-0.5	-	-	-	-	-	-	-	-	-	-
Lab 5	yes	-	-	-	-	-	-	-	1.8	-	-	-	-	-	-	-	-	-	-	-	-	-	-	-	-
Lab 5	yes	-	-	-	-	-	-	-	0.8	-	-	-	-	7.7	-	-	-	-	-	-	-	-	-	-	-
Lab 6	no	-	-	-	-	-	-	-	-2.0	-	-	-	no stat	2.4	1.4	-	no stat	-	-	-	-	-	-	-	-
Lab 6	no	-	-	-	-	-	-	-	-1.0	-	-	-	no stat	5.2	8.2	no stat	no stat	-	-	-	-	-	-	-	-
Lab 7	yes	-	-	-	-	-	-	-	-	-	no stat	-	-	-	-	-	-	-	-	-	-	-	-	-	-
Lab 7	yes	-	-	-	-	-	-	-	-	-	no stat	-	-	-	-	-	-	-	-	-	-	-	-	-	-
Lab 8	no	no stat	no stat	-	-	-	-	-	-2.4	-	no stat	-	-	-	-	no stat	no stat	-	no stat	-	-	-	-	-	-
Lab 8	no	no stat	no stat	-	-	-	-	-	-4.2	-	-	-	-	-	-	no stat	no stat	-	no stat	-	-	-	-	-	-
Lab 9	no	-	-	-	-	-	-	-	1.1	-	-	-	-	-0.1	0.0	-	-	-	-	-	-	-	-	-	no stat
Lab 9	no	-	-	-	-	-	-	-	0.4	-	-	-	-	-0.5	-0.1	-	-	-	-	-	-	-	-	-	no stat
Lab 10	no	-	-	-	-	-	-	-	-0.2	-	-	-	-	-0.3	-0.2	-	-	-	-	-	-	-	-	-	no stat
Lab 10	no	-	-	-	-	-	-	-	0.5	-	-	-	-	-0.3	-0.2	-	-	-	-	-	-	-	-	-	no stat

Lab Code	ISTD	15-AcDON	3-AcDON	AFB1	AFB2	AFG1	AFG2	AOH	BEA	D3G	DON	ENN-A	ENN-A1	ENN-B	ENN-B1	FB1	FB2	FB3	HT-2	MON	NIV	OTA	OTB	T-2	ZEN
		Lot 2																							
Lab 1	yes	-	-	-	-	-	-	-	-	-	-	-	-	-	-	-	-	-	-	-	-	-	-	-	-
Lab 1	yes	-	-	-	-	-	-	-	-	-	-	-	-	-	-	-	-	-	-	-	-	-	-	-	-
Lab 2	no	-	-	-2.7	-	-	-	-	0.1	-	-	-	-	-	-	-	-	-	-	-	-	-	-	-	-
Lab 2	no	-	-	-2.0	-	-	-	-	-3.5	-	-	-	-	-	-	-	-	-	-	-	-	-	-	-	-
Lab 3	yes	-	-	-2.2	-	-	-	-	-	-	no stat	-	-	-	-	no stat	no stat	-	no stat	-	-	-3.0	-	no stat	no stat
Lab 3	yes	-	-	-1.8	-	-	-	-	-	-	no stat	-	-	-	-	no stat	no stat	-	no stat	-	-	-2.9	-	no stat	no stat
Lab 4	yes	-	-	-	-	-	-	-	12.5	-	-	-	-	-	-	-	-	-	-	-	-	-	-	-	-
Lab 4	yes	-	-	-	-	-	-	-	12.3	-	-	-	-	-	-	-	-	-	-	-	-	-	-	-	-
Lab 5	yes	-	-	-0.7	-	-	-	-	-0.3	-	-	-	-	-	-	-	-	-	-	-	-	-	-	-	-
Lab 5	yes	-	-	0.6	-	-	-	-	-1.2	-	-	-	-	-	-	-	-	-	-	-	-	-	-	-	-
Lab 6	no	-	-	-	-	-	-	-	-	-	-	-	-	-	-	-	no stat	-	-	-	-	-	-	-	-
Lab 6	no	-	-	1.0	-	-	-	-	0.5	-	-	-	-	-	-	-	no stat	-	-	-	-	-	-	-	-
Lab 7	yes	-	-	18.3	no stat	-	-	-	-	-	no stat	-	-	-	-	-	-	-	-	-	-	-	-	-	-
Lab 7	yes	-	-	19.1	no stat	-	-	-	-	-	no stat	-	-	-	-	-	-	-	-	-	-	-	-	-	-
Lab 8	no	no stat	no stat	-	-	-	-	no stat	-3.2	-	-	-	-	-	-	-	-	-	no stat	-	-	-1.6	no stat	-	-
Lab 8	no	no stat	no stat	-	-	-	-	-	-	-	-	-	-	-	-	-	-	-	no stat	-	-	-2.1	no stat	-	-
Lab 9	no	-	-	0.2	-	-	-	-	-	-	-	-	-	-	-	-	-	-	-	-	-	3.1	-	-	no stat
Lab 9	no	-	-	1.0	-	-	-	-	-	-	-	-	-	-	-	-	-	-	-	-	-	2.2	-	-	no stat
Lab 10	no	-	-	0.0	-	-	-	-	-	-	-	-	-	-	-	-	-	-	-	-	-	2.3	-	-	no stat
Lab 10	no	-	-	0.3	-	-	-	-	-	-	-	-	-	-	-	-	-	-	-	-	-	1.8	-	-	no stat

Lab Code	ISTD	15-AcDON	3-AcDON	AFB1	AFB2	AFG1	AFG2	AOH	BEA	D3G	DON	ENN-A	ENN-A1	ENN-B	ENN-B1	FB1	FB2	FB3	HT-2	MON	NIV	OTA	OTB	T-2	ZEN
		**Lot 3**																							
Lab 1	yes	-	-	-	-	-	-	-	-	-	-	-	-	-	-2.1	-	-	-	1.3	-	-	-	-	1.5	0.0
Lab 1	yes	-	-	-	-	-	-	-	-	-	-	-	-	-	-1.9	-	-	-	2.3	-	-	-	-	0.3	0.0
Lab 2	no	-	-	-	-	-	-	-1.1	-3.1	-	-	-2.5	-1.4	-2.5	-0.3	-	-	-	-	-	-	-	-	-0.1	-0.4
Lab 2	no	-	-	-	-	-	-	-1.0	-2.8	-	-	-1.9	-1.9	-2.4	-1.5	-	-	-	-	-	-	-	-	-0.1	-0.7
Lab 3	yes	-	-	-	-	-	-	-	-	-	no stat	-	-	-	-	no stat	-	-	3.1	-	-	no stat	-	-0.1	0.1
Lab 3	yes	-	-	-	-	-	-	-	-	-	no stat	-	-	-	-	no stat	-	-	-1.9	-	-	no stat	-	-0.8	-1.8
Lab 4	yes	-	-	-	-	-	-	-0.5	12.3	-	-	2.3	-0.6	-0.2	-0.1	-	-	-	-1.6	-	-	-	-	-	0.1
Lab 4	yes	-	-	-	-	-	-	-0.1	13.2	-	-	2.0	-0.8	-0.3	-0.1	-	-	-	-1.6	-	-	-	-	-	0.3
Lab 5	yes	-	-	-	-	-	-	3.1	-1.3	-	-	-	6.2	2.1	7.9	-	-	-	-0.4	-	-	-	-	-	-
Lab 5	yes	-	-	-	-	-	-	5.5	0.7	-	-	-	2.1	0.2	0.9	-	-	-	4.8	-	-	-	-	-	5.5
Lab 6	no	-	-	-	-	-	-	-	-2.7	-	-	-0.9	-0.6	2.4	1.2	-	no stat	-	-	-	-	-	-	-	-0.5
Lab 6	no	-	-	-	-	-	-	-	-1.8	-	-	1.0	-0.4	-0.8	-1.5	-	no stat	-	9.6	-	-	-	-	12.7	0.7
Lab 7	yes	-	-	-	-	-	-	-	-	-	-	-	-	-	-	-	-	-	-	-	-	-	-	172.8	-
Lab 7	yes	-	-	-	-	-	-	-	-	-	-	-	-	-	-	-	-	-	-	-	-	-	-	164.8	-
Lab 8	no	no stat	no stat	-	-	-	-	-3.5	-	-	-	-	-	-0.3	-2.8	-	-	-	-0.2	-	-	no stat	-	-3.1	-4.2
Lab 8	no	no stat	no stat	-	-	-	-	-2.7	-	-	-	-	-	-2.3	-	-	-	-	0.6	-	-	no stat	-	-1.0	-4.2
Lab 9	no	-	-	-	-	-	-	0.5	2.2	-	-	-	0.9	1.4	1.9	-	-	-	-1.1	-	-	-	-	-0.3	0.6
Lab 9	no	-	-	-	-	-	-	0.8	1.2	-	-	-	0.8	1.2	1.4	-	-	-	-0.6	-	-	-	-	-0.7	0.7
Lab 10	no	-	-	-	-	-	-	0.4	-0.5	-	-	-	-	0.7	1.1	-	-	-	-1.4	-	-	-	-	-1.2	0.8
Lab 10	no	-	-	-	-	-	-	0.5	0.0	-	-	-	-	0.6	0.8	-	-	-	-1.6	-	-	-	-	-1.1	0.4

Lab Code	ISTD	15-AcDON	3-AcDON	AFB1	AFB2	AFG1	AFG2	AOH	BEA	D3G	DON	ENN-A	ENN-A1	ENN-B	ENN-B1	FB1	FB2	FB3	HT-2	MON	NIV	OTA	OTB	T-2	ZEN
		**Lot 4**																							
Lab 1	yes	-	-	-	-	-	-	-	-	-	-	-	-	-	-	-	-	-	-	-	-	-	-	-	-
Lab 1	yes	-	-	-	-	-	-	-	-	-	-	-	-	-	-	-	-	-	-	-	-	-	-	-	-
Lab 2	no	-	-	-	-	-	-	-	-3.3	-	-	-	-	-	-	-	-	-	-	-	-	-	-	-	-
Lab 2	no	-	-	-	-	-	-	-	-3.4	-	-	-	-	-	-	-	-	-	-	-	-	-	-	-	-
Lab 3	yes	-	-	-	-	-	-	-	-	-	no stat	-	-	-	-	no stat	no stat	-	no stat	-	-	no stat	-	no stat	no stat
Lab 3	yes	-	-	-	-	-	-	-	-	-	no stat	-	-	-	-	no stat	no stat	-	no stat	-	-	no stat	-	no stat	no stat
Lab 4	yes	-	-	-	-	-	-	-	8.3	-	-	-	-	-	-	-	-	-	-	-	-	-	-	-	-
Lab 4	yes	-	-	-	-	-	-	-	7.2	-	-	-	-	-	-	-	-	-	-	-	-	-	-	-	-
Lab 5	yes	-	no stat	-	-	-	-	-	-0.8	-	-	-	-	-	-	-	-	-	-	-	-	-	-	-	-
Lab 5	yes	-	-	-	-	-	-	-	-1.8	-	-	-	-	-	-	-	-	-	-	-	-	-	-	-	-
Lab 6	no	-	-	-	-	-	-	-	-	-	-	-	-	-	-	-	no stat	-	-	-	-	-	-	-	-
Lab 6	no	-	-	-	-	-	-	-	0.6	-	-	-	-	no stat	-	-	no stat	-	-	-	-	-	-	-	-
Lab 7	yes	-	-	-	-	-	-	-	-	-	-	-	-	-	-	-	-	-	-	-	-	-	-	-	-
Lab 7	yes	-	-	-	-	-	-	-	-	-	-	-	-	-	-	-	-	-	-	-	-	-	-	-	-
Lab 8	no	-	no stat	-	-	-	-	-	-	-	-	-	-	-	-	-	-	-	no stat	-	-	-	-	-	-
Lab 8	no	-	no stat	-	-	-	-	-	-	-	-	-	-	-	-	-	-	-	no stat	-	-	-	-	-	-
Lab 9	no	-	-	-	-	-	-	-	-	-	-	-	-	-	-	-	-	-	-	-	-	-	-	-	-
Lab 9	no	-	-	-	-	-	-	-	-	-	-	-	-	-	-	-	-	-	-	-	-	-	-	-	-
Lab 10	no	-	-	-	-	-	-	-	-	-	-	-	-	-	-	-	-	-	-	-	-	-	-	-	-
Lab 10	no	-	-	-	-	-	-	-	-	-	-	-	-	-	-	-	-	-	-	-	-	-	-	-	-

Lot 5

Lab Code	ISTD	15-AcDON	3-AcDON	AFB1	AFB2	AFG1	AFG2	AOH	BEA	D3G	DON	ENN-A	ENN-A1	ENN-B	ENN-B1	FB1	FB2	FB3	HT-2	MON	NIV	OTA	OTB	T-2	ZEN
Lab 1	yes	-	-	-	-	-	-	-	-	-	-	-	-	-	-	-	-	-	-	-	-	-	-	-	-
Lab 1	yes	-	-	-	-	-	-	-	-	-	-	-	-	-	-	-	-	-	-	-	-	-	-	-	-
Lab 2	no	-	-	-	-	-	-	-	1.3	-	-	-	-	-1.8	0.3	-	-	-	-	0.8	-	-	-	-	-2.5
Lab 2	no	-	-	-	-	-	-	-	-2.4	-	-	-	-	-3.3	-2.4	-	-	-	-	0.6	-	-	-	-	-1.3
Lab 3	yes	-	-	-	-	-	-	-	-	-	no stat	-	-	-	-	no stat	no stat	-	no stat	-	-	no stat	-	no stat	0.9
Lab 3	yes	-	-	-	-	-	-	-	-	-	no stat	-	-	-	-	no stat	no stat	-	no stat	-	-	no stat	-	no stat	7.7
Lab 4	yes	-	-	-	-	-	-	-	64.3	-	-	-	-	-0.6	-1.4	-	-	-	-	2.6	-	-	-	-	1.8
Lab 4	yes	-	-	-	-	-	-	-	61.9	-	-	-	-	-1.3	-1.3	-	-	-	-	2.7	-	-	-	-	0.7
Lab 5	yes	-	-	-	-	-	-	-	-0.1	-	-	-	-	-	-	-	-	-	-	-	-	-	-	-	-
Lab 5	yes	-	-	-	-	-	-	-	1.6	-	-	-	-	-	-	-	-	-	-	-	-	-	-	-	-
Lab 6	no	-	-	-	-	-	-	-	-1.6	-	-	-	no stat	16.9	7.6	-	no stat	-	-	-	-	-	-	-	15.2
Lab 6	no	-	-	-	-	-	-	-	-0.7	-	-	-	-	3.8	11.7	-	no stat	-	-	-	-	-	-	-	13.8
Lab 7	yes	-	-	-	-	-	-	-	-	-	no stat	-	-	-	-	-	-	-	-	-	-	-	-	-	-
Lab 7	yes	-	-	-	-	-	-	-	-	-	no stat	-	-	-	-	-	-	-	-	-	-	-	-	-	-
Lab 8	no	no stat	no stat	-	-	-	-	-	-	-	no stat	-	-	-	-	no stat	no stat	-	no stat	-4.3	-	-	-	-	-2.7
Lab 8	no	no stat	no stat	-	-	-	-	-	-	-	no stat	-	-	-	-	no stat	no stat	-	no stat	-4.3	-	-	-	-	-
Lab 9	no	-	-	-	-	-	-	-	0.4	-	-	-	-	0.4	-	-	-	-	-	-	-	-	-	-	-0.2
Lab 9	no	-	-	-	-	-	-	-	-1.1	-	-	-	-	0.2	-	-	-	-	-	-	-	-	-	-	-1.0
Lab 10	no	-	-	-	-	-	-	-	-1.6	-	-	-	-	0.0	-	-	-	-	-	-	-	-	-	-	-1.5
Lab 10	no	-	-	-	-	-	-	-	-0.5	-	-	-	-	0.4	-	-	-	-	-	-	-	-	-	-	-1.3

Lot 6

Lab Code	ISTD	15-AcDON	3-AcDON	AFB1	AFB2	AFG1	AFG2	AOH	BEA	D3G	DON	ENN-A	ENN-A1	ENN-B	ENN-B1	FB1	FB2	FB3	HT-2	MON	NIV	OTA	OTB	T-2	ZEN
Lab 1	yes	-	-	-	-	-	-	-	-	-	-	-	-	-	-	-	-	-	-	-	-	-	-	-	-
Lab 1	yes	-	-	-	-	-	-	-	-	-	-	-	-	-	-	-	-	-	-	-	-	-	-	-	-
Lab 2	no	-	-	-	-	-	-	-	-3.7	-	-	-	no stat	-2.3	-0.9	-	-	-	-	-	-	-3.2	-	-	-
Lab 2	no	-	-	-	-	-	-	-	-3.6	-	-	-	no stat	-2.7	-1.3	-	-	-	-	-	-	-1.0	-	-	-
Lab 3	yes	-	-	-	-	-	-	-	-	-	no stat	-	-	-	-	no stat	no stat	-	no stat	-	-	-2.7	-	no stat	-1.6
Lab 3	yes	-	-	-	-	-	-	-	-	-	no stat	-	-	-	-	no stat	no stat	-	no stat	-	-	-2.4	-	no stat	0.2
Lab 4	yes	-	-	-	-	-	-	-	5.8	-	-	-	-	0.1	-0.2	-	-	-	-	-	-	-	-	-	-
Lab 4	yes	-	-	-	-	-	-	-	5.0	-	-	-	-	0.0	0.1	-	-	-	-	-	-	-	-	-	-
Lab 5	yes	-	-	-	-	-	-	-	-2.0	-	-	-	-	2.2	-	-	-	-	-	-	-	2.6	-	-	-
Lab 5	yes	-	-	-	-	-	-	-	-	-	-	-	-	-0.2	-	-	-	-	-	-	-	-	-	-	-
Lab 6	no	-	-	-	-	-	-	-	-	-	-	-	no stat	3.5	1.8	-	no stat	-	-	-	-	12.3	-	-	-
Lab 6	no	-	-	-	-	-	-	-	0.1	-	-	-	no stat	5.3	7.1	-	no stat	-	-	-	-	-	-	-	-
Lab 7	yes	-	-	-	-	-	-	-	-	-	no stat	-	-	-	-	-	-	-	-	-	-	-	-	-	-
Lab 7	yes	-	-	-	-	-	-	-	-	-	no stat	-	-	-	-	-	-	-	-	-	-	-	-	-	-
Lab 8	no	-	no stat	-	-	-	-	-	-	-	-	-	-	19.8	6.7	-	-	-	no stat	-	-	-0.4	no stat	-	-
Lab 8	no	no stat	no stat	-	-	-	-	-	-	-	-	-	-	-	-	-	-	-	no stat	-	-	3.1	no stat	-	-
Lab 9	no	-	-	-	-	-	-	-	-	-	-	-	-	-0.9	-0.1	-	-	-	-	-	-	-	-	-	2.8
Lab 9	no	-	-	-	-	-	-	-	-	-	-	-	-	-1.0	-0.4	-	-	-	-	-	-	-	-	-	2.1
Lab 10	no	-	-	-	-	-	-	-	-	-	-	-	-	-1.5	-1.7	-	-	-	-	-	-	-	-	-	-1.0
Lab 10	no	-	-	-	-	-	-	-	-	-	-	-	-	-1.4	-1.2	-	-	-	-	-	-	-	-	-	-1.5

Lab Code	ISTD	15-AcDON	3-AcDON	AFB1	AFB2	AFG1	AFG2	AOH	BEA	D3G	DON	ENN-A	ENN-A1	ENN-B	ENN-B1	FB1	FB2	FB3	HT-2	MON	NIV	OTA	OTB	T-2	ZEN
		**Lot 7**																							
Lab 1	yes	-	-	-	-	-	-	-	-	-	-	-	-	-	-	-	-	-	-	-	-	-	-	-	-
Lab 1	yes	-	-	-	-	-	-	-	-	-	-	-	-	-	-	-	-	-	-	-	-	-	-	-	-
Lab 2	no	-	-	-	-	-	-	-	-2.3	-	-	-	-1.3	-2.0	-0.5	-	-	-	-	no stat	-	-	-	-	-
Lab 2	no	-	-	-	-	-	-	-	-2.6	-	-	-	-1.0	-1.8	-0.3	-	-	-	-	no stat	-	-	-	-	-
Lab 3	yes	-	-	-	-	-	-	-	-	-	no stat	-	-	-	-	no stat	no stat	-	no stat	-	-	no stat	-	no stat	-0.5
Lab 3	yes	-	-	-	-	-	-	-	-	-	no stat	-	-	-	-	no stat	no stat	-	no stat	-	-	no stat	-	no stat	0.1
Lab 4	yes	-	-	-	-	-	-	-	13.3	-	-	-	-1.1	-0.5	-0.7	-	-	-	-	no stat	-	-	-	-	-
Lab 4	yes	-	-	-	-	-	-	-	12.5	-	-	-	-1.2	-0.6	-0.6	-	-	-	-	no stat	-	-	-	-	-
Lab 5	yes	-	-	-	-	-	-	-	-1.2	-	-	-	-	2.6	7.4	-	-	-	-	-	-	-	-	-	-
Lab 5	yes	-	-	-	-	-	-	-	0.8	-	-	-	-	1.4	-	-	-	-	-	-	-	-	-	-	-
Lab 6	no	-	-	-	-	-	-	-	-1.8	-	-	no stat	0.9	1.8	0.8	-	no stat	-	-	-	-	-	-	-	-
Lab 6	no	-	-	-	-	-	-	-	-0.8	-	-	no stat	4.3	1.3	1.5	-	no stat	-	-	-	-	-	-	-	-
Lab 7	yes	-	-	-	-	-	-	-	-	-	no stat	-	-	-	-	-	-	-	-	-	-	-	-	-	-
Lab 7	yes	-	-	-	-	-	-	-	-	-	no stat	-	-	-	-	-	-	-	-	-	-	-	-	-	-
Lab 8	no	no stat	no stat	-	-	-	-	-	-	-	-	-	-	-	-	no stat	-	-	no stat	-	-	-	-	-	-
Lab 8	no	no stat	no stat	-	-	-	-	-	-	-	no stat	-	-	-	-	no stat	-	-	no stat	-	-	-	-	-	-
Lab 9	no	-	-	-	-	-	-	no stat	0.4	-	-	-	0.5	-0.3	0.7	-	-	-	-	-	-	-	-	-	1.6
Lab 9	no	-	-	-	-	-	-	no stat	2.1	-	-	-	1.2	-0.1	0.0	-	-	-	-	-	-	-	-	-	1.5
Lab 10	no	-	-	-	-	-	-	no stat	0.3	-	-	-	-	-0.3	-0.5	-	-	-	-	-	-	-	-	-	-0.5
Lab 10	no	-	-	-	-	-	-	no stat	-0.6	-	-	-	-	-0.3	-0.3	-	-	-	-	-	-	-	-	-	-1.1

Lab Code	ISTD	15-AcDON	3-AcDON	AFB1	AFB2	AFG1	AFG2	AOH	BEA	D3G	DON	ENN-A	ENN-A1	ENN-B	ENN-B1	FB1	FB2	FB3	HT-2	MON	NIV	OTA	OTB	T-2	ZEN
		**Lot 8**																							
Lab 1	yes	-	-	-	-	-	-	-	-	-	-	-	-	-	-	-	-	-	-	-	-	-	-	-	0.8
Lab 1	yes	-	-	-	-	-	-	-	-	-	-	-	-	-	-	-	-	-	-	-	-	-	-	-	0.8
Lab 2	no	-	-	-	-	-	-	no stat	-2.5	-	-	-	no stat	-1.0	0.2	-	-	-	-	-	-	-	-	no stat	-0.8
Lab 2	no	-	-	-	-	-	-	no stat	-2.6	-	-	-	no stat	-1.2	-0.3	-	-	-	-	-	-	-	-	no stat	-1.1
Lab 3	yes	-	-	-	-	-	-	-	-	-	no stat	-	-	-	-	no stat	no stat	-	no stat	-	-	no stat	-	no stat	-1.7
Lab 3	yes	-	-	-	-	-	-	-	-	-	no stat	-	-	-	-	no stat	no stat	-	no stat	-	-	no stat	-	no stat	0.4
Lab 4	yes	-	-	-	-	-	-	-	12.4	-	-	-	-	1.3	1.1	-	-	-	-	-	-	-	-	-	1.9
Lab 4	yes	-	-	-	-	-	-	-	10.8	-	-	-	-	1.2	1.1	-	-	-	-	-	-	-	-	-	1.8
Lab 5	yes	-	-	-	-	-	-	-	-0.7	-	-	-	-	5.1	-	-	-	-	-	-	-	-	-	-	-
Lab 5	yes	-	-	-	-	-	-	-	-1.0	-	-	-	-	-0.5	-	-	-	-	-	-	-	-	-	-	-
Lab 6	no	-	-	-	-	-	-	-	-	-	-	-	no stat	2.8	1.0	-	no stat	-	-	-	-	-	-	-	-
Lab 6	no	-	-	-	-	-	-	-	1.0	-	-	-	no stat	4.5	6.1	-	no stat	-	-	-	-	-	-	no stat	8.7
Lab 7	yes	-	-	-	-	-	-	-	-	-	-	-	-	-	-	-	-	-	-	-	-	-	-	-	-
Lab 7	yes	-	-	-	-	-	-	-	-	-	-	-	-	-	-	-	-	-	-	-	-	-	-	-	-
Lab 8	no	-	-	-	-	-	-	-	-	-	-	-	-	-	-	-	-	-	-	-	-	-	-	-	-
Lab 8	no	-	-	-	-	-	-	-	-	-	-	-	-	-	-	-	-	-	-	-	-	-	-	-	-
Lab 9	no	-	-	-	-	-	-	-	-	-	-	-	-	-0.6	-1.3	-	-	-	-	-	-	-	-	-	-0.8
Lab 9	no	-	-	-	-	-	-	-	-	-	-	-	-	-0.7	-0.6	-	-	-	-	-	-	-	-	-	-1.0
Lab 10	no	-	-	-	-	-	-	-	-	-	-	-	-	-0.8	-1.4	-	-	-	-	-	-	-	-	-	-0.5
Lab 10	no	-	-	-	-	-	-	-	-	-	-	-	-	-0.9	-2.0	-	-	-	-	-	-	-	-	-	-0.3

Lab Code	ISTD	15-AcDON	3-AcDON	AFB1	AFB2	AFG1	AFG2	AOH	BEA	D3G	DON	ENN-A	ENN-A1	ENN-B	ENN-B1	FB1	FB2	FB3	HT-2	MON	NIV	OTA	OTB	T-2	ZEN
		Lot 9																							
Lab 1	yes	-	-	-	-	-	-	1.2	-1.5	-	-	-0.9	1.0	-	-1.1	-	-	-	0.3	-	-	-	-	0.4	-0.6
Lab 1	yes	-	-	-	-	-	-	0.9	-1.8	-	-	-2.0	-0.9	-	-1.5	-	-	-	0.6	-	-	-	-	0.4	-0.7
Lab 2	no	-	-	-	-	-	-	-2.2	-1.5	-	-0.1	-1.2	-0.3	-2.0	0.1	-	-	-	1.5	-	-	-	-	1.1	0.9
Lab 2	no	-	-	-	-	-	-	-1.9	-1.4	-	-0.4	-1.0	0.1	-1.7	0.3	-	-	-	1.2	-	-	-	-	0.6	-0.1
Lab 3	yes	-	-	-	-	-	-	-	-	-	1.5	-	-	-	-	no stat	no stat	-	-0.5	-	-	no stat	-	-0.4	0.8
Lab 3	yes	-	-	-	-	-	-	-	-	-	1.8	-	-	-	-	no stat	no stat	-	-0.1	-	-	no stat	-	-0.1	0.0
Lab 4	yes	-	-	-	-	-	-	-0.7	12.7	-	-	0.6	-0.8	0.1	-0.2	-	-	-	-1.7	-	-	-	-	1.0	1.2
Lab 4	yes	-	-	-	-	-	-	-0.4	12.2	-	-	0.2	-1.3	-0.1	-0.5	-	-	-	-1.7	-	-	-	-	1.0	0.7
Lab 5	yes	-	-	-	-	-	-	9.5	-1.7	-	-	5.9	6.4	1.4	9.5	-	-	-	0.5	-	-	-	-	-	2.3
Lab 5	yes	-	-	-	-	-	-	-3.1	-0.3	-	4.8	1.1	0.2	-1.3	-0.5	-	-	-	0.6	-	-	-	-	-	2.4
Lab 6	no	-	-	-	-	-	-	-	-1.2	-	-	-0.7	-0.1	2.6	0.1	-	no stat	-	1.8	-	-	-	-	-1.4	-1.0
Lab 6	no	-	-	-	-	-	-	-2.0	-1.8	-	-	-0.4	0.6	0.7	-1.0	-	no stat	-	3.5	-	-	-	-	-0.3	-2.4
Lab 7	yes	-	-	-	-	-	-	-	-	-	-	-	-	-	-	-	-	-	0.3	-	-	-	-	72.2	-0.6
Lab 7	yes	-	-	-	-	-	-	-	-	-	-	-	-	-	-	-	-	-	0.0	-	-	-	-	70.7	-0.7
Lab 8	no	-	no stat	-	-	-	-	-0.5	0.3	-	-3.3	-	-1.0	-0.4	0.4	-	-	-	-2.1	-	-	-	-	-1.3	-3.6
Lab 8	no	-	no stat	-	-	-	-	-0.9	0.3	-	-3.4	-	-1.9	-1.0	-0.9	-	-	-	-1.9	-	-	-	-	-0.7	-3.8
Lab 9	no	-	-	-	-	-	-	1.5	1.4	-	-	-0.1	1.5	0.8	0.9	-	-	-	0.1	-	-	-	-	-1.2	1.3
Lab 9	no	-	-	-	-	-	-	1.4	1.4	-	-	-0.2	1.2	0.7	0.8	-	-	-	-0.3	-	-	-	-	-0.8	1.1
Lab 10	no	-	-	-	-	-	-	1.7	1.1	-	-	1.8	-0.6	0.3	0.8	-	-	-	-1.0	-	-	-	-	-0.4	-0.1
Lab 10	no	-	-	-	-	-	-	1.9	0.5	-	-	1.1	0.3	0.0	0.7	-	-	-	-0.6	-	-	-	-	-0.6	-0.1

Lab Code	ISTD	15-AcDON	3-AcDON	AFB1	AFB2	AFG1	AFG2	AOH	BEA	D3G	DON	ENN-A	ENN-A1	ENN-B	ENN-B1	FB1	FB2	FB3	HT-2	MON	NIV	OTA	OTB	T-2	ZEN
		Lot 10																							
Lab 1	yes	-	-	-	-	-	-	-	-	-	-	-	-	-	-	-	-	-	-	-	-	-	-	-	-
Lab 1	yes	-	-	-	-	-	-	-	-	-	-	-	-	-	-	-	-	-	-	-	-	-	-	-	-
Lab 2	no	-	-	-0.3	no stat	-2.9	-	no stat	-1.7	-	-	-	-	-3.1	-1.9	-	-	-	-	-	-	no stat	no stat	-	-1.4
Lab 2	no	-	-	-0.8	no stat	-2.5	-	no stat	-1.4	-	-	-	-	-3.3	-2.6	-	-	-	-	-	-	no stat	no stat	-	-2.2
Lab 3	yes	-	-	1.6	-	-	-	-	-	-	no stat	-	-	-	-	no stat	no stat	-	no stat	-	-	no stat	-	no stat	2.1
Lab 3	yes	-	-	0.3	-	-	-	-	-	-	no stat	-	-	-	-	no stat	no stat	-	no stat	-	-	no stat	-	no stat	0.6
Lab 4	yes	-	-	-	-	-	-	-	17.1	-	-	-	-	2.5	1.9	-	-	-	-	-	-	-	-	-	6.4
Lab 4	yes	-	-	-	-	-	-	-	16.8	-	-	-	-	1.0	1.5	-	-	-	-	-	-	-	-	-	6.2
Lab 5	yes	-	-	-	-	-	-	-	-0.6	-	-	-	-	-	-	-	-	-	-	-	-	-	-	-	-
Lab 5	yes	-	-	-	-	-	-	-	-0.2	-	-	-	-	-	-	-	-	-	-	-	-	-	-	-	-
Lab 6	no	-	-	-	-	-	-	-	2.1	-	-	-	-	1.1	-0.3	-	no stat	-	-	-	-	-	-	-	-
Lab 6	no	-	-	-	-	-	-	-	1.3	-	-	-	-	4.3	12.5	-	no stat	-	-	-	-	-	-	-	-
Lab 7	yes	-	-	15.2	no stat	0.5	-	-	-	-	-	-	-	-	-	-	-	-	-	-	-	-	-	-	-
Lab 7	yes	-	-	15.6	no stat	2.1	-	-	-	-	-	-	-	-	-	-	-	-	-	-	-	-	-	-	-
Lab 8	no	-	-	-	-	-	-	-	-	-	-	-	-	-	-	-	-	-	-	-	-	-	-	-	-
Lab 8	no	-	-	-	-	-	-	-	-	-	-	-	-	-	-	-	-	-	-	-	-	-	-	-	-
Lab 9	no	-	-	0.0	-	-0.1	-	no stat	0.3	-	-	-	-	-	-	-	-	-	-	-	-	-	-	-	0.5
Lab 9	no	-	-	-0.6	-	2.4	-	no stat	1.1	-	-	-	-	-	-	-	-	-	-	-	-	-	-	-	0.1
Lab 10	no	-	-	-0.9	-	-0.2	-	no stat	-1.9	-	-	-	-	-	-	-	-	-	-	-	-	-	-	-	-1.5
Lab 10	no	-	-	-0.5	-	0.2	-	no stat	-1.5	-	-	-	-	-	-	-	-	-	-	-	-	-	-	-	-1.8

3.2. Overview of individual z-score data for corn gluten matrix

Table S7: Summary of z-score performance of 10 corn gluten matrices. Acceptable, questionable and unacceptable z-scores are colored in green, yellow and red respectively. No-stat information refers to positivefindings where a z-score calculation was not feasible due to a reduced number of reported results.

Lab Code	ISTD	15-AcDON	3-AcDON	AFB1	AFB2	AFG1	AFG2	AOH	BEA	D3G	DON	ENN-A	ENN-A1	ENN-B	ENN-B1	FB1	FB2	FB3	HT-2	MON	NIV	OTA	OTB	T-2	ZEN
		Lot 1																							
Lab 1	yes	-	-	-	-	-	-	2.6	-	-	2.6	-	-0.5	-	-1.1	-2.7	-2.3	-2.3	-	0.4	-	2.2	-	-0.7	0.6
Lab 1	yes	-	-	-	-	-	-	2.8	-	-	3.0	-	2.0	-	-0.4	-2.4	-2.1	-2.1	-	0.4	-	2.4	-	-0.6	0.8
Lab 2	no	-2.4	-	no stat	-	no stat	-	-2.9	-1.8	no stat	-1.2	0.0	0.4	-0.1	0.9	3.3	1.5	1.9	-0.1	-0.1	-	0.6	no stat	0.5	-1.1
Lab 2	no	-2.4	-	no stat	-	no stat	-	-2.8	-1.9	no stat	-1.1	-0.3	0.3	-0.3	0.8	2.9	1.4	2.0	0.7	-0.1	-	0.1	no stat	0.4	-0.7
Lab 3	yes	-	-	-	-	-	-	-	-	-	0.0	-	-	-	-	-3.7	-2.4	-	-1.9	-	-	-0.9	-	-0.6	1.3
Lab 3	yes	-	-	-	-	-	-	-	-	-	-0.4	-	-	-	-	-3.8	-2.4	-	-1.7	-	-	-0.7	-	-0.4	1.5
Lab 4	yes	3.1	-	-	-	-	-	-1.8	11.8	-	-0.3	0.0	-2.0	-0.3	-1.4	0.1	-0.3	-0.9	-2.1	0.2	-	-	-	-0.5	-0.4
Lab 4	yes	3.4	-	-	-	-	-	-1.8	11.9	-	-0.4	0.1	-2.5	-0.5	-1.5	0.0	-0.1	-0.3	-2.3	0.3	-	-	-	-0.1	-0.6
Lab 5	yes	1.6	-	-	-	-	-	2.2	-4.9	-	-0.5	-	-	2.9	1.2	1.8	1.0	0.0	-	-	-	1.0	-	-	-1.1
Lab 5	yes	-0.1	-	-	-	-	-	0.1	-5.1	-	0.1	-	-	-3.6	-	0.8	3.6	1.7	-	-	-	-1.1	-	0.4	-0.3
Lab 6	no	-	-	-	-	-	-	-	-0.7	-	-1.8	1.2	-0.7	4.7	1.8	6.9	2.6	-	8.2	-	-	2.7	-	-1.4	-1.9
Lab 6	no	-	-	-	-	no stat	-	-	-1.7	-	-0.9	1.8	1.4	2.5	0.6	21.7	9.3	-	6.6	-	-	3.3	-	-0.4	-3.1
Lab 7	yes	-	-	-	-	-	-	-	-	-	0.1	-	-	-	-	-0.4	-1.1	-	1.1	-	-	-	-	4.5	2.1
Lab 7	yes	-	-	-	-	-	-	-	-	-	-0.1	-	-	-	-	-0.6	-1.3	-	-0.1	-	-	-	-	3.1	1.4
Lab 8	no	-2.2	-4.2	-	-	-	-	-0.8	1.1	-	-3.1	-	-	-1.2	-1.5	0.7	-0.6	-	-3.1	-2.0	-	-1.6	-	-1.6	-3.5
Lab 8	no	-2.4	-4.3	-	-	-	-	-1.1	2.1	-	-3.1	-	-	-0.4	-1.0	1.5	0.5	-	-2.8	-1.2	-	0.6	-	-1.0	-3.2
Lab 9	no	0.0	1.6	-	-	-	-	1.0	1.1	-	3.2	-0.2	1.2	0.8	0.9	-0.3	1.5	1.1	2.0	-	-	-0.3	-	1.1	2.1
Lab 9	no	-0.5	0.9	-	-	-	-	0.5	0.6	-	2.6	-0.2	1.1	0.7	0.9	-0.9	0.8	1.0	2.6	-	-	-0.3	-	0.2	1.6
Lab 10	no	1.2	0.5	-	-	-	-	1.0	0.3	-	3.7	-	-0.4	-0.3	-0.1	-1.6	-1.3	-1.0	-0.4	-	-	-2.3	-	2.0	1.1
Lab 10	no	0.7	0.1	-	-	-	-	1.0	0.1	-	2.8	-	-0.6	-0.1	-0.2	-1.5	-1.1	-1.0	0.0	-	-	-1.8	-	2.8	1.2

Lab Code	ISTD	15-AcDON	3-AcDON	AFB1	AFB2	AFG1	AFG2	AOH	BEA	D3G	DON	ENN-A	ENN-A1	ENN-B	ENN-B1	FB1	FB2	FB3	HT-2	MON	NIV	OTA	OTB	T-2	ZEN
		Lot 2																							
Lab 1	yes	-	-	-	-	-	-	2.6	-	-	3.1	-	2.3	-	-0.2	-2.6	-2.1	-1.8	-	2.7	-	1.5	-	-0.8	1.1
Lab 1	yes	-	-	-	-	-	-	2.1	-	-	2.6	-	1.2	-	-0.5	-2.4	-2.0	-2.2	-	2.3	-	1.7	-	-0.7	0.6
Lab 2	no	-2.4	-	-	-	-	-	-3.0	-2.1	-	-1.4	-0.7	-0.2	-0.2	1.0	2.6	1.5	1.7	0.2	-0.6	-	0.4	no stat	0.3	-1.0
Lab 2	no	-2.5	-	-	-	-	-	-2.9	-2.1	-	-1.6	-1.1	-0.2	-0.4	1.0	3.5	2.4	2.4	0.7	-0.3	-	0.3	no stat	0.2	-1.1
Lab 3	yes	-	-	-	-	-	-	-	-	-	0.4	-	-	-	-	-3.6	-2.5	-	-1.8	-	-	-1.1	-	-1.2	0.3
Lab 3	yes	-	-	-	-	-	-	-	-	-	0.3	-	-	-	-	-3.6	-2.6	-	-1.6	-	-	-1.0	-	-0.4	2.0
Lab 4	yes	4.2	-	-	-	-	-	-1.6	12.4	-	-0.8	0.4	-2.6	-0.5	-1.4	1.0	0.0	0.1	-	0.2	-	7.0	-	0.2	-0.5
Lab 4	yes	3.0	-	-	-	-	-	-1.4	8.9	-	-0.9	-0.5	-2.3	-1.1	-1.9	0.3	0.2	0.1	-	0.4	-	7.1	-	-0.2	-0.8
Lab 5	yes	1.6	-	-	-	-	-	1.8	-4.9	-	-0.1	-	-	1.0	0.1	0.4	0.2	-0.5	-	-	-	-0.2	-	-	0.1
Lab 5	yes	0.2	-	-	-	-	-	-0.2	-5.2	-	-0.1	-	-	-3.6	-	0.4	3.5	1.8	-	-	-	-1.8	-	0.4	-0.8
Lab 6	no	-	-	-	-	-	-	-	-1.1	-	-2.5	1.0	-0.7	4.7	1.6	8.9	1.7	-	7.1	-	-	0.3	-	-1.4	-1.8
Lab 6	no	-	-	-	-	-	-	-	-2.0	-	-2.1	1.0	1.4	2.1	0.1	23.8	10.5	-	7.3	-	-	1.6	-	-0.9	-3.2
Lab 7	yes	-	-	-	-	-	-	-	-	-	-1.1	-	-	-	-	-0.2	-1.3	-	0.1	-	-	-	-	2.5	1.2
Lab 7	yes	-	-	-	-	-	-	-	-	-	-0.6	-	-	-	-	-0.1	-0.8	-	0.8	-	-	-	-	2.7	1.6
Lab 8	no	-2.4	-4.2	-	-	-	-	-1.0	1.5	-	-2.8	-	-	-1.1	-1.5	0.8	-0.2	-	-2.9	-2.2	-	-1.9	-	-1.7	-3.4
Lab 8	no	-2.5	-4.1	-	-	-	-	-1.2	2.8	-	-2.7	-	-	-0.1	-0.6	1.5	0.7	-	-2.9	-1.3	-	0.2	-	-0.9	-3.1
Lab 9	no	0.1	0.8	-	-	-	-	1.2	1.4	-	2.4	-	1.2	0.9	0.5	-0.9	0.4	0.6	1.7	-	-	-1.6	-	0.2	1.8
Lab 9	no	0.2	-0.1	-	-	-	-	1.0	1.2	-	2.6	-	1.2	0.4	0.5	-1.2	0.4	-0.1	0.4	-	-	-1.3	-	0.1	1.5
Lab 10	no	0.0	1.5	-	-	-	-	0.8	0.1	-	3.2	-	-0.6	0.2	0.2	-1.5	-0.8	-0.9	-0.8	-	-	-2.1	-	1.1	1.3
Lab 10	no	0.5	1.1	-	-	-	-	1.4	0.1	-	3.0	-	-0.7	-0.2	0.0	-1.7	-0.6	-0.9	-0.4	-	-	-1.6	-	1.2	1.3

Lab Code	ISTD	15-AcDON	3-AcDON	AFB1	AFB2	AFG1	AFG2	AOH	BEA	D3G	DON	ENN-A	ENN-A1	ENN-B	ENN-B1	FB1	FB2	FB3	HT-2	MON	NIV	OTA	OTB	T-2	ZEN
		Lot 3																							
Lab 1	yes	-	-	-	-	-	-	-	-	-	4.7	-	-	-	-	-	-1.6	-1.4	-	-0.4	-	-	-	-0.1	0.0
Lab 1	yes	-	-	-	-	-	-	-	-	-	4.3	-	-	-	-	-	-1.4	-1.1	-	-0.3	-	-	-	-0.1	0.2
Lab 2	no	-2.7	-	-	-	-	-	-2.4	-1.3	no stat	-1.9	-	-	no stat	no stat	1.1	0.8	2.1	2.7	3.1	-	-	-	0.0	-0.7
Lab 2	no	-2.5	-	-	-	-	-	-2.6	-1.4	no stat	-2.0	-	-	no stat	no stat	1.3	0.6	1.0	2.1	2.9	-	-	-	0.1	-0.7
Lab 3	yes	-	-	-	-	-	-	-	-	-	-0.9	-	-	-	-	-3.9	-	-	-0.5	-	-	-	-	-	0.4
Lab 3	yes	-	-	-	-	-	-	-	-	-	-0.7	-	-	-	-	-3.6	-	-	-0.1	-	-	-	-	-	1.0
Lab 4	yes	3.5	-	-	-	-	-	1.5	-2.2	-	-0.2	-	-	-	-	-0.8	-1.1	0.1	-	0.2	-	-	-	-	0.4
Lab 4	yes	3.1	-	-	-	-	-	1.2	0.0	-	-0.8	-	-	-	-	-1.1	-0.8	-0.1	-	0.3	-	-	-	-	1.2
Lab 5	yes	4.1	-	-	-	-	-	3.9	-4.1	no stat	-2.6	-	-	-	-	0.4	-1.0	-0.5	-	-	-	-	-	-	1.0
Lab 5	yes	2.6	-	-	-	-	-	3.3	-4.3	no stat	1.4	-	-	-	-	0.6	1.8	2.0	-	-	-	-	-	-	1.3
Lab 6	no	-	-	-	-	-	-	-	-0.8	-	-2.1	-	-	no stat	no stat	4.9	0.7	-	-	-	-	-	-	-0.6	-1.0
Lab 6	no	-	-	-	-	-	-	-	-0.9	-	-1.4	-	no stat	no stat	no stat	8.5	4.9	-	-	-	-	-	-	4.3	-1.4
Lab 7	yes	-	-	-	-	-	-	-	-	-	1.5	-	-	-	-	1.2	-0.4	-	-	-	-	-	-	-	0.6
Lab 7	yes	-	-	-	-	-	-	-	-	-	1.9	-	-	-	-	0.4	-0.9	-	-	-	-	-	-	-	0.9
Lab 8	no	-3.1	-3.2	-	-	-	-	-0.9	2.3	-	-4.6	-	-	-	-	-0.7	-1.3	-	-2.2	-1.0	-	no stat	-	-0.1	-2.7
Lab 8	no	-3.0	-3.0	-	-	-	-	-0.7	3.1	-	-4.6	-	-	-	-	-0.2	-0.3	-	-2.1	-0.3	-	no stat	-	0.4	-2.2
Lab 9	no	-1.1	0.0	-	-	-	-	-0.1	4.2	-	2.3	-	-	-	-	1.1	1.8	-0.4	-	-	-	-	-	-	0.0
Lab 9	no	-0.6	0.8	-	-	-	-	0.4	4.3	-	2.5	-	-	-	-	2.2	1.7	1.0	-	-	-	-	-	-	0.2
Lab 10	no	-0.1	2.6	-	-	-	-	-0.6	1.1	-	1.9	-	-	-	-	-1.2	-0.2	-0.3	-	-	-	-	-	-	-0.2
Lab 10	no	-0.2	2.8	-	-	-	-	-0.2	1.5	-	2.6	-	-	-	-	-1.1	-0.4	-0.5	-	-	-	-	-	-	-0.1

Lab Code	ISTD	15-AcDON	3-AcDON	AFB1	AFB2	AFG1	AFG2	AOH	BEA	D3G	DON	ENN-A	ENN-A1	ENN-B	ENN-B1	FB1	FB2	FB3	HT-2	MON	NIV	OTA	OTB	T-2	ZEN
		Lot 4																							
Lab 1	yes	-	-	-	-	-	-	9.3	-	-	-	-	-	-	-	-1.4	-1.3	-0.8	0.1	-0.3	-	2.2	-	-0.4	-0.5
Lab 1	yes	-	-	-	-	-	-	9.2	-	-	-	-	-	-	-	-1.1	-1.0	-1.2	0.2	-0.2	-	2.6	-	-0.2	-0.5
Lab 2	no	-	-	0.8	-	no stat	-	-1.0	-0.8	-	-1.7	no stat	1.0	0.7	1.5	3.6	1.6	2.6	1.9	0.5	no stat	-0.9	-	-0.2	-2.5
Lab 2	no	-	-	2.1	-	no stat	-	-1.8	-0.9	-	-2.6	no stat	-1.2	-0.2	-0.1	2.6	0.6	3.2	2.5	0.2	no stat	-0.9	-	-0.2	-2.5
Lab 3	yes	-	-	-	-	-	-	-	-	-	2.4	-	-	-	-	-3.5	-	-	-1.8	-	-	-1.3	-	-0.6	8.9
Lab 3	yes	-	-	-	-	-	-	-	-	-	3.1	-	-	-	-	-3.6	-	-	-1.6	-	-	-1.2	-	0.0	11.8
Lab 4	yes	-	-	-	-	-	-	-0.1	-3.2	-	-	-	-	-0.9	-2.9	-0.2	-1.3	-0.8	-1.3	0.8	-	10.1	-	1.4	-0.3
Lab 4	yes	-	-	-	-	-	-	0.3	-2.5	-	-	-	-	-0.4	-2.8	-0.3	-1.2	0.1	-1.0	0.8	-	9.0	-	2.0	-0.4
Lab 5	yes	-	-	1.2	-	-	-	5.2	-4.1	-	-	-	-	0.1	-	-0.4	-1.0	-0.3	-	-	-	1.3	-	-	-
Lab 5	yes	-	-	-	-	-	-	3.3	-4.2	-	-	-	-	-1.0	-	0.1	0.8	1.3	1.0	-	-	0.9	-	3.3	-
Lab 6	no	-	-	-	-	-	-	-	0.8	-	-	-	-0.3	6.6	2.7	10.3	2.2	-	5.4	-	-	-	-	-1.6	-
Lab 6	no	-	-	-	-	no stat	-	-	1.0	-	-	-	3.7	3.7	1.8	14.8	7.3	-	5.4	-	-	3.9	-	-0.4	4.9
Lab 7	yes	-	-	-1.5	-	-	-	-	-	-	-	-	-	-	-	1.3	0.9	-	0.4	-	-	-	-	1.0	-
Lab 7	yes	-	-	-1.4	-	-	-	-	-	-	-	-	-	-	-	-1.2	-2.0	-	-0.7	-	-	-	-	1.1	-
Lab 8	no	no stat	no stat	-	-	-	-	-0.8	4.0	-	2.4	-	-	-	-	1.5	-0.3	-	-2.6	-1.1	-	-0.6	-	-0.9	-
Lab 8	no	no stat	no stat	-	-	-	-	-	-0.7	-	0.3	-	-	-4.0	-	-1.6	-0.4	-	-4.2	-0.8	-	-3.8	-	-1.1	5.8
Lab 9	no	-	-	-0.8	-	-	-	-0.6	3.5	-	-	-	-0.1	0.5	0.5	0.3	0.3	0.2	0.8	-	-	-2.4	-	0.1	-0.4
Lab 9	no	-	-	-1.1	-	-	-	-1.1	3.7	-	-	-	-0.3	0.2	1.5	-0.8	0.7	-1.0	0.6	-	-	-2.2	-	0.0	-0.1
Lab 10	no	-	-	0.4	-	no stat	-	-1.5	1.7	-	-	-	-	-0.2	-1.3	-0.1	-0.3	0.0	-0.8	-	-	-3.0	-	0.1	-1.9
Lab 10	no	-	-	0.2	-	no stat	-	-0.7	1.8	-	-	-	-	-0.1	-1.0	0.0	-0.1	-0.2	-0.3	-	-	-3.2	-	0.0	-1.7

Lab Code	ISTD	15-AcDON	3-AcDON	AFB1	AFB2	AFG1	AFG2	AOH	BEA	D3G	DON	ENN-A	ENN-A1	ENN-B	ENN-B1	FB1	FB2	FB3	HT-2	MON	NIV	OTA	OTB	T-2	ZEN
		**Lot 5**																							
Lab 1	yes	-	-	-	-	-	-	-	-	-	6.3	-	-	-	-	-1.7	-1.6	-1.3	-	-	-	-	-	-	3.4
Lab 1	yes	-	-	-	-	-	-	-	-	-	5.0	-	-	-	-	-1.7	-1.4	-0.5	-	-	-	-	-	-	2.6
Lab 2	no	-2.6	-	no stat	-	-	-	-	-1.2	-2.4	-2.0	-	-	-4.4	no stat	1.3	1.0	2.7	-	3.3	no stat	-	-	-	-0.5
Lab 2	no	-2.6	-	no stat	-	-	-	-	-1.3	-2.4	-1.8	-	-	-4.3	no stat	0.5	0.2	1.3	-	3.8	no stat	-	-	-	0.5
Lab 3	yes	-	-	-	-	-	-	-	-	-	-1.1	-	-	-	-	-3.5	-	-	-	-	-	-	-	-	0.3
Lab 3	yes	-	-	-	-	-	-	-	-	-	-1.2	-	-	-	-	-3.6	-	-	no stat	-	-	-	-	-	0.1
Lab 4	yes	3.9	-	-	-	-	-	-	-1.5	-	-0.5	-	-	8.4	-	-1.5	-1.1	-0.2	-	-0.9	-	-	-	-	-0.7
Lab 4	yes	3.5	-	-	-	-	-	-	0.6	-	-0.5	-	-	8.9	-	-1.3	-1.3	-0.2	-	-0.3	-	-	-	-	-0.3
Lab 5	yes	4.6	-	-	-	-	-	-	-4.3	-1.9	-2.1	-	-	-	-	0.7	0.1	-	-	-	-	-	-	-	0.3
Lab 5	yes	1.2	-	-	-	-	-	-	-4.4	0.2	0.7	-	-	-	-	0.3	1.4	-	-	-	-	-	-	-	0.5
Lab 6	no	-	-	-	-	-	-	-	-0.1	-	-1.7	-	-	-3.9	no stat	6.1	3.7	-	-	-	-	-	-	-	-1.1
Lab 6	no	-	-	-	-	-	-	-	-0.1	-	-1.0	-	no stat	-3.4	no stat	7.7	6.7	-	-	-	-	-	-	no stat	-1.6
Lab 7	yes	-	-	-	-	-	-	-	-	-	1.3	-	-	-	-	0.2	-1.0	-	-	-	-	-	-	-	1.8
Lab 7	yes	-	-	-	-	-	-	-	-	-	0.6	-	-	-	-	0.9	-0.6	-	-	-	-	-	-	-	0.3
Lab 8	no	-3.6	-4.2	-	-	-	-	0.0	1.2	-	-4.7	-	-	1.3	-	-0.6	-1.1	-	no stat	-3.2	-	no stat	-	no stat	-2.7
Lab 8	no	-3.5	-4.2	-	-	-	-	0.2	2.1	-	-4.6	-	-	3.2	-	0.0	-0.4	-	-	-2.7	-	-	-	no stat	-2.4
Lab 9	no	-0.9	0.7	-	-	-	-	1.7	2.8	1.2	3.5	-	-	-	-	1.3	2.3	-	-	-	-	-	-	-	0.0
Lab 9	no	-0.3	0.2	-	-	-	-	0.6	3.5	1.0	3.2	-	-	-	-	1.5	1.2	-	-	-	-	-	-	-	0.2
Lab 10	no	-0.2	1.7	-	-	-	-	-0.5	0.1	1.6	3.1	-	-	-	-	-0.4	-0.1	-	-	-	-	-	-	-	-0.1
Lab 10	no	0.5	1.9	-	-	-	-	-0.2	0.1	2.1	2.8	-	-	-	-	0.0	-0.8	-	-	-	-	-	-	-	-0.2

Lab Code	ISTD	15-AcDON	3-AcDON	AFB1	AFB2	AFG1	AFG2	AOH	BEA	D3G	DON	ENN-A	ENN-A1	ENN-B	ENN-B1	FB1	FB2	FB3	HT-2	MON	NIV	OTA	OTB	T-2	ZEN
		**Lot 6**																							
Lab 1	yes	-	-	-	-	-	-	2.1	-	-	-	-	-	-	-	-2.8	-2.1	-2.2	-	-	-	1.4	-	-0.7	0.4
Lab 1	yes	-	-	-	-	-	-	1.9	-	-	-	-	-	-	-	-2.7	-2.0	-2.0	-	-	-	1.4	-	-0.6	0.5
Lab 2	no	-2.2	-	-0.1	-	-	-	-2.8	-1.4	-	-0.7	no stat	0.0	-2.3	0.1	1.8	1.1	1.7	-0.4	0.2	-	0.0	no stat	0.2	-0.8
Lab 2	no	-2.0	-	0.5	-	-	-	-2.9	-1.3	-	-0.5	no stat	0.1	-2.3	0.2	1.3	1.6	1.6	-0.7	0.1	-	0.4	no stat	0.2	-0.7
Lab 3	yes	-	-	-	-	-	-	-	-	-	-1.5	-	-	-	-	-4.0	-2.3	-	-1.6	-	-	-1.3	-	-0.8	0.6
Lab 3	yes	-	-	-	-	-	-	-	-	-	-0.6	-	-	-	-	-4.0	-2.2	-	-1.6	-	-	-0.5	-	-0.6	-0.1
Lab 4	yes	1.2	-	-	-	-	-	-1.2	10.2	-	1.2	-	-2.7	30.7	-2.1	0.1	-0.5	-0.2	-1.8	0.1	-	-	-	-0.4	-0.7
Lab 4	yes	0.7	-	-	-	-	-	-0.7	11.1	-	1.4	-	-2.7	28.9	-1.9	0.2	-0.8	-0.2	-1.7	0.5	-	-	-	-0.1	-0.4
Lab 5	yes	-	-	-	-	-	-	3.5	-4.8	-	-0.2	-	-	0.4	1.8	0.7	0.2	-0.2	-	-	-	-0.5	-	-	0.0
Lab 5	yes	-	-	-	-	-	-	0.9	-3.9	-	0.2	-	-	-	-	0.0	2.8	1.3	3.1	-	-	-2.0	-	-0.3	-0.8
Lab 6	no	-	-	9.4	-	no stat	-	-	-0.2	-	-	no stat	-0.9	0.0	0.9	11.0	2.5	-	5.0	-	-	0.4	-	-1.8	-1.3
Lab 6	no	-	-	-	-	-	-	-	-0.9	-	-	no stat	4.2	-0.3	1.5	18.8	8.7	-	-	-	-	0.7	-	-0.1	-2.0
Lab 7	yes	-	-	-	-	-	-	-	-	-	-	-	-	-	-	0.2	-0.2	-	0.0	-	-	-	-	3.7	1.2
Lab 7	yes	-	-	-	-	-	-	-	-	-	-	-	-	-	-	0.7	0.2	-	-0.9	-	-	-	-	3.0	0.8
Lab 8	no	0.7	no stat	-	-	-	-	-1.0	0.8	-	11.2	-	-	14.4	-	-0.3	-0.6	-	-3.1	-3.4	-	-1.9	no stat	-1.4	-2.8
Lab 8	no	-0.2	no stat	-	-	-	-	-0.9	1.6	-	11.8	-	-	18.4	-	-5.6	1.2	-	-3.2	-2.5	-	0.3	-	-0.7	-2.4
Lab 9	no	-	no stat	-1.9	-	-	-	0.2	1.1	-	-	-	1.4	-0.7	0.0	-0.5	0.8	1.5	1.4	-	-	-	-	1.3	7.9
Lab 9	no	-	no stat	-1.9	-	-	-	0.0	1.6	-	-	-	1.7	-0.5	1.5	0.3	0.2	0.1	4.8	-	-	-	-	1.9	8.2
Lab 10	no	-	-	-0.8	-	-	-	0.4	-0.2	-	-2.2	-	-	-1.5	-0.9	-0.1	-1.3	-0.7	2.6	-	-	-	-	2.2	6.7
Lab 10	no	-	-	0.3	-	-	-	0.3	-0.3	-	-0.3	-	-	-1.5	-1.0	-0.5	-1.0	-0.7	2.7	-	-	-	-	2.4	6.6

Lab Code	ISTD	15-AcDON	3-AcDON	AFB1	AFB2	AFG1	AFG2	AOH	BEA	D3G	DON	ENN-A	ENN-A1	ENN-B	ENN-B1	FB1	FB2	FB3	HT-2	MON	NIV	OTA	OTB	T-2	ZEN
		Lot 7																							
Lab 1	yes	-	-	-	-	-	-	2.0	-	-	-	-	-	-	-	-2.4	-2.1	-2.3	-	-	-	-	-	-0.5	0.3
Lab 1	yes	-	-	-	-	-	-	2.2	-	-	-	-	-	-	-0.6	-2.4	-2.1	-1.9	-	-	-	-	-	-0.4	0.8
Lab 2	no	-2.0	-	no stat	-	no stat	-	-2.9	-1.3	-	-0.9	no stat	0.0	-2.0	0.6	1.5	1.6	1.4	0.1	-	-	no stat	-	0.4	-0.5
Lab 2	no	-2.1	-	no stat	-	no stat	-	-2.9	-1.7	-	-1.2	no stat	-0.8	-2.2	0.2	1.6	1.2	1.8	0.6	-	-	no stat	-	0.3	-0.8
Lab 3	yes	-	-	-	-	-	-	-	-	-	0.1	-	-	-	-	-3.8	-2.5	-	-1.5	-	-	-	-	-1.3	0.9
Lab 3	yes	-	-	-	-	-	-	-	-	-	0.8	-	-	-	-	-4.0	-2.4	-	-0.6	-	-	-	-	-0.1	0.7
Lab 4	yes	1.5	-	-	-	-	-	-0.9	8.2	-	0.8	-	-2.9	7.0	-2.0	-0.1	-0.2	-0.8	-2.2	-	-	-	-	0.6	-0.6
Lab 4	yes	0.5	-	-	-	-	-	-1.0	11.4	-	0.0	-	-2.8	6.9	-1.4	0.3	0.0	-0.5	-1.5	-	-	-	-	0.2	-0.4
Lab 5	yes	3.4	-	-	-	-	-	4.3	-4.9	-	-0.1	-	-	-0.3	1.6	-0.1	-0.3	-0.8	-	-	-	-	-	-	0.6
Lab 5	yes	-	-	-	-	-	-	0.2	-3.5	-	0.1	-	-	-	-	0.2	2.4	0.9	-	-	-	-	-	-0.9	-0.9
Lab 6	no	-	-	no stat	-	no stat	-	-	-0.6	-	-	no stat	-1.4	0.5	1.3	11.0	4.7	-	5.8	-	-	-	-	-1.1	-0.8
Lab 6	no	-	-	-	-	-	-	-	-0.9	-	-	no stat	5.0	0.1	1.6	20.2	9.3	-	5.8	-	-	-	-	-0.1	-2.3
Lab 7	yes	-	-	-	-	-	-	-	-	-	-	-	-	-	-	-0.8	-1.3	-	0.3	-	-	-	-	3.6	1.6
Lab 7	yes	-	-	-	-	-	-	-	-	-	-	-	-	-	-	-0.5	-1.3	-	0.1	-	-	-	-	3.3	0.9
Lab 8	no	-0.3	no stat	-	-	-	-	-1.0	0.9	-	8.2	-	-	1.6	-	-0.1	-0.4	-	-3.1	-	-	no stat	-	-1.6	-3.0
Lab 8	no	-0.7	no stat	-	-	-	-	-0.9	1.7	-	9.1	-	-	3.0	-	2.0	1.2	-	-3.2	-	-	no stat	-	-0.7	-2.6
Lab 9	no	-	no stat	-	-	-	-	0.4	1.6	-	-	-	2.3	-1.6	1.2	0.5	1.8	1.6	1.8	-	-	-	-	-0.2	0.9
Lab 9	no	-	no stat	-	-	-	-	0.0	1.0	-	-	-	1.5	-1.8	-0.2	0.3	0.6	1.5	0.8	-	-	-	-	0.3	0.9
Lab 10	no	-	-	-	-	-	-	0.6	-0.1	-	-1.7	-	-	-2.2	-0.7	-0.1	-1.0	-0.4	1.0	-	-	-	-	1.3	0.3
Lab 10	no	-	-	-	-	-	-	0.5	-0.1	-	-1.8	-	-	-2.3	-1.6	-0.3	-1.0	-0.7	1.7	-	-	-	-	1.2	0.5

Lab Code	ISTD	15-AcDON	3-AcDON	AFB1	AFB2	AFG1	AFG2	AOH	BEA	D3G	DON	ENN-A	ENN-A1	ENN-B	ENN-B1	FB1	FB2	FB3	HT-2	MON	NIV	OTA	OTB	T-2	ZEN
		Lot 8																							
Lab 1	yes	-	-	-	-	-	-	2.6	-	-	-	-	-	-	-	-2.4	-2.3	-2.2	-	-	-	-	-	-0.3	0.7
Lab 1	yes	-	-	-	-	-	-	2.9	-	-	-	-	-	-	-	-2.5	-2.0	-2.0	-	-	-	-	-	-0.2	0.2
Lab 2	no	-2.2	-	no stat	-	-	-	-3.0	-1.0	-	-1.5	no stat	0.4	-2.2	0.4	1.6	1.5	1.5	-0.9	-0.3	-	no stat	-	0.3	-0.4
Lab 2	no	-2.3	-	no stat	-	-	-	-2.9	-1.5	-	-2.0	no stat	-1.1	-2.2	0.1	1.8	1.6	2.2	0.0	-0.1	-	no stat	-	-0.1	-0.6
Lab 3	yes	-	-	-	-	-	-	-	-	-	3.1	-	-	-	-	-3.9	-2.4	-	-2.0	-	-	-	-	-0.4	0.4
Lab 3	yes	-	-	-	-	-	-	-	-	-	3.3	-	-	-	-	-3.7	-2.4	-	-1.2	-	-	-	-	-0.1	2.0
Lab 4	yes	1.4	-	-	-	-	-	-0.5	7.4	-	-1.3	-	-3.1	19.6	-2.2	0.3	-0.3	-0.5	-1.5	1.8	-	-	-	0.1	-1.0
Lab 4	yes	0.8	-	-	-	-	-	-0.6	6.0	-	-1.3	-	-3.3	17.4	-2.4	0.0	-0.7	-0.8	-1.1	2.0	-	-	-	-0.3	-0.8
Lab 5	yes	-	-	-	-	-	-	5.4	-5.0	-	-0.7	-	-	0.1	2.2	0.9	0.2	-0.6	-	-	-	-	-	-	1.0
Lab 5	yes	-	-	-	-	-	-	1.5	-4.1	-	-0.5	-	-	-	-	0.4	3.0	1.3	3.0	-	-	-	-	0.0	-0.2
Lab 6	no	-	-	-	-	-	-	-	-0.2	-	-	no stat	-1.3	0.3	1.1	13.2	2.5	-	6.1	-	-	-	-	-1.1	-1.3
Lab 6	no	-	-	-	-	-	-	-	-1.0	-	-	no stat	4.4	-0.5	1.2	20.5	8.3	-	6.1	-	-	-	-	-0.1	-2.2
Lab 7	yes	-	-	-	-	-	-	-	-	-	-	-	-	-	-	-0.4	-0.8	-	0.0	-	-	-	-	3.4	1.0
Lab 7	yes	-	-	-	-	-	-	-	-	-	-	-	-	-	-	0.4	-0.2	-	0.3	-	-	-	-	3.6	1.3
Lab 8	no	0.5	no stat	-	-	-	-	-0.7	0.9	-	7.8	-	-	11.3	-	-0.1	-0.3	-	-2.8	-2.0	-	no stat	-	-1.3	-2.7
Lab 8	no	0.1	-	-	-	-	-	-1.0	1.6	-	8.2	-	-	14.1	-	0.5	1.1	-	-2.9	-1.4	-	no stat	-	-0.3	-2.3
Lab 9	no	-	-	-	-	-	-	-0.6	1.0	-	-	-	2.0	-1.9	0.0	-0.2	1.1	1.3	0.3	-	-	-	-	1.0	0.9
Lab 9	no	-	-	-	-	-	-	-0.1	1.6	-	-	-	2.0	-1.7	0.2	-0.2	0.7	1.6	0.7	-	-	-	-	0.0	0.7
Lab 10	no	-	-	-	-	-	-	0.7	-0.2	-	-	-	-	-2.2	-1.0	-0.7	-1.4	-0.8	1.1	-	-	-	-	0.9	0.5
Lab 10	no	-	-	-	-	-	-	0.8	-0.3	-	-	-	-	-2.3	-0.4	-0.5	-1.1	-1.0	1.3	-	-	-	-	1.1	0.7

Lab Code	ISTD	15-AcDON	3-AcDON	AFB1	AFB2	AFG1	AFG2	AOH	BEA	D3G	DON	ENN-A	ENN-A1	ENN-B	ENN-B1	FB1	FB2	FB3	HT-2	MON	NIV	OTA	OTB	T-2	ZEN
		Lot 9																							
Lab 1	yes	-	-	-	-	-	-	2.1	-	-	-	-	-	-	-	-2.6	-2.2	-2.3	-	-	-	-	-	-0.6	0.5
Lab 1	yes	-	-	-	-	-	-	2.1	-	-	-	-	-	-	-	-2.2	-2.1	-2.3	-	-	-	-	-	-0.4	0.4
Lab 2	no	-2.5	-	no stat	-	-	-	-2.9	-1.9	-	-2.2	no stat	-0.6	-2.4	0.3	1.9	1.7	2.0	-0.7	-0.2	-	no stat	-	-0.6	-0.7
Lab 2	no	-2.1	-	no stat	-	-	-	-3.0	-1.6	-	-1.8	no stat	-0.4	-2.5	0.1	1.1	1.3	1.7	-0.1	-0.2	-	no stat	-	-0.2	-0.8
Lab 3	yes	-	-	-	-	-	-	-	-	-	3.6	-	-	-	-	-3.8	-2.1	-	-1.7	-	-	-	-	-0.9	1.8
Lab 3	yes	-	-	-	-	-	-	-	-	-	4.2	-	-	-	-	-3.7	-2.5	-	-0.9	-	-	-	-	0.5	1.3
Lab 4	yes	0.8	-	-	-	-	-	-0.5	7.4	-	-1.4	-	-3.0	17.2	-2.2	0.2	-0.6	-0.7	-2.1	1.7	-	-	-	0.6	-0.5
Lab 4	yes	1.8	-	-	-	-	-	-0.5	8.0	-	-1.4	-	-3.1	16.8	-2.1	0.1	-0.4	-0.4	-1.4	2.5	-	-	-	0.5	-0.7
Lab 5	yes	-	-	-	-	-	-	4.2	-5.0	-	-0.9	-	-	-0.2	2.1	0.9	0.3	-0.3	-	-	-	-	-	-	0.8
Lab 5	yes	-	-	-	-	-	-	1.3	-4.4	-	-0.5	-	-	-	-	0.3	2.8	1.1	2.9	-	-	-	-	-0.4	-0.4
Lab 6	no	-	-	-	-	-	-	-	-0.4	-	-	no stat	-1.0	0.0	1.6	14.3	4.6	-	5.1	-	-	-	-	-1.0	-0.7
Lab 6	no	-	-	-	-	-	-	-	-1.0	-	-	no stat	4.7	-0.5	1.8	23.1	8.9	-	-	-	-	-	-	-0.1	-2.1
Lab 7	yes	-	-	-	-	-	-	-	-	-	-	-	-	-	-	1.3	0.4	-	0.1	-	-	-	-	3.9	0.7
Lab 7	yes	-	-	-	-	-	-	-	-	-	-	-	-	-	-	0.2	-0.5	-	0.3	-	-	-	-	3.2	0.5
Lab 8	no	0.7	no stat	-	-	-	-	-0.9	1.4	-	8.7	-	-	12.9	-	0.1	-0.4	-	-2.7	-2.3	-	no stat	-	-1.2	-2.7
Lab 8	no	0.0	no stat	-	-	-	-	-1.3	1.4	-	9.2	-	-	13.5	-	1.6	1.4	-	-2.9	-1.5	-	no stat	-	-0.7	-2.5
Lab 9	no	-	-	-	-	-	-	-0.2	1.7	-	-	-	2.3	-2.0	0.3	-0.4	0.1	1.7	1.3	-	-	-	-	0.5	0.7
Lab 9	no	-	-	-	-	-	-	0.0	1.8	-	-	-	1.6	-1.9	0.3	-0.5	0.0	1.6	1.5	-	-	-	-	-0.1	0.7
Lab 10	no	-	-	-	-	-	-	0.3	-0.2	-	-	-	-	-2.6	-1.4	-1.3	-1.7	-1.0	1.5	-	-	-	-	1.1	0.5
Lab 10	no	-	-	-	-	-	-	0.3	-0.3	-	-	-	-	-2.5	-0.8	-1.2	-1.7	-1.1	1.7	-	-	-	-	0.8	0.7

Lab Code	ISTD	15-AcDON	3-AcDON	AFB1	AFB2	AFG1	AFG2	AOH	BEA	D3G	DON	ENN-A	ENN-A1	ENN-B	ENN-B1	FB1	FB2	FB3	HT-2	MON	NIV	OTA	OTB	T-2	ZEN
		Lot 10																							
Lab 1	yes	-	-	-	-	-	-	-	-	-	-	-	-	-	-	-0.9	-1.4	-1.2	-0.3	-0.1	-	1.4	-	-0.6	-
Lab 1	yes	-	-	-	-	-	-	-	-	-	-	-	-	-	-	-1.0	-1.1	-1.1	0.2	0.0	-	1.9	-	-0.4	-
Lab 2	no	-	-	0.4	no stat	-0.1	-	-3.3	-0.4	-	-3.8	no stat	-0.5	-0.2	0.5	4.2	1.2	4.1	1.2	0.4	-	-0.4	-	0.1	-2.4
Lab 2	no	-	-	0.2	no stat	0.9	-	-3.4	-1.0	-	-3.4	no stat	0.3	0.4	1.7	2.8	0.6	2.7	1.0	0.0	-	-0.2	-	-0.4	-1.5
Lab 3	yes	-	-	-	-	-	-	-	-	-	3.7	-	-	-	-	-3.3	-	-	-0.6	-	-	-1.5	-	0.0	9.2
Lab 3	yes	-	-	-	-	-	-	-	-	-	3.8	-	-	-	-	-3.3	-	-	-0.7	-	-	0.2	-	1.3	17.8
Lab 4	yes	-	-	-	-	-	-	2.6	-3.4	-	-	-	-	-1.4	-2.9	-0.1	-0.5	-0.5	-1.2	2.0	-	15.5	-	0.8	0.0
Lab 4	yes	-	-	-	-	-	-	3.6	-1.6	-	-	-	-	-1.4	-2.7	-0.1	-0.5	-0.6	-1.1	2.0	-	17.3	-	0.2	-0.7
Lab 5	yes	-	-	0.5	-	-	-	5.7	-4.3	-	-3.2	-	-	0.9	-	-0.2	-0.9	-	-	-1.4	-	0.9	-	-	-
Lab 5	yes	-	-	-	-	-	-	5.8	-4.0	-	-3.0	-	-	-1.3	-	-0.1	0.8	0.5	1.4	-	-	-0.2	-	0.3	-
Lab 6	no	-	-	-	-	0.9	-	-	1.4	-	-	no stat	-1.0	5.3	1.5	15.5	4.5	-	5.1	-	-	-	-	-1.2	-
Lab 6	no	-	-	2.9	-	7.8	-	-	-0.5	-	-	no stat	0.7	1.5	1.3	17.3	5.0	-	8.0	-	-	0.4	no stat	-0.2	-
Lab 7	yes	-	-	-1.3	-	-	-	-	-	-	-	-	-	-	-	-0.3	-1.5	-	0.2	-	-	-	-	2.2	-
Lab 7	yes	-	-	-0.9	-	-	-	-	-	-	-	-	-	-	-	0.0	-1.6	-	0.9	-	-	-	-	3.2	-
Lab 8	no	no stat	no stat	-	-	-	-	-1.7	1.6	-	3.3	-	-	-	-	0.4	-1.4	-	-3.0	-1.9	-	-1.8	-	-1.9	-
Lab 8	no	no stat	no stat	-	-	-	-	-1.8	5.2	-	2.6	-	-	-	-	1.2	0.2	-	-2.7	-1.1	-	2.1	-	-0.5	-
Lab 9	no	-	-	-0.9	-	-	-	-0.9	3.0	-	-	-	0.6	0.7	1.1	-0.1	0.9	-0.2	0.0	-	-	-1.5	-	0.3	1.6
Lab 9	no	-	-	-0.2	-	-	-	-0.2	3.8	-	-	-	0.9	0.4	0.6	0.5	1.3	0.9	0.2	-	-	-2.1	-	0.4	0.9
Lab 10	no	-	-	0.0	-	-3.2	-	-0.4	0.4	-	-	-	-	-1.1	-2.1	-0.4	0.0	-0.2	-0.8	-	-	-2.9	-	-0.5	-1.0
Lab 10	no	-	-	0.8	-	-3.0	-	-2.0	0.5	-	-	-	-	-1.2	-1.3	-0.3	-0.4	-0.5	-0.5	-	-	-2.8	-	-0.7	-1.1

3.3. Overview of individual z-score data for chicken feed matrix

Table S8: Summary of z-score performance of 10 chicken feed matrices. Acceptable, questionable and unacceptable z-scores are colored in green, yellow and red respectively. No-stat information refers to positivefindings where a z-score calculation was not feasible due to a reduced number of reported results.

Lab Code	ISTD	15-AcDON	3-AcDON	AFB1	AFB2	AFG1	AFG2	AOH	BEA	D3G	DON	ENN-A	ENN-A1	ENN-B	ENN-B1	FB1	FB2	FB3	HT-2	MON	NIV	OTA	OTB	T-2	ZEN
		Lot 1																							
Lab 1	yes	-	-	-	-	-	-	-	-1.0	-	0.8	-	-	-	-1.3	-1.3	-1.8	-	1.5	0.0	-	-	-	-0.1	0.5
Lab 1	yes	-	-	-	-	-	-	-	0.7	-	-	-	-	-	-1.3	-1.5	-1.6	-	2.2	-0.1	-	-	-	0.1	0.5
Lab 2	no	0.7	no stat	-	-	-	-	-2.3	-1.7	-1.3	-2.8	-1.2	-1.0	-2.7	-1.8	0.2	2.5	no stat	5.1	2.3	no stat	-4.2	-	4.5	-1.6
Lab 2	no	0.0	no stat	-	-	-	-	-3.1	-1.6	-0.6	-2.2	-1.1	-1.0	-2.4	-1.7	2.1	2.6	no stat	1.0	2.7	no stat	-4.2	-	1.6	-1.4
Lab 3	yes	-	-	-	-	-	-	-	-	-	-1.5	-	-	-	-	-3.0	-3.0	-	0.4	-	-	-4.1	-	-0.6	-0.9
Lab 3	yes	-	-	-	-	-	-	-	-	-	-1.6	-	-	-	-	-2.7	-2.8	-	-1.6	-	-	-4.1	-	-0.8	-1.2
Lab 4	yes	-	-	-	-	-	-	-2.5	12.8	0.1	2.9	0.3	-1.8	-1.9	-2.4	-0.3	-0.9	no stat	-1.5	-0.5	-	5.4	-	2.2	1.5
Lab 4	yes	-	-	-	-	-	-	-2.7	11.8	-0.4	2.4	-0.3	-2.2	-2.0	-2.3	-0.2	-1.4	no stat	-1.9	-0.3	-	7.0	-	2.0	1.7
Lab 5	yes	-	-	-	-	-	-	3.8	-2.6	1.2	0.0	-	-	-0.7	1.2	0.9	-0.3	-	-	-	-	-	-	-	0.9
Lab 5	yes	-	-	-	-	-	-	2.1	-2.6	1.0	-0.2	-	-	-3.6	-	0.8	0.5	-	-	-	-	-	-	-	2.7
Lab 6	no	-	-	-	-	-	-	-	-0.6	-	-0.8	1.2	1.0	1.7	-0.2	11.4	8.1	-	-	-	-	-	-	-0.1	7.7
Lab 6	no	-	-	-	-	-	-	-0.9	-0.2	-	-0.6	1.2	1.0	0.5	-0.7	11.4	11.3	-	7.5	-	-	-	-	0.6	0.1
Lab 7	yes	-	-	-	-	-	-	-	-	-	5.1	-	-	-	-	2.5	2.4	-	-	-	-	-	-	-	0.8
Lab 7	yes	-	-	-	-	-	-	-	-	-	1.0	-	-	-	-	-2.3	-2.0	-	-	-	-	-	-	-	-0.3
Lab 8	no	-1.0	no stat	-	-	-	-	-0.3	9.6	-	-3.3	-	9.7	-1.4	0.2	-0.8	0.2	-	-1.0	-0.7	-	2.5	-	-0.3	-1.0
Lab 8	no	-0.3	no stat	-	-	-	-	0.1	9.3	-	-2.9	-	9.5	-1.4	0.0	-0.9	-0.4	-	-1.0	-	-	2.5	-	0.1	-1.2
Lab 9	no	0.8	-	-	-	-	-	0.4	0.8	-	1.6	-	0.4	14.3	2.4	0.1	-	-	0.4	-	-	-	-	-0.1	-0.1
Lab 9	no	-0.8	-	-	-	-	-	0.2	0.8	-	1.3	-	-0.5	13.0	2.2	0.6	-	-	-0.5	-	-	-	-	-1.0	-0.3
Lab 10	no	0.5	-	-	-	-	-	0.7	-0.7	-	1.5	-	-	10.5	3.2	-0.1	-	-	-0.8	-	-	-	-	-0.2	-0.8
Lab 10	no	0.2	-	-	-	-	-	0.7	-0.7	-	1.1	-	-	9.5	2.9	0.5	-	-	-1.3	-	-	-	-	-0.3	-0.8

Lab Code	ISTD	15-AcDON	3-AcDON	AFB1	AFB2	AFG1	AFG2	AOH	BEA	D3G	DON	ENN-A	ENN-A1	ENN-B	ENN-B1	FB1	FB2	FB3	HT-2	MON	NIV	OTA	OTB	T-2	ZEN
		Lot 2																							
Lab 1	yes	-	-	-	-	-	-	-	-0.1	-	-	-	0.4	-	-0.4	-2.1	-	-	-	0.7	-	-	-	-	-0.4
Lab 1	yes	-	-	-	-	-	-	-	2.0	-	-	-	0.4	-	-0.2	-2.2	-	-	-	0.8	-	-	-	-	-0.3
Lab 2	no	-	-	no stat	-	no stat	-	-2.3	-1.2	no stat	-0.2	-1.0	-0.5	-1.6	-0.1	1.2	0.2	-	-	-0.8	no stat	no stat	no stat	-2.6	-2.1
Lab 2	no	-	-	no stat	-	no stat	-	-1.1	-1.9	no stat	-0.4	-1.0	-0.1	-1.3	0.6	1.0	-1.0	-	-	2.5	no stat	no stat	no stat	0.2	-2.0
Lab 3	yes	-	-	no stat	-	-	-	-	-	-	-0.6	-	-	-	-	-3.5	-3.9	-	no stat	-	-	no stat	-	-1.9	3.0
Lab 3	yes	-	-	no stat	-	-	-	-	-	-	-0.8	-	-	-	-	-3.4	-3.9	-	no stat	-	-	no stat	-	-2.2	3.6
Lab 4	yes	-	-	-	-	-	-	-	-3.0	-	3.5	-	-3.6	-1.9	-2.9	0.5	-	-	-	-1.6	-	-	-	0.5	-0.6
Lab 4	yes	-	-	-	-	-	-	-	-2.6	-	1.9	-	-3.5	-1.8	-2.9	0.6	-	-	-	-1.6	-	-	-	0.1	0.0
Lab 5	yes	-	-	-	-	-	-	-	-3.5	-	2.3	-	1.6	1.9	4.6	0.5	-	-	-	-	-	-	-	-	-
Lab 5	yes	-	-	-	-	-	-	-	-3.4	-	0.5	-	-	-2.8	-2.5	-	-	-	-	-	-	-	-	-	-
Lab 6	no	-	-	-	-	no stat	-	-	-1.3	-	-	1.0	1.2	3.4	1.5	15.7	12.1	-	-	-	-	-	-	-	-
Lab 6	no	-	-	-	-	-	-	-	-1.1	-	-	1.5	2.2	4.0	2.2	-	16.1	-	-	-	-	-	-	5.2	4.3
Lab 7	yes	-	-	-	-	-	-	-	-	-	-	-	-	-	-	-	-	-	-	-	-	-	-	-	-
Lab 7	yes	-	-	-	-	-	-	-	-	-	-	-	-	-	-	-	-	-	-	-	-	-	-	-	-
Lab 8	no	no stat	no stat	no stat	-	no stat	-	14.5	3.0	-	-2.0	-	3.6	-0.7	0.1	-0.2	-0.2	-	no stat	-	-	no stat	no stat	-0.2	0.3
Lab 8	no	no stat	no stat	no stat	-	no stat	-	17.4	3.5	-	-1.2	-	4.2	1.5	2.0	0.1	-0.2	-	no stat	-	-	no stat	no stat	-0.1	0.4
Lab 9	no	-	-	-	-	-	-	1.5	2.5	-	-	-0.1	-0.3	1.1	0.0	-	-	-	-	-	-	-	-	1.6	-0.3
Lab 9	no	-	-	-	-	-	-	-0.2	3.8	-	-	0.2	-0.5	0.9	0.0	-	-	-	-	-	-	-	-	0.0	-0.1
Lab 10	no	-	-	-	-	-	-	-1.3	1.8	-	-	0.2	-2.2	-0.5	-0.6	-	-	-	no stat	-	-	-	-	-0.2	0.5
Lab 10	no	-	-	-	-	-	-	-2.2	1.5	-	-	-0.9	-2.0	-0.2	-0.5	-	-	-	no stat	-	-	-	-	0.7	-0.1

Lab Code	ISTD	15-AcDON	3-AcDON	AFB1	AFB2	AFG1	AFG2	AOH	BEA	D3G	DON	ENN-A	ENN-A1	ENN-B	ENN-B1	FB1	FB2	FB3	HT-2	MON	NIV	OTA	OTB	T-2	ZEN
		Lot 3																							
Lab 1	yes	-	-	-	-	-	-	-	0.3	-	-	-	0.9	-	-0.6	-	-	-	-	-0.2	-	-0.6	-0.4	-	0.1
Lab 1	yes	-	-	-	-	-	-	-	0.7	-	-	-	0.9	-	-0.5	-	-	-	-	-0.2	-	-0.2	-0.5	-	-0.2
Lab 2	no	-	-	no stat	-	no stat	-	-1.2	-1.7	no stat	-0.8	0.2	-0.5	-1.4	0.0	no stat	0.4	-	-	1.2	no stat	-1.6	0.5	-1.5	-1.9
Lab 2	no	-	-	no stat	-	no stat	-	-1.3	-1.6	no stat	-0.1	0.2	0.1	-1.6	-0.1	no stat	-0.4	-	-	1.0	no stat	-0.9	-0.1	-1.2	-1.9
Lab 3	yes	-	-	no stat	-	-	-	-	-	-	-1.4	-	-	-	-	no stat	-3.5	-	no stat	-	-	-1.8	-	1.0	-0.6
Lab 3	yes	-	-	no stat	-	-	-	-	-	-	-1.4	-	-	-	-	no stat	-3.6	-	no stat	-	-	-1.8	-	1.8	-0.3
Lab 4	yes	-	-	-	-	-	-	-	4.8	-	1.7	-1.0	-3.0	-2.1	-2.7	-	-1.1	-	-	-0.9	-	10.1	-	-	0.2
Lab 4	yes	-	-	-	-	-	-	-	4.8	-	2.0	-1.1	-3.1	-2.0	-2.7	-	-1.9	-	-	-1.0	-	8.9	-	-	0.0
Lab 5	yes	-	-	-	-	-	-	-	-3.2	-	1.3	-	0.6	0.0	2.3	-	-	-	-	-	-	1.2	0.8	-	0.7
Lab 5	yes	-	-	-	-	-	-	-	-2.6	-	2.1	-	-	-2.1	-2.0	-	-	-	-	-	-	-0.3	9.1	-	1.2
Lab 6	no	-	-	-	-	-	-	-	-1.2	-	-	0.0	-0.6	1.4	0.6	-	15.5	-	-	-	-	1.7	0.0	-	1.3
Lab 6	no	-	-	-	-	-	-	-	-1.5	-	-	2.3	0.2	0.9	-0.1	-	16.4	-	-	-	-	0.5	0.9	-	0.2
Lab 7	yes	-	-	-	-	-	-	-	-	-	-	-	-	-	-	-	-	-	-	-	-	-	-	-	1.9
Lab 7	yes	-	-	-	-	-	-	-	-	-	-	-	-	-	-	-	-	-	-	-	-	-	-	-	1.7
Lab 8	no	no stat	no stat	no stat	-	no stat	-	30.5	2.1	-	-2.1	-	5.7	-0.3	0.5	no stat	1.4	-	no stat	-	-	8.2	9.2	2.9	-1.5
Lab 8	no	no stat	no stat	no stat	-	no stat	-	31.4	2.1	-	-1.8	-	5.3	-1.0	-0.1	no stat	1.5	-	no stat	-	-	10.8	14.2	2.7	-1.4
Lab 9	no	-	-	-	-	-	-	-1.7	0.0	-	-	-0.4	-1.2	2.5	0.2	-	-	-	-	-	-	-1.1	-3.2	-	0.6
Lab 9	no	-	-	-	-	-	-	0.0	0.5	-	-	0.6	-0.9	2.3	0.5	-	-	-	-	-	-	-2.3	-3.0	-	0.3
Lab 10	no	-	-	no stat	-	-	-	-0.7	0.0	-	0.6	0.6	-	1.9	1.1	-	-	-	-	-	-	-2.0	-3.6	-2.6	-0.4
Lab 10	no	-	-	no stat	-	-	-	1.0	-0.3	-	-0.1	-0.5	-	1.5	1.1	-	-	-	-	-	-	-2.1	-3.7	-3.2	-0.5

Lab Code	ISTD	15-AcDON	3-AcDON	AFB1	AFB2	AFG1	AFG2	AOH	BEA	D3G	DON	ENN-A	ENN-A1	ENN-B	ENN-B1	FB1	FB2	FB3	HT-2	MON	NIV	OTA	OTB	T-2	ZEN
		Lot 4																							
Lab 1	yes	-	-	-	-	-	-	-	0.5	-	2.4	-	-	-	-0.2	0.1	-1.0	0.2	-	-0.5	-	-	-	-1.3	0.7
Lab 1	yes	-	-	-	-	-	-	-	1.6	-	2.2	-	-0.2	-	-0.1	-0.1	-0.9	0.5	-	-0.5	-	-	-	-0.4	0.3
Lab 2	no	-0.6	no stat	-1.3	-	-	-	-2.5	-1.4	-0.9	-1.5	no stat	0.0	-0.4	0.3	0.8	1.7	1.4	1.3	1.2	no stat	-3.3	-	0.7	-1.7
Lab 2	no	-1.0	no stat	-0.7	-	-	-	-2.1	-1.1	-1.0	-1.6	no stat	-0.3	-0.3	0.2	-0.6	0.7	-1.0	1.4	1.1	no stat	-2.2	-	0.7	-1.7
Lab 3	yes	-	-	-0.6	-	-	-	-	-	-	-1.7	-	-	-	-	-2.7	-3.1	-	-1.5	-	-	0.3	-	-1.0	0.0
Lab 3	yes	-	-	-1.6	-	-	-	-	-	-	-1.8	-	-	-	-	-2.7	-3.0	-	-0.2	-	-	-0.5	-	-1.0	-0.2
Lab 4	yes	2.7	-	-	-	-	-	-2.4	0.0	-0.6	1.5	-	-4.0	-3.1	-3.8	-0.5	-1.5	-1.2	-	-0.2	-	-	-	0.0	-0.3
Lab 4	yes	3.1	-	-	-	-	-	-2.5	2.7	0.2	1.3	-	-3.8	-2.6	-3.5	0.0	-1.2	0.1	-	-0.3	-	-	-	0.7	0.2
Lab 5	yes	4.5	-	-	-	-	-	3.4	-4.3	2.4	0.2	-	-	-2.4	-2.7	1.1	-0.9	-	-	-	-	-	-	-	0.5
Lab 5	yes	-	-	-	-	-	-	0.7	-4.0	2.9	0.6	-	-	-3.6	-	-0.3	-0.2	-	-	-	-	-	-	-	0.1
Lab 6	no	-	-	3.0	-	-	-	-	0.4	-	0.8	no stat	0.1	6.2	2.3	13.8	8.3	-	-	-	-	-	-	0.0	2.2
Lab 6	no	-	-	-	-	-	-	-	-0.5	-	0.0	no stat	0.5	4.0	1.3	10.6	11.5	-	-	-	-	-	-	3.1	-0.2
Lab 7	yes	-	-	-	-	-	-	-	-	-	2.5	-	-	-	-	1.1	4.6	-	-	-	-	-	-	-	2.2
Lab 7	yes	-	-	-	-	-	-	-	-	-	2.1	-	-	-	-	0.4	3.7	-	-	-	-	-	-	-	2.4
Lab 8	no	-1.4	no stat	15.1	-	-	-	2.9	1.7	-	-3.9	-	6.4	3.1	1.5	-0.9	-0.3	-	-0.5	1.4	-	55.9	-	-0.1	-1.4
Lab 8	no	-1.5	no stat	15.3	-	-	-	3.2	1.8	-	-4.0	-	6.1	0.4	0.6	-0.7	0.1	-	-0.3	-	-	55.6	-	0.2	-1.8
Lab 9	no	-	-	-	-	-	-	-	-	-	-	-	-	-	-	-	-	-	-	-	-	-	-	-	-
Lab 9	no	-	-	-	-	-	-	-	-	-	-	-	-	-	-	-	-	-	-	-	-	-	-	-	-
Lab 10	no	-	-	-	-	-	-	-	-	-	-	-	-	-	-	-	-	-	-	-	-	-	-	-	-
Lab 10	no	-	-	-	-	-	-	-	-	-	-	-	-	-	-	-	-	-	-	-	-	-	-	-	-

Lab Code	ISTD	15-AcDON	3-AcDON	AFB1	AFB2	AFG1	AFG2	AOH	BEA	D3G	DON	ENN-A	ENN-A1	ENN-B	ENN-B1	FB1	FB2	FB3	HT-2	MON	NIV	OTA	OTB	T-2	ZEN
		Lot 5																							
Lab 1	yes	-	-	-	-	-	-	-	-1.6	-	-	-	-	-	-2.1	-3.0	-	-	-	-	-	-	-	-	-0.6
Lab 1	yes	-	-	-	-	-	-	-	-2.3	-	-	-	-	-	-1.6	-3.0	-	-	-	-	-	-	-	-	-0.8
Lab 2	no	-1.1	-	-	-	-	-	-1.1	-2.7	no stat	-3.0	-1.6	-1.4	-2.7	-1.5	-1.4	-0.9	-	-	no stat	no stat	-	-	-1.8	-2.9
Lab 2	no	-0.7	-	-	-	-	-	-1.6	-3.0	no stat	-2.7	-1.4	-1.4	-2.8	-1.5	-1.4	0.2	-	-	no stat	no stat	-	-	-2.0	-2.6
Lab 3	yes	-	-	-	-	-	-	-	-	-	-2.4	-	-	-	-	-3.3	-3.7	-	0.0	-	-	no stat	-	-2.3	-1.2
Lab 3	yes	-	-	-	-	-	-	-	-	-	-2.6	-	-	-	-	-3.8	-3.8	-	-0.7	-	-	no stat	-	-2.2	-1.4
Lab 4	yes	-	-	-	-	-	-	-0.3	3.2	-	-0.1	-	-2.3	-1.6	-2.0	-	-	-	-	no stat	-	-	-	-	1.1
Lab 4	yes	-	-	-	-	-	-	-0.1	1.8	-	-0.4	-	-2.7	-1.9	-2.3	-	-	-	-	no stat	-	-	-	-	0.6
Lab 5	yes	-	-	-	-	-	-	7.5	-3.4	-	-1.4	-	1.8	-0.1	3.4	-1.6	-	-	-	-	-	-	-	-	0.0
Lab 5	yes	-	-	-	-	-	-	6.9	-3.8	-	-1.4	-	-	-2.8	-2.7	-1.8	-	-	-	-	-	-	-	-	3.0
Lab 6	no	-	-	-	-	-	-	-	-2.5	-	-	-1.1	-0.9	-0.1	-0.5	7.0	9.9	-	-	-	-	-	-	-	0.0
Lab 6	no	-	-	-	-	-	-	-	-2.0	-	0.1	2.3	0.5	0.9	0.7	7.9	11.2	-	-	-	-	-	-	5.4	-0.4
Lab 7	yes	-	-	-	-	-	-	-	-	-	1.5	-	-	-	-	-	-	-	-	-	-	-	-	-	-
Lab 7	yes	-	-	-	-	-	-	-	-	-	1.1	-	-	-	-	-	-	-	-	-	-	-	-	-	-
Lab 8	no	-1.6	-3.6	-	-	-	-	4.0	-0.8	-	-3.0	-	9.4	-1.2	0.7	-1.2	-0.1	-	2.9	-	-	-	-	1.6	-2.1
Lab 8	no	-1.8	-3.7	-	-	-	-	3.8	-0.6	-	-3.7	-	9.5	-1.0	0.9	-1.5	-0.3	-	2.5	-	-	-	-	0.4	-2.5
Lab 9	no	0.7	3.3	-	-	-	-	-0.5	7.0	-	15.0	1.3	0.0	4.5	1.7	5.7	-	-	-	-	-	-	-	1.4	3.4
Lab 9	no	1.5	3.7	-	-	-	-	-0.9	8.9	-	15.4	0.7	0.3	4.8	2.1	6.2	-	-	-	-	-	-	-	0.5	3.5
Lab 10	no	1.2	0.1	no stat	-	-	-	-1.2	6.1	no stat	16.1	-	-	4.7	2.6	4.2	-	-	-2.4	-	-	-	-	0.4	1.6
Lab 10	no	1.7	0.2	no stat	-	-	-	-1.8	5.5	no stat	14.7	-	-	4.2	2.1	4.5	-	-	-2.2	-	-	-	-	1.0	1.7

Lab Code	ISTD	15-AcDON	3-AcDON	AFB1	AFB2	AFG1	AFG2	AOH	BEA	D3G	DON	ENN-A	ENN-A1	ENN-B	ENN-B1	FB1	FB2	FB3	HT-2	MON	NIV	OTA	OTB	T-2	ZEN
		Lot 6																							
Lab 1	yes	-	-	-	-	-	-	-	1.8	-	-	-	-	-	-0.5	-1.5	-0.4	-1.6	-	-0.5	-	-	-	-	-0.3
Lab 1	yes	-	-	-	-	-	-	-	2.5	17.8	-	-	-	-	-0.5	-1.3	-1.1	-1.2	-	-0.5	-	-	-	-	-0.3
Lab 2	no	-1.1	-	-	-	-	-	-3.7	0.1	-2.0	-0.9	no stat	-1.0	-1.9	0.0	0.4	0.1	2.0	no stat	1.8	no stat	-	-	-0.4	-1.4
Lab 2	no	-0.7	-	-	-	-	-	-3.9	0.0	-2.0	-1.0	no stat	-0.9	-2.0	-0.2	0.3	0.2	1.7	no stat	1.8	no stat	-	-	-0.9	-1.7
Lab 3	yes	-	-	-	-	-	-	-	-	-	-0.1	-	-	-	-	-2.7	-2.2	-	no stat	-	-	no stat	-	-1.3	0.1
Lab 3	yes	-	-	-	-	-	-	-	-	-	-0.1	-	-	-	-	-2.9	-2.4	-	no stat	-	-	no stat	-	0.4	-0.2
Lab 4	yes	-	-	-	-	-	-	-	-2.5	-0.9	0.7	-	-	-3.5	-4.0	-1.0	-0.9	-0.4	-	-0.3	-	no stat	-	-	-1.2
Lab 4	yes	-	-	-	-	-	-	-	-2.8	-0.8	1.5	-	-	-3.6	-4.0	-0.1	-0.7	-0.4	-	-0.2	-	no stat	-	-	-1.0
Lab 5	yes	-	-	-	-	-	-	-	-3.7	3.8	0.6	-	-	-1.5	-0.5	0.3	0.6	-	-	-	-	-	-	-	-0.3
Lab 5	yes	-	-	-	-	-	-	-	-1.5	2.5	1.9	-	-	-2.8	-	-0.4	1.3	-	-	-	-	-	-	-	0.2
Lab 6	no	-	-	no stat	-	-	-	-	0.7	-	-	no stat	-0.2	3.2	2.4	8.2	4.2	-	-	-	-	-	-	-	1.7
Lab 6	no	-	-	-	-	-	-	-	1.0	-	2.4	no stat	1.1	3.2	2.6	8.2	4.2	-	-	-	-	-	-	14.5	1.0
Lab 7	yes	-	-	-	-	-	-	-	-	-	4.3	-	-	-	-	1.2	2.0	-	-	-	-	-	-	-	-
Lab 7	yes	-	-	-	-	-	-	-	-	-	3.8	-	-	-	-	0.8	1.4	-	-	-	-	-	-	-	0.4
Lab 8	no	0.7	no stat	no stat	-	-	-	3.1	4.6	-	-3.5	-	10.9	1.0	3.9	0.1	-0.9	-	-	0.5	-	-	-	4.9	-1.3
Lab 8	no	0.9	no stat	no stat	-	-	-	3.0	5.2	-	-3.2	-	11.3	1.2	4.4	0.4	-0.5	-	-	-	-	-	-	4.5	-1.1
Lab 9	no	0.3	-	-	-	-	-	-0.1	-0.6	-	-1.1	-	-2.1	1.7	0.2	-	-	-	-	-	-	-	-	-	2.1
Lab 9	no	-0.3	-	-	-	-	-	0.3	0.1	-	-0.8	-	-2.3	1.8	0.7	-	-	-	-	-	-	-	-	-	2.8
Lab 10	no	0.3	-	-	-	-	-	0.4	-0.9	-	-1.2	-	-	1.5	-0.2	-	-	-	-	-	-	-	-	-1.5	0.7
Lab 10	no	-0.3	-	-	-	-	-	-0.1	-0.9	-	-0.9	-	-	1.6	-0.1	-	-	-	-	-	-	-	-	-1.6	0.9

Lab Code	ISTD	15-AcDON	3-AcDON	AFB1	AFB2	AFG1	AFG2	AOH	BEA	D3G	DON	ENN-A	ENN-A1	ENN-B	ENN-B1	FB1	FB2	FB3	HT-2	MON	NIV	OTA	OTB	T-2	ZEN
		Lot 7																							
Lab 1	yes	-	-	-	-	-	-	-	0.6	23.9	-	-	-	-	-0.2	-0.9	-0.4	-1.6	-	-0.8	-	-	-	-	0.2
Lab 1	yes	-	-	-	-	-	-	-	0.5	24.7	-	-	-	-	-0.2	-0.8	-0.2	-1.4	-	-0.9	-	-	-	-	0.0
Lab 2	no	-0.8	-	no stat	-	-	-	-3.2	-0.3	-2.5	-1.5	no stat	-0.9	-1.4	-0.2	1.2	1.3	0.7	no stat	0.9	no stat	-2.2	-	-0.2	-1.4
Lab 2	no	-1.2	-	no stat	-	-	-	-3.4	-0.9	-2.5	-1.8	no stat	-1.2	-1.7	-0.6	1.3	1.3	1.0	no stat	0.7	no stat	-2.3	-	-0.4	-0.9
Lab 3	yes	-	-	-	-	-	-	-	-	-	-0.8	-	-	-	-	-2.3	-2.0	-	no stat	-	-	-2.3	-	-0.9	1.1
Lab 3	yes	-	-	-	-	-	-	-	-	-	-0.5	-	-	-	-	-2.5	-2.3	-	no stat	-	-	-2.3	-	-0.6	0.1
Lab 4	yes	-	-	-	-	-	-	-	-2.3	-1.5	0.2	-	-	-3.2	-3.9	-0.1	-0.4	-0.4	-	-0.1	-	25.3	-	-	-1.2
Lab 4	yes	-	-	-	-	-	-	-	-2.7	-1.3	0.9	-	-	-3.2	-3.9	0.3	-0.9	0.2	-	-0.1	-	25.7	-	-	-1.2
Lab 5	yes	-	-	-	-	-	-	-	-2.3	0.5	-0.8	-	-	2.9	3.6	2.5	1.7	-	-	-	-	-	-	-	0.5
Lab 5	yes	-	-	-	-	-	-	-	-1.6	1.0	0.8	-	-	-2.2	-	0.1	1.9	1.1	-	-	-	-	-	-	1.1
Lab 6	no	-	-	-	-	-	-	-	-0.9	-	-1.5	no stat	-0.1	2.7	0.6	8.9	13.2	-	-	-	-	-	-	-	0.9
Lab 6	no	-	-	no stat	no stat	no stat	no stat	-	-1.4	-	-	no stat	1.3	2.4	1.1	9.7	4.3	-	-	-	-	-	no stat	14.6	1.1
Lab 7	yes	-	-	-	-	-	-	-	-	-	2.3	-	-	-	-	2.8	2.2	-	-	-	-	-	-	-	0.9
Lab 7	yes	-	-	-	-	-	-	-	-	-	1.8	-	-	-	-	2.4	2.1	-	-	-	-	-	-	-	1.0
Lab 8	no	1.5	-	-	-	-	-	7.6	2.9	-	-3.3	-	14.5	3.4	5.7	0.9	-0.8	-	-	0.3	-	15.6	-	3.9	-1.2
Lab 8	no	1.7	-	-	-	-	-	8.1	2.7	-	-3.3	-	14.4	3.1	5.3	1.1	-0.6	-	-	-	-	15.3	-	3.8	-1.3
Lab 9	no	-0.9	-	-	-	-	-	0.3	2.0	-	2.2	-	-2.1	-0.9	-0.1	-4.1	-2.5	-	-	-	-	-0.9	-	-	1.3
Lab 9	no	0.9	-	-	-	-	-	0.5	3.0	-	1.1	-	-1.5	-0.6	0.4	-3.9	-3.0	-	-	-	-	-0.6	-	-	0.9
Lab 10	no	-1.3	-	-	-	-	-	-1.6	0.2	-	2.8	-	-	-0.7	-0.8	-3.9	-2.0	-	-	-	-	-	-	-2.6	-1.0
Lab 10	no	0.1	-	-	-	-	-	-0.9	0.6	-	1.1	-	-	-0.6	-0.5	-3.9	-2.3	-	-	-	-	-	-	-1.6	-0.9

Lab Code	ISTD	15-AcDON	3-AcDON	AFB1	AFB2	AFG1	AFG2	AOH	BEA	D3G	DON	ENN-A	ENN-A1	ENN-B	ENN-B1	FB1	FB2	FB3	HT-2	MON	NIV	OTA	OTB	T-2	ZEN
		Lot 8																							
Lab 1	yes	-	-	-	-	-	-	-	0.1	-	2.4	-	-2.0	-	-1.1	-1.0	-2.2	-	-	-0.6	-	-1.6	-	-1.5	-0.2
Lab 1	yes	-	-	-	-	-	-	-	1.1	-	2.5	-	-	-	-0.7	-0.9	-2.3	-	-	-0.6	-	-1.5	-	-1.4	-0.2
Lab 2	no	1.1	-	no stat	-	-	-	-2.1	-1.2	-1.2	-1.5	no stat	-2.0	0.3	0.1	0.2	0.9	no stat	0.1	0.4	-	-2.2	-	-1.5	-2.3
Lab 2	no	2.7	-	no stat	-	-	-	-2.2	-1.1	-1.4	-0.6	no stat	-2.1	-0.4	-0.5	1.0	2.0	no stat	0.4	1.0	-	-2.3	-	-0.9	-2.3
Lab 3	yes	-	-	no stat	-	-	-	-	-	-	-0.9	-	-	-	-	-2.5	-3.4	-	-0.1	-	-	-1.2	-	-0.4	-0.1
Lab 3	yes	-	-	no stat	-	-	-	-	-	-	-1.1	-	-	-	-	-2.3	-3.3	-	0.1	-	-	-3.2	-	-1.4	-0.1
Lab 4	yes	-	-	-	-	-	-	-	1.5	-0.3	1.4	-	-	-1.4	-3.1	-1.0	-2.4	-	-	0.2	-	16.1	-	0.8	0.5
Lab 4	yes	-	-	-	-	-	-	-	-1.2	-0.2	0.8	-	-	-1.5	-3.3	-0.5	-2.7	-	-	0.3	-	14.9	-	0.7	0.7
Lab 5	yes	11.5	-	-	-	-	-	-	-3.6	1.3	2.9	-	-	0.8	0.1	2.9	-1.1	-	-	-	-	-1.8	-	-	0.7
Lab 5	yes	-	-	-	-	-	-	-	-2.8	1.8	0.6	-	-	-0.3	0.2	0.6	-0.5	-	-	-	-	-	-	-	2.1
Lab 6	no	-	-	-	-	-	-	-	-0.6	-	-1.4	no stat	-0.6	5.2	0.4	29.6	24.8	-	-	-	-	1.8	-	1.0	0.4
Lab 6	no	-	-	-	-	-	-	-	-1.4	-	1.4	no stat	2.0	7.5	2.3	26.1	11.1	-	22.7	-	-	-	-	6.2	1.1
Lab 7	yes	-	-	-	-	-	-	-	-	-	3.1	-	-	-	-	0.3	4.0	-	-	-	-	-	-	-	0.5
Lab 7	yes	-	-	-	-	-	-	-	-	-	2.8	-	-	-	-	1.2	5.9	-	-	-	-	-	-	-	0.5
Lab 8	no	0.0	no stat	no stat	-	-	-	25.1	2.2	-	-3.8	-	17.4	3.3	4.1	2.3	0.4	-	-1.1	-0.8	-	5.2	-	4.5	-1.9
Lab 8	no	0.7	no stat	no stat	-	-	-	25.3	2.3	-	-3.7	-	17.5	4.0	4.5	0.3	-0.1	-	-1.6	-	-	4.9	-	0.0	-2.0
Lab 9	no	-1.2	-	-	-	-	-	-1.0	2.1	-	-0.2	-	-1.3	-1.5	1.1	-0.4	-0.7	-	-	-	-	-	-	-	1.1
Lab 9	no	-1.8	-	-	-	-	-	1.4	1.5	-	-1.5	-	-0.6	-1.7	-0.1	-0.7	0.8	-	-	-	-	-	-	-	1.0
Lab 10	no	-2.5	-	-	-	-	-	-1.9	-0.2	-	-0.6	-	-	-1.7	-0.5	-1.0	-0.1	-	-	-	-	-	-	-	-1.1
Lab 10	no	-2.5	-	-	-	-	-	0.0	0.4	-	-1.9	-	-	-1.8	-0.9	-1.5	-0.7	-	-	-	-	-	-	-	-1.0

Lab Code	ISTD	15-AcDON	3-AcDON	AFB1	AFB2	AFG1	AFG2	AOH	BEA	D3G	DON	ENN-A	ENN-A1	ENN-B	ENN-B1	FB1	FB2	FB3	HT-2	MON	NIV	OTA	OTB	T-2	ZEN
		Lot 9																							
Lab 1	yes	-	-	-	-	-	-	-	-0.4	-	2.4	-	-	-	-0.9	-1.2	-2.2	-	-	-0.9	-	0.7	-	-1.5	0.6
Lab 1	yes	-	-	-	-	-	-	-	0.1	-	2.9	-	-	-	-0.5	-0.9	-2.2	-	-	-0.8	-	1.0	-	-1.3	0.8
Lab 2	no	2.3	-	no stat	-	-	-	-3.3	-1.6	-1.6	-1.2	no stat	-1.4	-0.8	0.6	1.1	1.3	no stat	0.3	0.2	-	-0.9	-	-0.5	-2.3
Lab 2	no	0.8	-	no stat	-	-	-	-3.1	-1.6	-1.6	-1.1	no stat	-1.3	-0.7	0.7	0.7	0.6	no stat	1.6	-0.2	-	-1.2	-	1.1	-2.1
Lab 3	yes	-	-	no stat	-	-	-	-	-	-	-1.6	-	-	-	-	-2.3	-3.4	-	0.6	-	-	-1.9	-	-0.2	-0.2
Lab 3	yes	-	-	no stat	-	-	-	-	-	-	-0.3	-	-	-	-	-2.1	-3.4	-	0.6	-	-	-1.5	-	-0.8	5.1
Lab 4	yes	-	-	-	-	-	-	-	0.5	4.6	0.8	-	-	-2.3	-3.1	-1.1	-2.5	-	-	0.1	-	-	-	2.2	-0.8
Lab 4	yes	-	-	-	-	-	-	-	1.6	4.6	0.6	-	-	-2.3	-3.0	-1.7	-2.9	-	-	0.3	-	-	-	1.9	-0.8
Lab 5	yes	-	-	-	-	-	-	-	-3.2	-0.9	-0.6	-	-	0.4	1.5	1.0	-1.3	-	-	-	-	1.3	-	-	0.6
Lab 5	yes	-	-	-	-	-	-	-	-2.4	0.1	-	-	-	-1.1	-1.2	1.0	-0.4	-	-	-	-	3.3	-	-	2.0
Lab 6	no	-	-	-	-	-	-	-	-1.1	-	-1.9	no stat	-0.1	3.8	1.8	20.2	19.4	-	-	-	-	-	-	1.6	4.7
Lab 6	no	-	-	-	-	-	-	-	-1.7	-	0.3	no stat	1.5	5.9	3.6	27.4	12.0	-	-	-	-	-	-	5.5	1.2
Lab 7	yes	-	-	-	-	-	-	-	-	-	-0.2	-	-	-	-	2.6	5.1	-	-	-	-	-	-	-	0.0
Lab 7	yes	-	-	-	-	-	-	-	-	-	2.5	-	-	-	-	6.9	9.0	-	-	-	-	-	-	-	0.8
Lab 8	no	-0.1	no stat	no stat	-	-	-	19.7	3.0	-	-3.6	-	14.4	1.4	4.3	4.8	1.2	-	-0.4	0.8	-	12.1	-	0.7	-1.8
Lab 8	no	-0.4	no stat	no stat	-	-	-	20.0	1.8	-	-3.6	-	14.5	1.6	4.4	0.5	-0.3	-	-0.2	-	-	12.7	-	0.8	-1.6
Lab 9	no	0.0	-	-	-	-	-	-2.2	1.8	-	0.6	-	-2.8	0.5	0.3	-0.3	-	-	-	-	-	-1.3	-	0.3	0.8
Lab 9	no	0.8	-	-	-	-	-	-2.5	1.2	-	1.4	-	-2.7	0.9	0.8	0.2	-	-	-	-	-	-0.8	-	0.5	1.2
Lab 10	no	0.4	-	-	-	-	-	-1.2	-0.1	-	1.5	-	-	-0.1	-0.7	-0.9	-	-	-	-	-	-	-	-1.8	-1.0
Lab 10	no	0.1	-	-	-	-	-	-1.3	0.2	-	0.7	-	-	0.1	-0.2	-0.8	-	-	-	-	-	-	-	-1.5	-0.9

Lab Code	ISTD	15-AcDON	3-AcDON	AFB1	AFB2	AFG1	AFG2	AOH	BEA	D3G	DON	ENN-A	ENN-A1	ENN-B	ENN-B1	FB1	FB2	FB3	HT-2	MON	NIV	OTA	OTB	T-2	ZEN
		Lot 10																							
Lab 1	yes	-	-	-	-	-	-	-	-0.4	-	-	-	-	-	-	-1.0	-2.5	-	-	-0.9	-	-	-	-	-0.2
Lab 1	yes	-	-	-	-	-	-	-	-1.2	-	-	-	-	-	-	-1.1	-2.4	-	-	-1.0	-	-	-	-	-0.5
Lab 2	no	-	-	no stat	-	-	-	-4.5	-1.4	-	0.9	no stat	-1.0	-1.9	-1.2	0.3	0.7	no stat	-	0.6	-	-1.7	-	no stat	-3.4
Lab 2	no	-	-	no stat	-	-	-	-4.5	-0.8	-	-1.0	no stat	-0.9	-1.3	-0.7	0.0	0.3	no stat	-	0.3	-	-1.5	-	no stat	-3.4
Lab 3	yes	-	-	-	-	-	-	-	-	-	1.5	-	-	-	-	-2.4	-3.3	-	no stat	-	-	-0.9	-	no stat	2.8
Lab 3	yes	-	-	-	-	-	-	-	-	-	1.2	-	-	-	-	-2.5	-3.4	-	no stat	-	-	-1.7	-	no stat	3.3
Lab 4	yes	-	-	-	-	-	-	-	-0.1	-	-	-	-	-1.9	-3.3	-0.6	-3.0	-	-	0.8	-	-	-	-	-1.3
Lab 4	yes	-	-	-	-	-	-	-	-0.9	-	-	-	-	-1.9	-3.2	-1.0	-2.7	-	-	0.8	-	-	-	-	-1.0
Lab 5	yes	-	-	-	-	-	-	-	-2.7	-	-	-	-	-0.4	0.1	0.2	-1.6	-	-	-	-	-	-	-	-
Lab 5	yes	-	-	-	-	-	-	-	-2.9	-	-	-	-	-1.9	-	0.8	-0.3	-	-	-	-	-	-	-	-
Lab 6	no	-	-	-	-	-	-	-	-0.5	-	-	no stat	0.1	1.1	-0.6	12.5	13.9	-	-	-	-	-	-	-	-
Lab 6	no	-	-	-	-	-	-	-	-1.0	-	-	no stat	1.8	1.8	0.5	24.2	11.4	-	-	-	-	-	-	-	5.8
Lab 7	yes	-	-	-	-	-	-	-	-	-	-	-	-	-	-	0.7	4.5	-	-	-	-	-	-	-	-
Lab 7	yes	-	-	-	-	-	-	-	-	-	-	-	-	-	-	1.5	5.3	-	-	-	-	-	-	-	-
Lab 8	no	no stat	no stat	-	-	-	-	-2.3	4.6	-	-0.4	-	21.3	5.7	7.4	1.0	-0.3	-	-	-1.3	-	25.4	-	-	0.5
Lab 8	no	no stat	no stat	-	-	-	-	-2.3	8.8	-	-0.7	-	18.0	2.5	3.9	0.7	0.1	-	-	-	-	25.1	-	-	0.2
Lab 9	no	-	-	-	-	-	-	-	4.8	-	-	-	-0.7	0.7	1.0	0.4	-	-	-	-	-	0.9	-	-	1.4
Lab 9	no	-	-	-	-	-	-	-	4.0	-	-	-	-0.7	0.8	1.3	-1.4	-	-	-	-	-	0.2	-	-	1.2
Lab 10	no	-	-	-	-	-	-	-	1.2	-	-	-	-	-0.3	0.0	-0.3	-	-	-	-	-	-	-	-	-0.7
Lab 10	no	-	-	-	-	-	-	-	1.4	-	-	-	-	-0.3	-0.6	-0.4	-	-	-	-	-	-	-	-	-1.1

3.4. Overview of individual z-score data for swine feed matrix

Table S9: Summary of z-score performance of 10 swine feed matrices. Acceptable, questionable and unacceptable z-scores are colored in green, yellow and red respectively. No-stat information refers to positivefindings where a z-score calculation was not feasible due to a reduced number of reported results.

Lab Code	ISTD	15-AcDON	3-AcDON	AFB1	AFB2	AFG1	AFG2	AOH	BEA	D3G	DON	ENN-A	ENN-A1	ENN-B	ENN-B1	FB1	FB2	FB3	HT-2	MON	NIV	OTA	OTB	T-2	ZEN
		Lot 1																							
Lab 1	yes	-	-	-	-	-	-	-	-	-	-	-	-	-	-	1.0	-0.7	-	-	0.8	-	2.6	-	-	-
Lab 1	yes	-	-	-	-	-	-	-	-0.8	-	-	-	-	-	-	0.7	-0.9	-	-	1.2	-	2.8	-	-	-
Lab 2	no	-	-	no stat	-	-	-	-2.1	0.0	-	-0.4	-0.2	0.2	-0.3	0.3	0.4	1.6	no stat	-	-0.4	-	-0.3	no stat	-1.8	-3.0
Lab 2	no	-	-	no stat	-	-	-	1.5	2.2	-	-0.5	-0.1	0.9	0.6	1.2	-0.6	1.0	no stat	-	-0.5	-	-0.4	no stat	0.4	-2.9
Lab 3	yes	-	-	no stat	-	-	-	-	-	-	1.6	-	-	-	-	-2.7	-3.2	-	no stat	-	-	-1.4	-	2.1	2.2
Lab 3	yes	-	-	no stat	-	-	-	-	-	-	-1.5	-	-	-	-	-2.7	-3.0	-	no stat	-	-	-1.2	-	-0.4	0.9
Lab 4	yes	-	-	-	-	-	-	-	4.2	-	-	-	-	-1.8	-3.2	0.9	-1.3	no stat	-	-0.1	-	6.5	-	12.0	0.1
Lab 4	yes	-	-	-	-	-	-	-	1.8	-	-	-	-	-2.3	-3.3	0.4	-0.9	no stat	-	-0.3	-	5.9	-	9.3	0.5
Lab 5	yes	-	-	-	-	-	-	-	-3.9	-	-	-	-	-2.0	-	-0.5	-1.2	-	-	-	-	0.9	-	-	-
Lab 5	yes	-	-	-	-	-	-	-	-3.6	-	-	-	-	-1.1	-	-0.2	-0.5	-	-	-	-	-0.1	-	-	-
Lab 6	no	-	-	-	-	-	-	-	0.6	-	-	0.2	0.8	6.6	1.4	9.3	19.8	-	-	-	-	0.1	-	-	-
Lab 6	no	-	-	-	-	-	-	-	0.6	-	-	1.5	2.2	4.7	1.2	9.3	29.3	-	-	-	-	0.1	-	-	10.0
Lab 7	yes	-	-	-	-	-	-	-	-	-	7.1	-	-	-	-	0.8	3.0	-	-	-	-	1.6	-	-	-
Lab 7	yes	-	-	-	-	-	-	-	-	-	10.3	-	-	-	-	3.2	3.5	-	-	-	-	0.5	-	-	-
Lab 8	no	no stat	no stat	-	-	-	-	-	-0.9	-	-3.9	-	-	-	-	-1.3	-2.1	-	no stat	-1.8	-	-1.3	no stat	-	-
Lab 8	no	no stat	no stat	-	-	-	-	-	-0.8	-	-4.3	-	-4.5	-	-	-0.3	-1.7	-	no stat	0.9	-	-1.3	no stat	-2.6	-
Lab 9	no	-	-	-	-	-	-	1.7	-2.4	-	-	-1.2	-1.8	-1.2	-0.9	-0.7	-	-	-	-	-	-2.0	-	-	0.3
Lab 9	no	-	-	-	-	-	-	1.1	0.8	-	-	0.1	-0.7	-0.1	-0.8	-1.0	-	-	-	-	-	-1.4	-	-	0.0
Lab 10	no	-	-	-	-	-	-	-0.4	0.1	-	-	-	-	1.3	0.8	-0.5	-	-	-	-	-	-1.4	-	-0.7	-1.3
Lab 10	no	-	-	-	-	-	-	-1.8	0.1	-	-	-	-	1.2	0.3	-0.2	-	-	-	-	-	-1.4	-	-1.7	-0.9

Lab Code	ISTD	15-AcDON	3-AcDON	AFB1	AFB2	AFG1	AFG2	AOH	BEA	D3G	DON	ENN-A	ENN-A1	ENN-B	ENN-B1	FB1	FB2	FB3	HT-2	MON	NIV	OTA	OTB	T-2	ZEN
		Lot 2																							
Lab 1	yes	-	-	-	-	-	-	-	-1.5	no stat	-	-	-1.1	-2.9	-1.5	0.0	-	-	-	3.1	-	-	-	-1.0	-0.5
Lab 1	yes	-	-	-	-	-	-	-	-1.9	-	-	-	-1.2	-2.8	-1.5	-0.3	-	-	-	3.3	-	-	-	-1.0	-0.6
Lab 2	no	no stat	-	-	-	-	-	-2.4	-0.3	no stat	-2.0	-0.4	1.0	-0.7	0.6	2.0	-0.1	-	1.1	-0.3	2.4	-	-	0.5	-1.8
Lab 2	no	no stat	-	-	-	-	-	-2.2	1.0	no stat	-2.1	0.6	1.8	-0.8	0.9	1.8	0.5	-	1.1	-1.0	2.1	-	-	1.0	-1.2
Lab 3	yes	-	-	-	-	-	-	-	-	-	-1.6	-	-	-	-	-2.4	-3.4	-	-0.2	-	-	no stat	-	-1.4	-1.0
Lab 3	yes	-	-	-	-	-	-	-	-	-	-1.2	-	-	-	-	-2.6	-3.6	-	0.0	-	-	no stat	-	-2.2	-1.3
Lab 4	yes	-	-	-	-	-	-	-2.0	-0.1	no stat	0.2	-2.1	-3.0	-0.7	-2.3	-	-	-	-	0.0	-0.1	-	-	0.7	1.2
Lab 4	yes	-	-	-	-	-	-	-1.7	0.2	no stat	0.6	-2.1	-2.9	-0.7	-2.2	-	-	-	-	0.0	0.4	-	-	2.6	2.0
Lab 5	yes	-	-	-	-	-	-	3.8	-3.2	-	-0.2	-	1.5	1.3	2.9	-	-	-	-	-	1.2	-	-	-	-
Lab 5	yes	-	-	-	-	-	-	4.6	-2.1	-	0.1	-	-1.0	-0.1	-0.1	-	-	-	-	-	1.0	-	-	-	-
Lab 6	no	-	-	-	-	-	-	-	-0.3	-	0.1	0.1	2.0	3.9	1.3	-	51.4	-	-	-	-	-	-	-0.3	1.2
Lab 6	no	-	-	-	-	-	-	-	-0.2	-	-0.2	0.6	2.3	1.8	0.5	-	44.6	-	-	-	-	-	-	-	2.3
Lab 7	yes	-	-	-	-	-	-	-	-	-	0.8	-	-	-	-	-	-	-	-	-	-	-	-	-	-
Lab 7	yes	-	-	-	-	-	-	-	-	-	1.3	-	-	-	-	-	-	-	-	-	-	-	-	-	-
Lab 8	no	no stat	no stat	-	-	-	-	-1.9	0.3	-	-3.9	-	-1.3	-0.3	-0.4	0.2	-0.7	-	-1.8	-2.4	-1.8	-	-	-2.5	-3.5
Lab 8	no	no stat	no stat	-	-	-	-	-0.4	1.1	-	-3.9	-	-0.9	0.3	0.2	1.4	-0.3	-	3.4	-2.3	-2.4	-	-	9.2	-3.1
Lab 9	no	-	-	-	-	-	-	0.7	0.3	-	3.2	0.1	0.9	-0.1	-0.2	-	-	-	-	-	-	-	-	1.8	0.8
Lab 9	no	-	-	-	-	-	-	0.4	0.4	-	3.0	0.2	0.9	-0.1	-0.1	-	-	-	-	-	-	-	-	0.9	1.4
Lab 10	no	-	-	-	-	-	-	1.1	0.4	-	2.0	0.1	-0.1	1.2	1.0	-	-	-	-1.0	-	-1.2	-	-	-0.5	1.1
Lab 10	no	-	-	-	-	-	-	0.9	1.6	-	2.2	0.6	0.4	1.2	0.8	-	-	-	-1.7	-	-1.6	-	-	-0.3	1.3

Lab Code	ISTD	15-AcDON	3-AcDON	AFB1	AFB2	AFG1	AFG2	AOH	BEA	D3G	DON	ENN-A	ENN-A1	ENN-B	ENN-B1	FB1	FB2	FB3	HT-2	MON	NIV	OTA	OTB	T-2	ZEN
		Lot 3																							
Lab 1	yes	-	-	-	-	-	-	0.4	-0.8	-	-	-	-1.7	-2.8	-1.6	0.4	-	-	-	3.5	-	-	-	-0.9	-0.3
Lab 1	yes	-	-	-	-	-	-	0.3	1.0	-	-	-	0.1	-2.6	-1.2	1.2	-	-	-	3.3	-	-	-	-0.5	-0.4
Lab 2	no	-0.7	-	-	-	-	-	-2.2	0.0	no stat	-1.7	1.6	2.9	0.1	2.1	-0.1	-0.1	-	1.7	-0.4	1.1	-	-	0.8	-1.7
Lab 2	no	-0.8	-	-	-	-	-	-2.1	2.6	no stat	-1.5	0.4	1.4	0.2	1.2	-0.8	-1.1	-	1.6	-0.3	2.8	-	-	1.7	-1.6
Lab 3	yes	-	-	-	-	-	-	-	-	-	-1.0	-	-	-	-	-3.0	-3.2	-	1.2	-	-	-	-	-1.4	-1.1
Lab 3	yes	-	-	-	-	-	-	-	-	-	-1.3	-	-	-	-	-3.0	-3.1	-	-0.6	-	-	-	-	-1.6	0.7
Lab 4	yes	-	-	-	-	-	-	-1.3	-0.6	no stat	0.5	-2.8	-3.5	-1.4	-2.9	-	-	-	-	0.3	0.7	-	-	1.4	1.7
Lab 4	yes	-	-	-	-	-	-	-1.3	-1.8	no stat	0.6	-3.2	-3.7	-1.6	-3.0	-	-	-	-	0.1	0.5	-	-	3.2	1.8
Lab 5	yes	-	-	-	-	-	-	3.0	-3.3	-	0.2	0.0	1.2	0.5	2.0	0.0	-	-	-	-	0.6	-	-	-	-
Lab 5	yes	-	-	-	-	-	-	2.7	-2.4	-	-0.2	-	-1.7	-0.4	-0.6	-	-	-	-	-	1.2	-	-	-	-
Lab 6	no	-	-	-	-	-	-	-	2.1	-	-	0.8	2.8	4.8	2.4	12.2	34.9	-	-	-	-	-	-	1.4	1.5
Lab 6	no	-	-	-	-	-	-	-	0.4	-	0.5	1.2	2.6	4.2	1.3	-	40.0	-	-	-	-	-	-	-	1.0
Lab 7	yes	-	-	-	-	-	-	-	-	-	0.6	-	-	-	-	-	-	-	-	-	-	-	-	-	-
Lab 7	yes	-	-	-	-	-	-	-	-	-	0.9	-	-	-	-	-	-	-	-	-	-	-	-	-	-
Lab 8	no	-3.0	no stat	-	-	-	-	-1.7	0.1	-	-3.5	-	-1.3	0.4	-0.1	1.6	0.8	-	-2.0	-1.9	-1.7	-	-	-2.5	-3.1
Lab 8	no	-2.8	no stat	-	-	-	-	-2.8	0.9	-	-3.6	-	-1.1	0.4	0.1	-0.1	-0.7	-	-1.9	-1.7	-2.6	-	-	-2.1	-3.2
Lab 9	no	-	-	-	-	-	-	1.9	-0.4	-	1.0	-0.7	0.0	-0.3	-0.8	-	-	-	-	-	-	-	-	1.5	0.9
Lab 9	no	-	-	-	-	-	-	0.9	-0.8	-	0.5	-0.6	-0.1	-0.5	-0.8	-	-	-	-	-	-	-	-	1.2	1.1
Lab 10	no	5.0	-	-	-	-	-	0.8	1.0	-	0.6	0.3	0.1	1.0	0.5	-	-	-	-	-	-1.4	-	-	-1.2	0.2
Lab 10	no	4.9	-	-	-	-	-	1.2	0.8	-	0.9	0.3	0.5	1.0	0.8	-	-	-	-	-	-1.5	-	-	-0.6	0.2

Lab Code	ISTD	15-AcDON	3-AcDON	AFB1	AFB2	AFG1	AFG2	AOH	BEA	D3G	DON	ENN-A	ENN-A1	ENN-B	ENN-B1	FB1	FB2	FB3	HT-2	MON	NIV	OTA	OTB	T-2	ZEN
		Lot 4																							
Lab 1	yes	-	-	-	-	-	-	-	-	12.4	2.0	-	-1.3	-3.0	-1.7	-0.3	-	-	-	5.4	-	-	-	-	0.1
Lab 1	yes	-	-	-	-	-	-	-	-	-	1.8	-	-1.0	-3.0	-1.7	-0.2	-	-	-	5.4	-	-	-	-	-0.2
Lab 2	no	no stat	-	-	-	-	-	-3.6	-0.4	-0.3	-1.7	0.7	1.7	-0.2	1.0	0.2	0.3	-	0.8	-1.2	no stat	-0.8	-	0.6	-0.9
Lab 2	no	no stat	-	-	-	-	-	-3.6	0.6	-0.3	-2.0	1.1	2.2	-0.6	0.9	0.1	1.0	-	1.3	-0.7	no stat	2.4	-	0.4	-1.4
Lab 3	yes	-	-	-	-	-	-	-	-	-	-1.3	-	-	-	-	-3.1	-2.7	-	-0.2	-	-	-1.6	-	0.9	-0.3
Lab 3	yes	-	-	-	-	-	-	-	-	-	-1.2	-	-	-	-	-3.0	-3.0	-	1.2	-	-	-1.1	-	1.3	-0.6
Lab 4	yes	-	-	-	-	-	-	-	1.5	1.2	0.4	-1.8	-3.0	-1.2	-2.5	-0.1	0.6	-	-	-0.5	-	-	-	-	0.4
Lab 4	yes	-	-	-	-	-	-	-	-1.0	1.1	0.3	-2.7	-3.3	-1.5	-2.7	0.0	-0.4	-	-	-0.4	-	-	-	-	0.5
Lab 5	yes	-	-	-	-	-	-	-0.5	-2.6	0.7	0.4	0.3	2.2	0.7	2.8	0.4	-	-	-	-	-	1.3	-	-	1.9
Lab 5	yes	-	-	-	-	-	-	-	-2.2	1.0	0.9	-	-1.2	0.2	-0.1	-0.5	-	-	-	-	-	-	-	-	1.3
Lab 6	no	-	-	-	-	-	-	-	0.3	-	-0.9	0.0	1.6	3.7	1.8	14.2	28.2	-	-	-	-	-	-	10.0	0.8
Lab 6	no	-	-	-	-	-	-	-	0.2	-	-0.5	-0.4	1.5	1.9	0.2	11.2	26.0	-	-	-	-	-	-	-	1.5
Lab 7	yes	-	-	-	-	-	-	-	-	-	0.6	-	-	-	-	1.2	-	-	-	-	-	-	-	-	0.0
Lab 7	yes	-	-	-	-	-	-	-	-	-	1.8	-	-	-	-	4.4	-	-	-	-	-	-	-	-	0.8
Lab 8	no	no stat	-	-	-	-	-	-3.8	0.2	-	-4.0	-	-0.4	2.1	1.3	-0.7	-0.8	-	-2.8	-2.4	-	-0.6	-	-1.6	-2.6
Lab 8	no	no stat	-	-	-	-	-	-3.0	-0.6	-	-4.0	-	-1.1	0.2	-0.1	-0.5	-0.5	-	-2.2	1.1	-	7.4	-	-2.6	-2.8
Lab 9	no	-	-	-	-	-	-	3.2	-0.2	-1.6	-1.2	-0.1	0.4	-0.1	-0.6	-	-	-	-	-	-	-	-	-	0.0
Lab 9	no	-	-	-	-	-	-	3.3	0.9	-1.1	2.6	-0.2	0.8	-0.3	-0.5	-	-	-	-	-	-	-	-	-	-0.1
Lab 10	no	-	no stat	-	-	-	-	4.1	0.9	-1.4	1.9	0.2	0.2	0.5	0.8	-	-	-	-	-	-	-	-	-1.5	0.0
Lab 10	no	-	no stat	-	-	-	-	3.9	0.3	-1.3	2.3	0.3	-0.1	0.6	0.7	-	-	-	-	-	-	-	-	-2.0	-0.3

Lab Code	ISTD	15-AcDON	3-AcDON	AFB1	AFB2	AFG1	AFG2	AOH	BEA	D3G	DON	ENN-A	ENN-A1	ENN-B	ENN-B1	FB1	FB2	FB3	HT-2	MON	NIV	OTA	OTB	T-2	ZEN
		Lot 5																							
Lab 1	yes	-	-	-	-	-	-	-	-	-	2.1	-	-1.9	-2.8	-1.8	1.5	-	-	-	2.0	-	-	-	-	-0.5
Lab 1	yes	-	-	-	-	-	-	-	-0.9	-	2.7	-	-1.3	-2.8	-1.8	2.2	0.9	-	-	2.7	-	-	-	-	-0.1
Lab 2	no	-1.1	0.1	no stat	-	-	-	-2.8	0.3	-0.5	-2.7	0.1	1.2	0.3	1.0	-0.8	0.6	-	6.5	-0.4	no stat	-	-	no stat	-1.0
Lab 2	no	-1.0	-0.8	no stat	-	-	-	-1.8	0.0	-0.5	-3.0	0.4	1.7	-0.2	1.0	1.7	0.9	-	4.5	-0.6	no stat	-	-	no stat	-1.5
Lab 3	yes	-	-	-	-	-	-	-	-	-	-2.1	-	-	-	-	-2.2	-2.0	-	0.1	-	-	no stat	-	no stat	-0.2
Lab 3	yes	-	-	-	-	-	-	-	-	-	-2.1	-	-	-	-	-2.1	-2.0	-	-0.3	-	-	no stat	-	no stat	-1.0
Lab 4	yes	4.6	-	-	-	-	-	-0.3	-2.5	1.0	0.0	-3.7	-4.0	-2.6	-3.7	0.5	-1.1	-	-	0.3	-	-	-	-	-0.5
Lab 4	yes	5.0	-	-	-	-	-	0.5	-2.3	0.5	0.9	-3.5	-3.9	-2.4	-3.6	-0.6	-1.1	-	-	0.4	-	-	-	-	-0.8
Lab 5	yes	5.0	-	-	-	-	-	-	-1.7	1.0	0.0	0.8	2.4	1.3	3.0	1.5	-	-	-	-	-	-	-	-	4.4
Lab 5	yes	4.5	-	-	-	-	-	4.4	-1.7	0.8	0.1	0.8	1.7	1.4	1.8	1.1	-	-	-	-	-	-	-	-	1.7
Lab 6	no	-	-	-	-	-	-	-	3.3	-	-1.5	0.2	2.0	6.1	2.5	13.3	26.9	-	-	-	-	-	-	-	2.8
Lab 6	no	-	-	-	-	-	-	-	3.9	-	-1.0	0.7	2.1	3.5	0.9	11.5	29.3	-	-	-	-	-	-	-	1.7
Lab 7	yes	-	-	-	-	-	-	-	-	-	0.6	-	-	-	-	-0.8	-	-	-	-	-	-	-	-	1.0
Lab 7	yes	-	-	-	-	-	-	-	-	-	1.0	-	-	-	-	-0.5	-	-	-	-	-	-	-	-	0.8
Lab 8	no	-2.6	-2.9	-	-	-	no stat	-3.9	1.3	-	-4.2	-	-1.4	0.1	-0.5	-0.8	-0.4	-	-0.3	-2.1	-	no stat	-	no stat	-3.0
Lab 8	no	-2.5	-3.0	-	-	-	no stat	-4.2	1.0	-	-4.2	-	-1.3	0.1	-0.5	-0.8	-0.5	-	-0.5	-2.1	-	no stat	-	-	-3.0
Lab 9	no	0.2	1.2	-	-	-	-	1.6	0.0	-0.1	3.5	-0.5	0.3	-0.2	-0.7	-1.3	-	-	-	-	-	-	-	-	0.6
Lab 9	no	-1.0	2.3	-	-	-	-	2.6	-0.5	-0.6	2.9	-0.6	0.2	-0.5	-1.0	-1.6	-	-	-	-	-	-	-	-	0.7
Lab 10	no	-0.6	0.8	-	-	-	-	1.6	1.2	-0.7	3.1	0.2	0.3	1.0	0.7	-0.7	-	-	-	-	-	-	-	-	-0.1
Lab 10	no	-0.6	0.2	-	-	-	-	2.1	0.7	-0.3	4.0	0.1	0.1	0.9	0.7	-0.9	-	-	-	-	-	-	-	-	-0.2

Lab Code	ISTD	15-AcDON	3-AcDON	AFB1	AFB2	AFG1	AFG2	AOH	BEA	D3G	DON	ENN-A	ENN-A1	ENN-B	ENN-B1	FB1	FB2	FB3	HT-2	MON	NIV	OTA	OTB	T-2	ZEN
		Lot 6																							
Lab 1	yes	-	-	-	-	-	-	-	-1.4	-	2.7	-	-1.8	-2.3	-0.9	0.8	-0.6	-	-	3.5	-	3.4	-	-	-0.2
Lab 1	yes	-	-	-	-	-	-	-	-1.2	-	3.4	-	-1.9	-2.3	-0.7	1.0	-0.4	-	-	3.7	-	3.7	-	-	0.1
Lab 2	no	0.0	-0.5	-	-	-	-	-1.4	0.8	1.0	-1.8	0.7	0.8	-0.5	0.9	-0.4	1.2	no stat	-	-1.1	-0.1	-1.5	-	-1.1	-0.8
Lab 2	no	-0.9	-1.4	-	-	-	-	-2.6	0.6	1.3	-2.3	0.2	1.0	0.1	1.5	-0.8	0.6	no stat	-	-1.0	1.9	-1.7	-	4.5	-2.0
Lab 3	yes	-	-	-	-	-	-	-	-	-	-1.3	-	-	-	-	-2.7	-2.8	-	0.6	-	-	-1.0	-	-2.3	1.0
Lab 3	yes	-	-	-	-	-	-	-	-	-	-1.2	-	-	-	-	-3.3	-3.1	-	0.7	-	-	-1.5	-	-0.8	2.5
Lab 4	yes	5.5	23.3	-	-	-	-	-	-1.7	-0.1	0.7	-3.3	-4.1	-2.9	-3.8	1.0	-1.6	no stat	-	0.7	0.5	17.3	-	1.4	0.1
Lab 4	yes	5.6	19.3	-	-	-	-	-	-1.0	-0.2	0.4	-3.0	-4.0	-2.7	-3.6	0.8	-1.1	no stat	-	0.7	0.3	19.4	-	1.6	0.5
Lab 5	yes	8.1	-	-	-	-	-	321.6	-4.2	17.7	-3.0	-	-	-1.6	-2.6	0.8	0.2	-	-	-	-	0.4	-	-	0.4
Lab 5	yes	4.2	-	-	-	-	-	-	-3.6	0.0	0.3	-	-	-2.5	-3.4	-0.4	0.2	-	-	-	-	-1.3	-	-	1.3
Lab 6	no	-	-	-	-	-	-	-	1.7	-	-1.2	0.9	1.1	5.8	3.3	10.4	8.6	-	-	-	-	0.5	-	-	1.5
Lab 6	no	-	-	-	-	-	-	-	1.0	-	-0.9	2.1	3.3	6.7	3.6	8.9	12.1	-	-	-	-	-0.5	no stat	-	-0.5
Lab 7	yes	-	-	-	-	-	-	-	-	-	1.4	-	-	-	-	4.3	6.7	-	-	-	-	4.4	-	-	1.2
Lab 7	yes	-	-	-	-	-	-	-	-	-	2.9	-	-	-	-	2.8	1.8	-	-	-	-	-1.4	-	-	0.1
Lab 8	no	-3.2	-3.4	-	-	-	no stat	-4.3	2.0	-	-4.6	-	-0.6	4.1	2.1	-0.5	-1.2	-	0.3	-1.9	-2.2	-0.8	no stat	-2.9	-3.3
Lab 8	no	-2.5	-3.3	-	-	-	no stat	-1.4	2.4	-	-4.3	-	-1.0	0.9	0.3	-0.9	-1.3	-	0.1	-2.5	-1.0	7.6	no stat	-3.4	-3.5
Lab 9	no	-0.7	2.2	-	-	-	-	0.3	-0.1	-0.2	1.6	-0.1	0.4	-0.1	-0.2	-1.1	-	-	-	-	-	-2.7	-	1.3	-0.2
Lab 9	no	-0.3	0.8	-	-	-	-	0.7	-0.3	0.1	1.8	-0.2	0.3	0.1	-0.4	-1.7	-	-	-	-	-	-3.1	-	1.8	-0.1
Lab 10	no	-0.9	-0.9	-	-	-	-	1.8	1.4	-0.8	1.6	0.1	0.7	0.9	1.5	-1.0	-	-	-2.9	-	-	-2.6	-	0.6	-0.7
Lab 10	no	-0.6	-0.9	-	-	-	-	1.9	1.8	-0.2	1.0	-0.4	1.2	0.9	1.7	-0.8	-	-	-1.5	-	-	-2.9	-	-0.2	-0.7

Lot 7

Lab Code	ISTD	15-AcDON	3-AcDON	AFB1	AFB2	AFG1	AFG2	AOH	BEA	D3G	DON	ENN-A	ENN-A1	ENN-B	ENN-B1	FB1	FB2	FB3	HT-2	MON	NIV	OTA	OTB	T-2	ZEN
Lab 1	yes	-	-	-	-	-	-	-	-1.9	-	-	-	-1.8	-2.6	-1.1	0.9	-0.1	-0.3	-	1.7	-	1.7	-	-	-0.3
Lab 1	yes	-	-	-	-	-	-	-	-1.7	-	-	-	-1.7	-2.5	-1.0	1.0	0.5	-0.5	-	1.7	-	1.4	-	-	-0.4
Lab 2	no	0.3	-	1.1	no stat	0.7	-	-2.0	1.2	no stat	-1.7	0.0	1.0	0.2	1.7	-0.5	0.5	0.3	-	-1.4	2.1	-0.2	-	no stat	-1.4
Lab 2	no	0.5	-	2.7	no stat	0.4	-	-2.1	1.3	no stat	-1.5	-0.2	0.9	0.2	1.5	-0.5	0.2	1.0	-	-1.4	0.4	2.9	-	no stat	0.1
Lab 3	yes	-	-	-1.6	-	-	-	-	-	-	-1.3	-	-	-	-	-2.9	-2.2	-	no stat	-	-	-1.9	-	no stat	0.3
Lab 3	yes	-	-	-0.5	-	-	-	-	-	-	-1.3	-	-	-	-	-3.2	-2.5	-	no stat	-	-	-2.0	-	no stat	-0.7
Lab 4	yes	-	-	-0.9	-	-	-	1.4	-2.3	no stat	0.5	-	-	-3.1	-3.9	-1.5	-1.3	-0.8	-	0.8	0.2	-	-	-	0.1
Lab 4	yes	-	-	0.8	-	-	-	0.4	-2.7	no stat	0.9	-	-	-3.2	-4.0	-1.4	-1.3	-0.9	-	0.8	1.1	-	-	-	0.1
Lab 5	yes	-	-	1.7	-	-	-	6.0	-3.7	no stat	-0.2	-	-	-1.6	-1.6	2.2	0.9	1.0	-	-	-	0.0	-	-	-
Lab 5	yes	-	-	-0.7	-	-	-	10.7	-3.2	-	0.1	-	-	-1.9	-2.3	-0.3	0.6	0.0	-	-	-	-	-	-	-
Lab 6	no	-	-	0.4	-	0.9	-	-	1.9	-	1.3	0.3	1.0	7.3	3.4	6.7	2.4	-	-	-	-	4.6	-	-	2.7
Lab 6	no	-	-	-	-	16.2	-	-	1.5	-	1.5	0.6	1.5	5.8	2.4	9.0	9.0	-	-	-	-	6.3	-	-	9.0
Lab 7	yes	-	-	0.6	-	0.3	-	-	-	-	0.8	-	-	-	-	5.6	6.3	-	-	-	-	-	-	-	-
Lab 7	yes	-	-	1.1	-	1.1	-	-	-	-	1.3	-	-	-	-	4.1	3.8	-	-	-	-	-	-	-	-
Lab 8	no	-2.5	-2.9	-	-	-	-	-1.9	0.6	-	-3.5	-	-2.6	0.0	-1.4	-0.4	-1.8	-	no stat	-2.4	-1.7	-1.4	-	no stat	-1.6
Lab 8	no	-2.1	-3.1	-	-	-	-	-2.5	3.7	-	-3.5	-	-2.5	0.2	-1.2	-0.4	-1.9	-	no stat	0.0	-2.1	-1.3	-	-	-3.2
Lab 9	no	-	1.3	-0.7	-	-1.1	-	-1.0	0.8	-	0.4	0.1	0.9	1.3	1.3	-0.6	0.3	0.6	-	-	-	-3.5	-	-	1.1
Lab 9	no	-	1.3	-0.8	-	-1.3	-	-0.9	0.6	-	1.3	0.4	0.8	1.2	1.2	-0.8	-1.0	0.9	-	-	-	-3.2	-	-	0.8
Lab 10	no	1.5	0.5	-1.2	-	-1.8	-	1.1	1.0	-	0.6	-2.0	-0.8	2.0	2.0	-0.3	-0.5	-0.9	-	-	-	-	-	-	-0.1
Lab 10	no	1.7	0.2	-1.2	-	-1.8	-	1.1	1.9	-	1.2	-0.7	0.4	1.7	2.1	-0.1	-0.6	-0.6	-	-	-	-	-	-	0.1

Lot 8

Lab Code	ISTD	15-AcDON	3-AcDON	AFB1	AFB2	AFG1	AFG2	AOH	BEA	D3G	DON	ENN-A	ENN-A1	ENN-B	ENN-B1	FB1	FB2	FB3	HT-2	MON	NIV	OTA	OTB	T-2	ZEN
Lab 1	yes	-	-	-	-	-	-	-	-	-	2.8	-	-	-	-	-	-	-	-	2.9	-	-	-	-	-0.5
Lab 1	yes	-	-	-	-	-	-	-	-	-	2.9	-	-	-	-	-	-	-	-	2.7	-	-	-	-	-0.4
Lab 2	no	-1.4	-2.4	-	-	-	-	-	0.2	2.7	-1.3	-	-0.6	-0.7	-0.1	-	-	-	-	-2.1	-	-	-	-	0.8
Lab 2	no	0.1	-2.7	-	-	-	-	-	0.2	2.7	-1.0	-	-0.9	-0.2	0.3	-	-	-	-	-1.8	-	-	-	-	-2.5
Lab 3	yes	-	-	-	-	-	-	-	-	-	-1.2	-	-	-	-	no stat	no stat	-	-	-	-	-	-	-	-0.6
Lab 3	yes	-	-	-	-	-	-	-	-	-	-1.1	-	-	-	-	no stat	no stat	-	-	-	-	-	-	-	0.2
Lab 4	yes	0.8	-	-	-	-	-	-	1.1	0.0	1.0	-	-	-1.1	-3.0	-	-	-	-	0.3	-	-	-	-	0.0
Lab 4	yes	0.1	-	-	-	-	-	-	1.8	0.0	0.8	-	-	-0.8	-2.8	-	-	-	-	0.2	-	-	-	-	0.9
Lab 5	yes	6.0	-	-	-	-	-	-	-3.5	1.2	0.6	-	-	0.1	-	-	-	-	-	-	-	-	-	-	-
Lab 5	yes	3.8	-	-	-	-	-	-	-	-0.2	0.0	-	-	0.0	-	-	-	-	-	-	-	-	-	-	-
Lab 6	no	-	-	-	-	-	-	-	0.4	-	-1.4	no stat	2.0	18.1	5.3	-	no stat	-	-	-	-	-	-	-	-
Lab 6	no	-	-	-	-	-	-	-	1.4	-	-1.5	-	4.6	4.2	2.4	-	no stat	-	-	-	-	-	-	-	2.9
Lab 7	yes	-	-	-	-	-	-	-	-	-	1.5	-	-	-	-	-	-	-	-	-	-	-	-	-	-
Lab 7	yes	-	-	-	-	-	-	-	-	-	0.5	-	-	-	-	-	-	-	-	-	-	-	-	-	-
Lab 8	no	-3.7	-	-	-	-	-	-	-4.4	-	-4.3	-	-	-0.2	-	-	-	-	no stat	-1.3	-	-	-	-	-
Lab 8	no	-3.7	-	-	-	-	-	-	-4.1	-	-4.3	-	-	-	-	-	-	-	no stat	-0.6	-	-	-	-	-
Lab 9	no	0.0	2.5	-	-	-	-	no stat	1.2	-1.1	0.8	-	-0.2	0.3	-0.8	-	-	-	-	-	-	-	-	-	-0.6
Lab 9	no	0.1	1.8	-	-	-	-	no stat	0.3	-1.0	1.4	-	-0.2	0.3	-1.0	-	-	-	-	-	-	-	-	-	0.3
Lab 10	no	0.1	0.7	-	-	-	-	no stat	-0.7	-1.9	0.3	-	-	-0.2	0.5	-	-	-	-	-	-	-	-	-	-0.3
Lab 10	no	-0.4	0.1	-	-	-	-	no stat	-0.6	-1.6	0.1	-	-	0.1	0.8	-	-	-	-	-	-	-	-	-	0.0

Lab Code	ISTD	15-AcDON	3-AcDON	AFB1	AFB2	AFG1	AFG2	AOH	BEA	D3G	DON	ENN-A	ENN-A1	ENN-B	ENN-B1	FB1	FB2	FB3	HT-2	MON	NIV	OTA	OTB	T-2	ZEN
		**Lot 9**																							
Lab 1	yes	-	-	-	-	-	-	-	-	-	-	-	-	-	-	-	-	-	-	2.3	-	-	-	-	-
Lab 1	yes	-	-	-	-	-	-	-	-	-	-	-	-	-	-	-	-	-	-	2.5	-	-	-	-	-
Lab 2	no	4.4	-	-	-	-	-	-	3.5	0.5	-1.4	no stat	-0.3	0.3	0.6	-	-	-	-	-0.6	-	-	-	-	-2.7
Lab 2	no	1.5	-	-	-	-	-	-	-0.3	0.9	-1.6	no stat	0.3	0.3	1.3	-	-	-	-	-0.9	-	-	-	-	-2.8
Lab 3	yes	-	-	-	-	-	-	-	-	-	-1.6	-	-	-	-	no stat	-	-	-	-	-	no stat	-	-	-0.8
Lab 3	yes	-	-	-	-	-	-	-	-	-	-1.5	-	-	-	-	no stat	-	-	-	-	-	no stat	-	-	-1.6
Lab 4	yes	-	-	-	-	-	-	-	-0.1	1.2	1.1	-	-	-2.6	-3.8	-	-	-	-	0.4	-	-	-	-	0.8
Lab 4	yes	-	-	-	-	-	-	-	0.6	1.4	1.2	-	-	-2.3	-3.5	-	-	-	-	0.4	-	-	-	-	1.0
Lab 5	yes	-	-	-	-	-	-	-	-3.2	0.7	0.4	-	-	-0.9	-	-	-	-	-	-	-	-	-	-	-
Lab 5	yes	-	-	-	-	-	-	-	-2.4	0.1	-0.1	-	-	1.4	-	-	-	-	-	-	-	-	-	-	-
Lab 6	no	-	-	-	-	-	-	-	1.5	-	0.2	-	0.2	6.6	1.2	-	no stat	-	-	-	-	-	-	-	-
Lab 6	no	-	-	-	-	-	-	-	1.7	-	0.5	no stat	3.3	4.6	2.0	-	no stat	-	-	-	-	-	-	-	12.9
Lab 7	yes	-	-	-	-	-	-	-	-	-	1.9	-	-	-	-	-	-	-	-	-	-	-	-	-	-
Lab 7	yes	-	-	-	-	-	-	-	-	-	0.1	-	-	-	-	-	-	-	-	-	-	-	-	-	-
Lab 8	no	-2.8	no stat	-	-	-	-	-	-4.0	-	-3.3	-	-	-3.6	-	no stat	-	-	no stat	-1.6	-	no stat	no stat	-	-
Lab 8	no	-2.5	no stat	-	-	-	-	-	-	-	-3.1	-	-	-3.2	-	no stat	-	-	no stat	-2.3	-	no stat	no stat	-	-
Lab 9	no	-	-	-	-	-	-	-	0.5	-0.5	1.4	-	-0.4	0.9	-1.1	-	-	-	-	-	-	-	-	-	1.6
Lab 9	no	-	-	-	-	-	-	-	0.4	-1.1	1.1	-	-0.3	0.5	-0.8	-	-	-	-	-	-	-	-	-	0.9
Lab 10	no	0.3	-	-	-	-	-	-	-0.2	-1.9	0.9	-	-	0.3	0.7	-	-	-	-	-	-	-	-	-	0.2
Lab 10	no	-0.5	-	-	-	-	-	-	-0.3	-2.8	0.5	-	-	0.3	-0.1	-	-	-	-	-	-	-	-	-	-0.7

Lab Code	ISTD	15-AcDON	3-AcDON	AFB1	AFB2	AFG1	AFG2	AOH	BEA	D3G	DON	ENN-A	ENN-A1	ENN-B	ENN-B1	FB1	FB2	FB3	HT-2	MON	NIV	OTA	OTB	T-2	ZEN
		**Lot 10**																							
Lab 1	yes	-	-	-	-	-	-	-	-1.9	-	1.7	-	-	-2.5	-1.4	-	-	-	-	2.1	-	-	-	-	0.8
Lab 1	yes	-	-	-	-	-	-	-	-2.2	-	1.7	-	-	-2.7	-1.6	-	-	-	-	2.1	-	-	-	-	0.5
Lab 2	no	-0.5	-	-	-	-	-	-	1.0	0.5	-2.0	-0.2	1.7	0.0	1.3	no stat	-	-	3.3	-1.2	0.2	-	-	-	-1.1
Lab 2	no	1.5	-	-	-	-	-	-	0.5	0.4	-1.8	0.0	2.0	0.1	1.6	no stat	-	-	1.2	-0.4	1.0	-	-	-	-0.6
Lab 3	yes	-	-	-	-	-	-	-	-	-	-1.3	-	-	-	-	no stat	no stat	-	-0.6	-	-	no stat	-	no stat	-0.2
Lab 3	yes	-	-	-	-	-	-	-	-	-	-1.1	-	-	-	-	no stat	no stat	-	0.2	-	-	no stat	-	no stat	1.5
Lab 4	yes	-	-	-	-	-	-	-	-0.9	-0.4	0.2	-	-	-2.9	-3.7	-	-	-	-	0.1	1.0	-	-	-	0.6
Lab 4	yes	-	-	-	-	-	-	-	-0.6	-0.3	-0.1	-	-	-2.8	-3.6	-	-	-	-	0.2	1.1	-	-	-	0.4
Lab 5	yes	-	-	-	-	-	-	-	-2.4	1.6	1.5	-	2.5	0.9	3.0	-	-	-	-	-	-	-	-	-	-
Lab 5	yes	-	-	-	-	-	-	-	-0.9	1.0	0.0	-	-0.5	-0.5	-0.1	-	-	-	-	-	1.5	-	-	-	-
Lab 6	no	-	-	-	-	-	-	-	4.6	-	0.0	0.9	1.8	6.2	3.1	-	no stat	-	-	-	-	-	-	-	5.6
Lab 6	no	-	-	-	-	-	-	-	2.1	-	-0.2	0.9	2.3	5.2	1.9	-	-	-	-	-	-	-	-	-	3.9
Lab 7	yes	-	-	-	-	-	-	-	-	-	0.2	-	-	-	-	-	-	-	-	-	-	-	-	-	-
Lab 7	yes	-	-	-	-	-	-	-	-	-	-0.7	-	-	-	-	-	-	-	-	-	-	-	-	-	-
Lab 8	no	-3.7	-	-	-	-	-	-	0.3	-	-4.1	-	-3.2	1.8	-1.2	-	no stat	-	-1.5	-1.0	-2.1	-	-	-	-4.1
Lab 8	no	-3.7	-	-	-	-	-	-	0.4	-	-4.1	-	-3.1	2.1	-1.0	-	no stat	-	-1.7	-1.0	-2.1	-	-	-	-4.1
Lab 9	no	-0.1	no stat	-	-	-	-	no stat	0.2	-	2.1	-1.0	-0.8	-0.3	-0.4	-	-	-	-	-	-	-	-	-	-0.1
Lab 9	no	2.3	no stat	-	-	-	-	no stat	-0.5	-	1.2	-0.5	-0.2	0.0	-0.3	-	-	-	-	-	-	-	-	-	-0.2
Lab 10	no	1.5	no stat	-	-	-	-	no stat	0.5	-2.0	2.3	-	-1.0	0.3	0.9	-	-	-	-	-	0.0	-	-	-	-0.8
Lab 10	no	0.4	no stat	-	-	-	-	no stat	0.5	-2.8	2.5	-	-1.5	0.5	1.1	-	-	-	-	-	-1.3	-	-	-	-0.2

4. Overview of z-score deviations

Table S10: Overview of analyte specific z-score deviations from ± 2 per laboratory and matrix

Analyt e	Soy				Glute n				Chicke n				Swine				sp of dev in %
	≥ 6 dp	< -2	> 2	% dev	≥ 6 dp	< -2	> 2	% dev	≥ 6 dp	< -2	> 2	% dev	≥ 6 dp	< -2	> 2	% dev	
Aflatoxin B1	-	-	-	-	3	-	-	0	3	-	-	0	1	-	-	0	0
Aflatoxin B2	-	-	-	-	-	-	-	-	-	-	-	-	-	-	-	-	-
Aflatoxin G1	-	-	-	-	2	-	-	0	-	-	-	-	-	-	-	-	0
Aflatoxin G2	-	-	-	-	-	-	-	-	-	-	-	-	-	-	-	-	-
Deoxynivalenol	-	-	-	-	10	-	3	30	10	1	4	50	10	1	1	20	33
Fumonisin B1	-	-	-	-	10	2	2	40	10	1	1	20	10	1	2	30	30
Fumonisin B2	-	-	-	-	10	1	1	20	8	1	2	38	8	1	2	38	31
HT-2 toxin	-	-	-	-	8	1	1	25	4	-	-	0	3	-	1	33	20
Ochratoxin A	-	-	-	-	9	1	1	22	6	1	2	50	7	1	1	29	32
T-2 toxin	-	-	-	-	10	-	1	10	8	-	1	13	6	-	1	17	13
Zearalenone	7	-	2	29	10	1	1	20	10	-	-	0	10	1	1	20	16
15-Acetyl-Deoxynivalenol	-	-	-	-	6	1	2	50	4	-	-	0	6	1	2	50	38
3-Acetyl-Deoxynivalenol	-	-	-	-	3	1	-	33	1	-	-	0	4	1	-	25	25
Alternariol	-	-	-	-	7	1	2	43	6	1	2	50	6	2	1	50	47
Beauvericin	6	1	1	33	7	1	2	43	8	1	2	38	8	1	-	13	31
Deoxynivalenol-3-Glucoside	-	-	-	-	1	-	-	0	3	-	-	0	5	-	-	0	0
Enniatin A	-	-	-	-	2	-	-	0	2	-	-	0	5	1	-	20	11
Enniatin A 1	4	-	-	0	4	1	-	25	6	1	1	33	8	1	-	13	18
Enniatin B	6	1	1	33	7	-	2	29	7	1	2	43	8	1	1	25	32
Enniatin B1	5	-	1	20	6	1	-	17	8	1	1	25	8	1	1	25	22
Fumonisin B3	-	-	-	-	6	-	-	0	3	-	-	0	2	-	-	0	0
Moniliformin	-	-	-	-	4	-	-	0	4	-	-	0	4	-	1	25	8
Nivalenol	-	-	-	-	-	-	-	-	1	-	-	0	4	-	-	0	0
Ochratoxin B	-	-	-	-	1	-	-	0	-	-	-	-	1	-	-	0	0

The table includes an overview of possible average z-score data evaluable per matrix. This calculation is based on the number of z-scores calculated in this study. Each laboratory (in total 10) which provided ≥ 6 z-score data points (≥ 6 dp) per matrix was taking into account, resulting in a maximum of 10 possible data points per analyte/matrix combination. In addition, information about tendencies in under (< -2) and overestimations (> 2) per analyte are provided. Based on that informational content, the total share of deviations in percent (% dev) were calculated for each substance. Finally, a sum product was derived in percent (sp of dev in %) for each compound in order to reflect a weighted average value for the interpretation of the analyte specific performance. This information acts as the basis for the interpretation of matrix independent tendencies in deviations for the total scope of this study. Average deviations lower 25% are marked as green, data above as yellow indicating general problem with this compound in all matrices.

5. Regulatory framework of the European Union

Table S11: Overview of EU regulated mycotoxins

Mycotoxin	Regulated Commodities	Maximum Levels Food	Maximum Levels Feed
Aflatoxins<br>- AFB1<br>- AFB2<br>- AFG1<br>- AFG2	Groundnuts, almonds, pistachios, brazil nuts, tree nuts, dried fruit, corn, rice, cereals, milk, apricot kernels, infant formula, dried figs, all feed materials	0.05 - 15 µg/kg	5 - 20 µg/kg
Citrinin	Food supplements (based on rice fermented with red yeast)	2000 µg/kg	
Deoxynivalenol	Unprocessed cereals, durum wheat, oats and corn, cereal flour, corn flour, corn, grits, corn meal, bread biscuits, pastries, cereal snacks, breakfast cereals, dry pasta, processed cereal based baby and infant food, cereals and cereal products, corn by-products, complementary and complete feeding stuffs	200 - 1250 µg/kg	0.9 - 12 mg/kg
Ergot alkaloids	Milling products of barley, wheat, spelt and oats, barley, wheat, spelt, oat grains and rye, rye milling products, wheat gluten, processed cereal based food for infants and young chicken, feed materials containing unground cereal	20 - 500 µg/kg	1000 mg/kg
Fumonisins<br>- FB1<br>- FB2	Unprocessed corn, corn, corn based foods, breakfast cereals and snacks, processed corn based foods and baby foods for infants and young children, complementary and complete feeding stuffs	200 - 4000 µg/kg	5 - 60 mg/kg
Ochratoxin A	Unprocessed cereals and all products derived from them, dried wine fruit, wine, fruit wine, flavored wine, grape juice, roasted coffee beans, ground roasted coffee, soluble coffee, species of spices, mixtures of sices, liquorice root and extract, baby foods and processed ceral based foods for infants and young children, dietary foods for infants, wheat gluten, complementary and complete feeding stuffs	0.5 - 80 µg/kg	0.05 - 0.25 mg/kg
Patulin	Fruit juices and fruit nectar containing apple juice or derived from apples, spirit drinks, cider, fermented drinks from apples, solid apple products, apple puree, apple juice and products for infants and young children	10 - 50 µg/kg	-
T2 Toxin & HT2-Toxin	Unprocessed cereals, cereal grains for direct human consumption, cereal products for human consumtion, cereal products for feed and compound feed	15 - 1000 µg/kg	50 - 2000 µg/kg
Zearalenone	Unprocessed cereals and corn, cereals, corn and bran intended for direct human consumption, cereal flour, corn based snacks and cereals, refined corn oil, bread, pastries, biscuits, cereal snacks, breakfast cereals, processed cereal and corn based foods and baby foods for infants and young children	20 - 400 µg/kg	0.1 - 2 mg/kg

6. Control Standard Solutions

Table S12: Preparation scheme of the control solutions provided by the study organizer. The participants were informed to dilute the standard mixtures (conc. mix) of each vial in a ratio of 1:10 by using the same solvent as for their calibration standards.

VIAL 1						
analyte	conc. stock µg/mL	Lot no.	dilution factor	µL standard	conc. mix [µg/L]	target conc. [µg/L]
aflatoxin B1	1.04	10002879	50	160	20.8	2.08
aflatoxin B2	1.03				20.6	2.06
aflatoxin G1	1.01				20.2	2.02
aflatoxin G2	1.03				20.6	2.06
HT-2 toxin	100	10000352	200	40	2000	200
T-2 toxin	100	10003663		40	2000	200
ochratoxin A	10.03	10003658	500	16	201	20.1
zearalenone	100	10003660		16	2000	200
deoxynivalenol	100	10000334	100	80	2000	200
			sum [µL]:	352		
			ACN [µL]:	7648		
			total [µL]:	8000		

VIAL 2						
analyte	conc. stock µg/mL	Lot no.	dilution factor	µL standard	conc. mix [µg/L]	target conc. [µg/L]
fumonisin B1	50.1	10003642	50	160	1002	100
fumonisin B2	50.0	10003643		160	1000	100
fumonisin B3	50.9	10003645		160	1018	102
			sum [µL]:	480		
			ACN/H_2O (50/50) [µL]:	7520		
			total [µL]:	8000		

7. Lab specific methodology

Table S13: Lab 001 method information

Scope	LOQ (µg/kg)	Quantifier transition(m/z > m/z)	Qualifier transition (m/z > m/z)	Pig Feed Recovery value (%)	Soy Bean Recovery value (%)	Chicken Feed Recovery value (%)	Corn Gluten Recovery value (%)
**Mycotoxins (mandatory)**							
aflatoxin B1	3	312.9 : 285.0	312.9 : 240.9	99.8	100	100	100
aflatoxin B2	3	315.0 : 287.0	315.0 : 258.9	98.2	100	100	100
aflatoxin G1	3	329.0 : 242.9	329.0 : 200.0	103	100	100	100
aflatoxin G2	3	331.0 : 245.0	331.0 : 115.2	102	100	100	100
deoxynivalenol	500	355.0 : 58.9	355.0 : 294.9	106	100	100	100
fumonisin B1	15	722.5 : 334.2	722.5 : 352.2	60.0	100	100	100
fumonisin B2	15	706.4 : 336.2	706.4 : 318.2	72.6	100	100	100
HT-2 toxin	50	442.2 : 263.1	442.2 : 215.0	112	100	100	100
ochratoxin A	2	404.1 : 238.9	404.1 : 358.1	98.4	100	100	100
T-2 toxins	3	484.3 : 305.0	484.3 : 245.1	109	100	100	100
zearalenone	5	316.6 : 130.9	316.6 : 174.8	111	100	100	100
**Mycotoxins (voluntary)**							
15-acetyl-deoxynivalenol	1000	356.2 : 145.0	356.2 : 338.2	112	100	100	100
3-acetyl-deoxynivalenol	150	397.0 : 58.9	397.0 : 336.9	107	100	100	100
alternariol	15	257.0 : 212.8	257.0 : 214.8	51.3	83.1	70.6	63.4
beauvericin	4	801.4 : 784.3	801.4 : 244.3	115	88.8	65.7	
deoxynivalenol-3-glucoside	350	517.2 : 456.9	517.2 : 59.0	79.4	100	100	100
enniatin A	3	699.1 : 682.7	699.1 : 210.4	111	96.1	78.7	81.5
enniatin A1	3	685.5 : 668.4	685.5 : 210.1	127	88.0	63.1	57.6
enniatin B	3	657.1 : 640.5	657.1 : 196.0	104			
enniatin B1	3	671.0 : 654.3	671.0 : 196.4	123	96.7	90.4	86.4
fumonisin B3	15	706.5 : 336.2	706.5 : 81.1	70.9	100	100	100
moniliformin	10	96.9 : 40.9		70.2	149	137	115
nivalenol	1000	371.1 : 280.8	371.1 : 59.0	90.9	100	100	100
ochratoxin B	2	370.1 : 205.0	370.1 : 187.1	82.6	39.3	40.3	35.9

Table S14: Lab 002 method information

Scope	LOQ (µg/kg)	Quantifier transition(m/z > m/z)	Qualifier transition (m/z > m/z)	Pig Feed Recovery value (%)	Soy Bean Recovery value (%)	Chicken Feed Recovery value (%)	Corn Gluten Recovery value (%)
**Mycotoxins (mandatory)**							
aflatoxin B1	0.72	313 > 285	313 > 128	41	49	36	27
aflatoxin B2	0.21	315 > 287	315 > 259	44	49	43	36
aflatoxin G1	0.54	329 > 243	329 > 200	43	45	43	30
aflatoxin G2	1.69	331 > 313	331 < 245	43	45	43	30
deoxynivalenol	3.00	355 > 265	355 > 59	80	73	80	60
fumonisin B1	7.98	72 > 334	722 > 352	66	18	59	70
fumonisin B2	7.08	706 > 336	706 > 318	75	58	68	70
HT-2 toxin	5.00	442 > 263	442 > 215	77	85	74	43
ochratoxin A	1.50	404 < 239	404 > 102	92	93	85	66
T-2 toxins	2.40	484 > 215	484 > 185	76	87	77	56
zearalenone	0.63	317 > 175	317 > 131	84	39	84	56
**Mycotoxins (voluntary)**							
15-acetyl-deoxynivalenol	15.0	339 > 321	339 > 137	86	66	80	66
3-acetyl-deoxynivalenol	15.0	397 > 59	397 > 307	58	58	59	58
alternariol	0.32	257 > 213	257 > 215	57	46	52	51
beauvericin	0.10	801 > 244	801 > 134	100	100	100	100
deoxynivalenol-3-glucoside	3.00	517 > 427	517 > 59	80	80	80	66
enniatin A	0.03	699 > 210	699 < 228	79	79	79	79
enniatin A1	0.20	685 > 210	685 < 228	91	91	91	91
enniatin B	0.04	657 > 196	657 > 214	91	91	91	91
enniatin B1	0.11	671 > 196	671 > 210	82	82	82	82
fumonisin B3	7.00	706 > 336	706 > 318	75	58	68	70
moniliformin	5.00	96.9 > 41.2		98	99	101	100
nivalenol	2.60	371 > 281	371 > 59	80	80	80	66
ochratoxin B	0.36	380 > 205	370 > 203	82	82	82	65

Table S15: Lab 003 method information

Scope	LOQ (µg/kg)	Quantifier transition(m/z > m/z)	Qualifier transition (m/z > m/z)	Pig Feed Recover y value (%)	Soy Bean Recover y value (%)	Chicken Feed Recovery value (%)	Corn Gluten Recovery value (%)
**Mycotoxins (mandatory)**							
aflatoxin B1	0.2	313,1>285	313,1>241				
aflatoxin B2	0.2	315,1>258,9	315,1>287				
aflatoxin G1	0.2	329,1>243	329,1>200,1				
aflatoxin G2	0.2	331,1>245,1	331,1>313				
deoxynivalenol	40	297,1>249	297,1>203				
fumonisin B1	100	722,4>334,4	722,4>352,4				
fumonisin B2	100	706,4>336,4	706,4>318,3				
HT-2 toxin	5	442,2>263	442,2>215				
ochratoxin A	2	404,1>238,9	404,1>102,1				
T-2 toxins	5	484,3>305	484,3>215,1				
zearalenone	20	317,1>272,9	317,1>130,9				
**Mycotoxins (voluntary)**							
15-acetyl-deoxynivalenol							
3-acetyl-deoxynivalenol							
alternariol							
beauvericin							
deoxynivalenol-3-glucoside							
enniatin A							
enniatin A1							
enniatin B							
enniatin B1							
fumonisin B3							
moniliformin							
nivalenol							
ochratoxin B							

Table S16: Lab 004 method information

Scope	LOQ (µg/kg)	Quantifier transition(m/z > m/z)	Qualifier transition(m/z > m/z)	Pig Feed Recovery value (%)	Soy Bean Recovery value (%)	Chicken Feed Recovery value (%)	Corn Gluten Recovery value (%)
**Mycotoxins (mandatory)**							
aflatoxin B1		312.9 : 285.0	312.9 : 240.9				
aflatoxin B2		315.0 : 287.0	315.0 : 258.9				
aflatoxin G1		329.0 : 242.9	329.0 : 200.0				
aflatoxin G2		331.0 : 245.0	331.0 : 115.2				
deoxynivalenol		355.0 : 58.9	355.0 : 294.9				
fumonisin B1		722.5 : 334.2	722.5 : 352.2				
fumonisin B2		706.4 : 336.2	706.4 : 318.2				
HT-2 toxin		442.2 : 263.1	442.2 : 215.0				
ochratoxin A		404.1 : 238.9	404.1 : 358.1				
T-2 toxins		484.3 : 305.0	484.3 : 245.1				
zearalenone		316.6 : 130.9	316.6 : 174.8				
**Mycotoxins (voluntary)**							
15-acetyl-deoxynivalenol		356.2 : 145.0	356.2 : 338.2				
3-acetyl-deoxynivalenol		397.0 : 58.9	397.0 : 336.9				
alternariol		257.0 : 212.8	257.0 : 214.8				
beauvericin		801.4 : 784.3	801.4 : 244.3				
deoxynivalenol-3-glucoside		517.2 : 456.9	517.2 : 59.0				
enniatin A		699.1 : 682.7	699.1 : 210.4				
enniatin A1		685.5 : 668.4	685.5 : 210.1				
enniatin B		657.1 : 640.5	657.1 : 196.0				
enniatin B1		671.0 : 654.3	671.0 : 196.4				
fumonisin B3		706.5 : 336.2	706.5 : 81.1				
moniliformin		96.9 : 40.9					
nivalenol		371.1 : 280.8	371.1 : 59.0				
ochratoxin B		370.1 : 205.0	370.1 : 187.1				

Table S17: Lab 005 method information

Scope	LOQ (µg/kg)	Quantifier transition(m/z > m/z)	Qualifier transition (m/z > m/z)	Pig Feed Recovery value (%)	Soy Bean Recovery value (%)	Chicken Feed Recovery value (%)	Corn Gluten Recovery value (%)
**Mycotoxins (mandatory)**							
aflatoxin B1	1	312.9 : 285.0	312.9 : 240.9	102	102	111	115
aflatoxin B2	2	315.0 : 287.0	315.0 : 258.9	99	109	101	88
aflatoxin G1	2	329.0 : 242.9	329.0 : 200.0	102	82	109	93
aflatoxin G2	2	331.0 : 245.0	331.0 : 115.2	99	96	91	89
deoxynivalenol	50	355.0 : 58.9	355.0 : 294.9	93	93	96	94
fumonisin B1	30	722.5 : 334.2	722.5 : 352.2	63	39	76	70
fumonisin B2	30	706.4 : 336.2	706.4 : 318.2	70	43	70	70
HT-2 toxin	50	442.2 : 263.1	442.2 : 215.0	87	98	95	115
ochratoxin A	2	404.1 : 238.9	404.1 : 358.1	92	92	97	94
T-2 toxins	30	484.3 : 305.0	484.3 : 245.1	99	104	113	125
zearalenone	10	316.6 : 130.9	316.6 : 174.8	90	113	104	103
**Mycotoxins (voluntary)**							
15-acetyl-deoxynivalenol	100	356.2 : 145.0	356.2 : 338.2	90	94	96	94
3-acetyl-deoxynivalenol	100	397.0 : 58.9	397.0 : 336.9	102	136	108	111
alternariol	10	257.0 : 212.8	257.0 : 214.8	93	105	95	87
beauvericin	6	801.4 : 784.3	801.4 : 244.3	95	98	86	89
deoxynivalenol-3-glucoside	50	517.2 : 456.9	517.2 : 59.0	79	84	87	86
enniatin A	3	699.1 : 682.7	699.1 : 210.4	83	101	96	50
enniatin A1	3	685.5 : 668.4	685.5 : 210.1	91	107	96	78
enniatin B	3	657.1 : 640.5	657.1 : 196.0	97	97	104	76
enniatin B1	3	671.0 : 654.3	671.0 : 196.4	100	104	91	88
fumonisin B3	30	706.5 : 336.2	706.5 : 81.1	72	43	74	68
moniliformin	20	96.9 : 40.9		98	117	92	89
nivalenol	60	371.1 : 280.8	371.1 : 59.0	85	100	82	72
ochratoxin B	3	370.1 : 205.0	370.1 : 187.1	86	87	87	114

Table S18: Lab 006 method information

Scope	LOQ (µg/kg)	Quantifier transition(m/z > m/z)	Qualifier transition (m/z > m/z)	Pig Feed Recovery value (%)	Soy Bean Recovery value (%)	Chicken Feed Recovery value (%)	Corn Gluten Recovery value (%)
**Mycotoxins (mandatory)**							
aflatoxin B1	1.2	313.1 > 285.1	313.1 > 213.1				
aflatoxin B2	1.2	315.1 > 287.1	315.1 > 259.0				
aflatoxin G1	0.4	329.1 > 243.1	329.1 > 200.0				
aflatoxin G2	1.2	331.0 > 189.1	331.1 > 245.1				
deoxynivalenol	80	355.1 > 265.0	355.1 > 247.2				
fumonisin B1	120	722.5 > 352.3	722.5 > 334.4				
fumonisin B2	40	706.4 > 336.3	706.4 > 318.2				
HT-2 toxin	80	442.2 > 215.0	442.5 > 215.0				
ochratoxin A	4.8	404.1 > 239.0	404.1 > 101.9				
T-2 toxins	4.8	484.3 > 215.1	484.1 > 185.1				
zearalenone	4	317.1 > 175.1	317.1 > 131.1				
**Mycotoxins (voluntary)**							
15-acetyl-deoxynivalenol							
3-acetyl-deoxynivalenol							
alternariol	12	257.1 > 215.1	257.1 > 147.0				
beauvericin	0.24	801.4 > 243.9	801.4 > 133.9				
deoxynivalenol-3-glucoside							
enniatin A	0.24	699.5 > 210.0	600.5 > 228.0				
enniatin A1	0.24	685.5 > 210.0	685.5 > 228.2				
enniatin B	0.24	657.4 > 196.3	657.4 > 213.9				
enniatin B1	0.024	671.4 > 196.0	671.4 > 209.9				
fumonisin B3							
moniliformin							
nivalenol	300	371.0 > 311.22	371.0 > 281.1				
ochratoxin B	1.2	370.1 > 205.0	370.0 > 103.0				

Table S19: Lab 007 method information

Scope	LOQ (μg/kg)	Quantifier transition(m/z > m/z)	Qualifier transition (m/z > m/z)	Pig Feed Recovery value (%)	Soy Bean Recovery value (%)	Chicken Feed Recovery value (%)	Corn Gluten Recovery value (%)
**Mycotoxins (mandatory)**							
aflatoxin B1	0.75	313	285	84		84	
aflatoxin B2	0.75	315	287	85		85	
aflatoxin G1	0.75	328	243	91		91	
aflatoxin G2	0.75	331	313	96		96	
deoxynivalenol	50	355	265	90		90	
fumonisin B1	25	722	352	80		80	
fumonisin B2	25	706	336	82		82	
HT-2 toxin	10	442	263	75		75	
ochratoxin A	5	404	239	96		96	
T-2 toxins	10	489	327	73		73	
zearalenone	25	317	175	86		86	
**Mycotoxins (voluntary)**							
15-acetyl-deoxynivalenol							
3-acetyl-deoxynivalenol							
alternariol							
beauvericin							
deoxynivalenol-3-glucoside							
enniatin A							
enniatin A1							
enniatin B							
enniatin B1							
fumonisin B3							
moniliformin							
nivalenol							
ochratoxin B							

Table S20: Lab 008 method information

Scope	LOQ (µg/kg)	Quantifier transition(m/z > m/z)	Qualifier transition (m/z > m/z)	Pig Feed Recovery value (%)	Soy Bean Recovery value (%)	Chicken Feed Recovery value (%)	Corn Gluten Recovery value (%)
**Mycotoxins (mandatory)**							
aflatoxin B1		313.061 > 285.1	313.061 > 241.1				
aflatoxin B2		315.074 > 287.2	315.074 > 259.1				
aflatoxin G1		329.055 > 243.2	329.055 > 311.1				
aflatoxin G2		331.057 > 313.0	331.057 > 245.2				
deoxynivalenol		297.097 > 249.1	297.097 > 203.2				
fumonisin B1		722.316 > 704.3	722.316 > 334.4				
fumonisin B2		706.309 > 354.3	706.309 > 336.1				
HT-2 toxin		442.257 > 263.102	442.257 > 215.102				
ochratoxin A		404.092 > 239.0	404.092 > 358.1				
T-2 toxins		484.3 > 215.2	484.3 > 185.1				
zearalenone		317.1 > 131.1	317.1 > 175.0				
**Mycotoxins (voluntary)**							
15-acetyl-deoxynivalenol		339.1 > 321.3	339.1 > 261.1				
3-acetyl-deoxynivalenol		397.3 > 59.2	397.3 > 307.1				
alternariol		256.957 > 213.0	256.957 > 215.0				
beauvericin		801.287 > 784.3	801.287 > 244.1				
deoxynivalenol-3-glucoside							
enniatin A		699.386 > 682.4	699.386 > 210.2				
enniatin A1		685.36 > 668.5	685.36 > 210.1				
enniatin B		657.319 > 640.3	657.319 > 196.1				
enniatin B1		671.317 > 654.4	671.317 > 196.1				
fumonisin B3							
moniliformin		96.946 > 41.2					
nivalenol		371.094 > 281.1	371.094 > 59.1				
ochratoxin B		370.08 > 205.0	370.08 > 187.1				

Table S21: Lab 009 method information

Scope	LOQ (µg/kg)	Quantifier transition (m/z > m/z)	Qualifier transition (m/z > m/z)	Pig Feed Recovery value (%)	Soy Bean Recovery value (%)	Chicken Feed Recovery value (%)	Corn Gluten Recovery value (%)
**Mycotoxins (mandatory)**							
aflatoxin B1	0.5	313 > 285	313 > 241			84	
aflatoxin B2	0.5	315 > 287	315 > 259			84	
aflatoxin G1	0.5	329 > 243	329 > 200			85	
aflatoxin G2	0.5	331 > 313	331 > 189			78	
deoxynivalenol	100	355 > 265	355 > 295			72	
fumonisin B1	50	722 > 334	722 > 352			88	
fumonisin B2	50	706 > 336	706 > 318			94	
HT-2 toxin	10	442 > 263	442 > 215			79	
ochratoxin A	1	404 > 239	404 > 102			84	
T-2 toxins	1	484 > 305	484 > 185			92	
zearalenone	0.5	317 > 175	317 > 131			82	
**Mycotoxins (voluntary)**							
15-acetyl-deoxynivalenol	25	356 > 137	356 > 321			80	
3-acetyl-deoxynivalenol	25	397 > 59	397 > 337			93	
alternariol	0.5	257 > 213	257 > 215			91	
beauvericin	2.5	801 > 784	801 > 262			71	
deoxynivalenol-3-glucoside	100	517 > 457	517 > 427			30	
enniatin A	0.5	699 > 210	699 > 228			92	
enniatin A1	0.5	685 > 210	685 > 214			90	
enniatin B	0.5	657 > 196	657 > 214			90	
enniatin B1	0.5	671 > 214	671 > 228			90	
fumonisin B3	50	706 > 336	706 > 318			74	
moniliformin							
nivalenol	100	371 > 281	371 > 311			72	
ochratoxin B	0.5	370 > 205	370 > 187			91	

Table S22: Lab 010 method information

Scope	LOQ (µg/kg)	Exact mass m/z (precursor)	Exact mass m/z (fragmemt)	Pig Feed Recover y value (%)	Soy Bean Recover y value (%)	Chicken Feed Recovery value (%)	Corn Gluten Recovery value (%)
**Mycotoxins (mandatory)**							
aflatoxin B1	0.5	313.0706646	241.0495353			82	
aflatoxin B2	0.5	315.0863146	259.0600999			83	
aflatoxin G1	0.5	329.0655792	215.0702707			80	
aflatoxin G2	0.5	331.0812292	245.0808354			77	
deoxynivalenol	50	355.1398409	265.1081469			73	
fumonisin B1	50	722.3957464	334.3104414			99	
fumonisin B2	50	706.4008318	336.3260915			101	
HT-2 toxin	1	442.2435435	263.1277856			86	
ochratoxin A	1	404.0895415	358.0840622			84	
T-2 toxins	0.5	484.2541081	305.1383502			83	
zearalenone	0.5	317.139447	131.0502382			84	
**Mycotoxins (voluntary)**							
15-acetyl-deoxynivalenol	5	356.1703786	339.1438295			72	
3-acetyl-deoxynivalenol	10	397.1504056	59.0138			90	
alternariol	0.5	257.0455467	215.034982			82	
beauvericin	1	801.4433058	244.1332053			84	
deoxynivalenol-3-glucoside	50	517.1926642	427.1609702			30	
enniatin A	1	699.490256	210.1488554			90	
enniatin A1	2.5	685.474606	668.4480569			87	
enniatin B	0.5	657.4433058	196.1332053			89	
enniatin B1	0.5	671.4589559	196.1332053			85	
fumonisin B3	50	706.4008318	336.3260915			100	
moniliformin							
nivalenol	25	371.1347555	281.1030615			71	
ochratoxin B	0.5	370.1285	205.0495			88	
